# High-Throughput Identification of Potential Minor Histocompatibility Antigens by MHC Tetramer-Based Screening: Feasibility and Limitations

**DOI:** 10.1371/journal.pone.0022523

**Published:** 2011-08-05

**Authors:** Pleun Hombrink, Sine R. Hadrup, Arne Bakker, Michel G. D. Kester, J. H. Frederik Falkenburg, Peter A. von dem Borne, Ton N. M. Schumacher, Mirjam H. M. Heemskerk

**Affiliations:** 1 Department of Hematology, Leiden University Medical Center, Leiden, The Netherlands; 2 Division of Immunology, The Netherlands Cancer Institute, Amsterdam, The Netherlands; University Paris Sud, France

## Abstract

T-cell recognition of minor histocompatibility antigens (MiHA) plays an important role in the graft-versus-tumor (GVT) effect of allogeneic stem cell transplantation (allo-SCT). However, the number of MiHA identified to date remains limited, making clinical application of MiHA reactive T-cell infusion difficult. This study represents the first attempt of genome-wide prediction of MiHA, coupled to the isolation of T-cell populations that react with these antigens. In this unbiased high-throughput MiHA screen, both the possibilities and pitfalls of this approach were investigated. First, 973 polymorphic peptides expressed by hematopoietic stem cells were predicted and screened for HLA-A2 binding. Subsequently a set of 333 high affinity HLA-A2 ligands was identified and post transplantation samples from allo-SCT patients were screened for T-cell reactivity by a combination of pMHC-tetramer-based enrichment and multi-color flow cytometry. Using this approach, 71 peptide-reactive T-cell populations were generated. The isolation of a T-cell line specifically recognizing target cells expressing the MAP4K1_IMA_ antigen demonstrates that identification of MiHA through this approach is in principle feasible. However, with the exception of the known MiHA HMHA1, none of the other T-cell populations that were generated demonstrated recognition of endogenously MiHA expressing target cells, even though recognition of peptide-loaded targets was often apparent.

Collectively these results demonstrate the technical feasibility of high-throughput analysis of antigen-specific T-cell responses in small patient samples. However, the high-sensitivity of this approach requires the use of potential epitope sets that are not solely based on MHC binding, to prevent the frequent detection of T-cell responses that lack biological relevance.

## Introduction

Patients with hematological malignancies can be successfully treated with HLA-matched allogeneic stem cell transplantation (allo-SCT) and subsequent donor lymphocyte infusion (DLI) [1], [2]. The graft-versus-leukemia (GVL) effect of this successful immunotherapy is due to recognition by donor T-cells of minor histocompatibility antigens (MiHA) expressed on malignant hematopoietic recipient cells [3]–[6]. These MiHA result from genetic polymorphisms between donor and recipient that alter the HLA-associated peptide repertoire, and are therefore capable to elicit a potent T-cell response in the context of self-HLA [7]. Unfortunately, most MiHA are not solely expressed on hematopoietic cells but display a broad expression pattern in nonmalignant recipient tissues. As a consequence, DLI can induce or enhance graft-versus-host disease (GVHD), one of the main causes of transplant-related morbidity and mortality [8], [9]. It is assumed that the selective infusion of T-cells reactive with MiHA exclusively expressed on recipient hematopoietic cells would help to separate the beneficial GVL effect from GVHD, and identification of MiHA with a hematopoietic expression pattern is therefore of interest.

To date, the number of known MiHA that form attractive targets for antigen-selective cell therapy remains limited. As a consequence of the requirement for both the relevant MiHA mismatch between donor and recipient and expression of the relevant HLA restriction molecule, the percentage of patients that can be treated with such MiHA-selective cell therapy remains low [10]. Considering the complex gene expression profiles in hematopoietic cells [11] and the enormous number of known allelic polymorphisms [12], the existence of many more clinically applicable MiHA seems reasonable.

Several biochemical and molecular methods have successfully led to the identification of MiHA including peptide elution from HLA, cDNA library screening, genetic linkage analysis, and genome-wide association analysis [7], [13]–[16]. These methods identified MiHA using a forward immunology approach, based on the characterization of epitopes recognized by T-cells isolated during a GVL response.

The use of soluble fluorescently labeled multimeric peptide-MHC (pMHC) complexes has become a widely used approach to detect antigen-specific T-cells in a diverse T-cell repertoire [17]. Furthermore, the development of technology for high-throughput pMHC production [18], [19], makes it possible to also screen for T-cell reactivity against large panels of potential antigens by flow cytometry either by combinatorial encoding [20], or by extension of the number of fluorescent labels used for pMHC tetramer labeling [21], [22].

In this study we set out to determine whether genome-wide identification of MiHA by pMHC-tetramer screening is feasible. In addition, we assessed whether these screens are possible in an unbiased approach, in which patient are screened with a fixed set of pMHC tetramers. To this purpose, we first predicted a large number of potential MiHA epitopes using HLA-peptide binding algorithms, single nucleotide polymorphism (SNP) data and gene-expression databases. Subsequently, flow cytometry-based high-throughput analysis of antigen-specific T-cell responses, followed by functional testing of identified T-cell clones was used to assess the clinical value of predicted MiHA. This unbiased screen resulted in the generation of a large number of pMHC tetramer positive T-cell lines. Subsequent functional analysis demonstrated the isolation of two high-affinity T-cell populations specific for the known HMHA-1 MiHA as well as a previously unknown epitope. However, since this novel epitope was not produced to a sufficient level by the endogenous antigen presentation machinery, it should not be considered a bona fide MiHA.

Two major conclusions can be drawn from our study: First, high-throughput analysis of antigen-specific T-cell responses in small patient samples is technically feasible using the highly sensitive technologies developed here. Second, when such screens are performed using unbiased peptide sets that are based on epitope binding, irrespective of peptide processing data and SNP status of donor and recipient, the vast majority of T-cell responses detected are of insufficient avidity to allow recognition of endogenously produced antigen, or are directed against epitopes that are not naturally presented to a sufficient extent to allow T-cell recognition.

## Results

### Identification of genes with a hematopoiesis-restricted expression pattern

In many hematological malignancies it is likely to be essential to therapeutically target not only the differentiated leukemic cells, but also the leukemic stem cell fraction, because of this, genes that are expressed in hematopoietic precursor cells are of interest as a potential source of MiHA, as these genes are likely to be expressed in leukemic precursor cells as well. To obtain a better insight into the gene expression profiles of specific hematopoietic cell fractions, we performed microarray analyses on hematopoietic stem cells purified from bone marrow, G-CSF mobilized peripheral blood and cord blood. Both CD34^+^/CD38^−^ and CD34^+^/CD38^+^ fractions were analyzed, representing early and more committed hematopoietic stem cells, respectively. Subsequently, these data were merged with gene expression data for similar and other cell types from the NCBI GEO database [23], to identify genes expressed in stem cells with a hematological restricted pattern. The robustness of the approach was shown by the identification of known hematopoiesis-restricted MiHA encoding genes such as HMHA-1 and PTPRC (CD45). In addition, ubiquitous and non-hematopoiesis tissue specific genes like KLK2 and GAPD, were also found to have the expected expression profiles, demonstrating that this combined database was sufficiently robust to identify genes with a hematopoiesis-restricted expression pattern (e.g. ITGB2 and FLT3 (Figure S1a–f). The combined database was subsequently used to identify 79 non-Y-chromosomal genes that are relatively specifically expressed in hematopoiesis-restricted cell subsets (Table S1).

### SNP identification in selected genes

The molecular basis for the immunogenicity of most MiHA is formed by amino acid changes in MHC-restricted epitopes that occur as a consequence of single nucleotide polymorphisms. Identification of such SNPs within our 79 hematopoiesis-restricted genes using the NCBI's dbSNP polymorphism database [24] revealed 315 SNPs, of which the majority was nonsynonymous. In addition, as MiHA have been reported to also be encoded by alternative reading frames [5], [6] (ARF) we also included synonymous SNPs to ensure no MiHA encoded by ARF were left out. At the time of this SNP selection (dbSNP build 126), allele frequencies were unknown for many of these SNPs, and SNPs with an unknown allele frequency were included.

### Prediction of MiHA epitope candidates

To predict potential MiHA epitopes encoded by this set of SNPs, we generated peptide sequences in silico, based on the nucleotide sequences of both allelic variants of the SNP. Peptide sequences were generated both from the canonical and from the two alternative forward reading frames, using gene segment encoding ten amino acids N-terminal and C-terminal of the SNP-containing codon. This peptide sequence set was then used to predict 9-, 10- and 11-mer polymorphic HLA-A2 binding peptides using three different HLA-peptide binding algorithms, Syfpeithi [25], Bimas [26] and netMHC [27]. Predicted HLA-A2 binding peptides were selected for further testing when at least passing the threshold for one of the three algorithms. Peptides predicted from an ARF were only selected if an upstream alternative start site was detected. In total, 973 unique peptides were selected with a predicted HLA-A2 binding affinity (Table S2). The successful prediction of known MiHA such as HMHA-1, indicated that the quality of the gene-expression and SNP databases combined with HLA-peptide binding algorithms was sufficient to predict putative MiHA (Figure S1g,h).

### Assessing the HLA-A2 binding affinity of predicted MiHA epitopes

**Figure 1:**
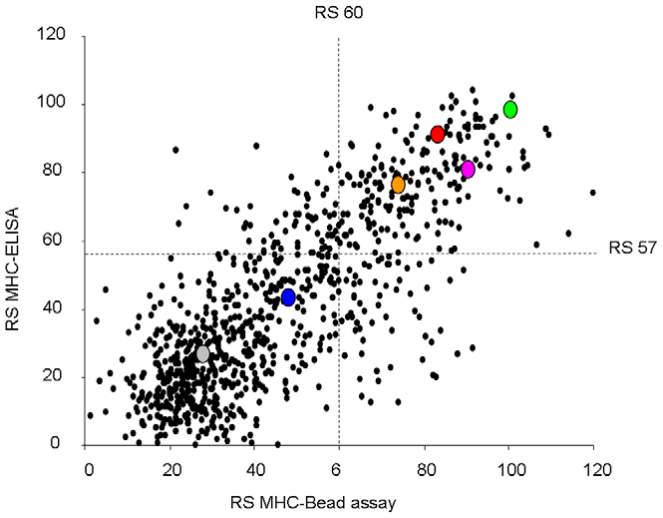
HLA-A2 affinity of predicted peptides measured in parallel by two different assays. HLA-A2 affinity of 973 predicted MiHA peptides was measured in parallel by two different binding assays. Each dot (black) represents a pMHC complex rescued by a tested peptide after UV induced cleavage of a conditional ligand. On the y-axis rescue score (RS) are shown for MHC-ELISA assay. On the x-axis RS are shown for MHC-bead assay. RS are normalized to the HLA-A2 high affinity CMV-pp65_NLV_ peptide and CMV-pp65_NLV_ peptide RS set to 100 for both assays. Selection threshold: RS≥57 (MHC-ELISA) and RS≥60 (MHC-bead assay). High affinity peptide controls: CMV-NLVPMVATV (green), FLU-GILGFVFTL (pink), EBV-GLCTLVAML (orange) and HA1-VLHDDLLEA (red). Low affinity peptide control: MART1-AAGIGILTV (blue) and negative control A3-gp100-LIYRRRLMK (grey).

To evaluate the HLA-A2 affinity of these predicted MiHA, the set of 973 peptides was synthesized and analyzed using two different MHC binding assays (“MHC ELISA” and “MHC bead assay”) that are both based on UV-induced conditional ligand cleavage, followed by peptide affinity dependent rescue of the MHC complex [18], [28], [29] (Figure 1). To set selection thresholds for both binding assays, a number of control peptides with high, intermediate or low HLA-A2 affinity were included. Peptide-MHC rescue scores (RS) were determined in both assays and normalized to the high affinity CMV-pp65_NLV_ peptide [30]. Results of both assays showed a clear correlation and all control peptides demonstrated the expected HLA-A2 affinity. Based on this analysis, 333 peptides with RS≥57 (MHC-ELISA) or RS≥60 (MHC-bead assay) were selected (Table S3).

### Analysis of efficiency and sensitivity for pull down of MiHA specific T-cell populations

Antigen specific T-cells can be present at very low frequencies. MiHA specific T-cell responses may therefore go undetected, especially when analyzed directly ex vivo in clinical specimens that often contain only a few million cells and that are generally not obtained during the peak GVL response. To allow high-throughput screening with a very large unbiased set of pMHC tetramers in PBMC samples with low cell numbers, we first developed an approach to simultaneously isolate T-cells reactive with any of the pMHC tetramers, and then expand these T-cells in vitro, prior to flow cytometric analysis. To address the sensitivity of this approach, we attempted the detection of a MiHA specific T-cell population in an allo-SCT patient PBMC sample obtained at 15 months after DLI, and in which HMHA-1 specific T-cells were barely detectable (∼0.01% of CD8^+^ T-cells) ex vivo. After magnetic pull down with the entire PE-labeled 333 pMHC tetramer set and subsequent expansion of the cells, HMHA-1 reactive T-cells were clearly detectable at a frequency of 2.56% of total CD8^+^ T-cells (Figure S2). Thus, magnetic pull down with large collections of pMHC tetramers can be used to facilitate detection of low-level T-cell responses.

### Identification of MiHA specific T-cell populations

Having successfully established the feasibility of our pull down and in vitro expansion method for the detection of MiHA specific T-cells in small PBMC samples, we subsequently utilized the entire set of 333 PE-labeled pMHC tetramers to pull down MiHA specific T-cells from 20 HLA-A2 positive allo-SCT patients with various hematologic malignancies. Selected patients all demonstrated a clear graft versus leukemia response after DLI, and samples were obtained at the memory phase of the GVL response, when MiHA specific T-cells are expected to be present but at low frequencies. Following magnetic isolation, isolated cells were expanded in vitro until cell numbers allowed the detection of MiHA-reactive T-cell populations by MHC tetramer combinatorial encoding [19], [20], [28], [31]. For this purpose, a set of fluorescently labeled pMHC tetramers was generated in which each specific pMHC complex was encoded by a unique combination of fluorochromes [20], to screen for recognition of all 333 selected epitopes in a limited number of stainings. The total set of selected epitopes was hierarchically clustered to 16 groups of up to 25 unique pMHC complexes according to the order of priority, i.e. SNP frequencies and HLA-A2 affinity (Table S3).

**Figure 2:**
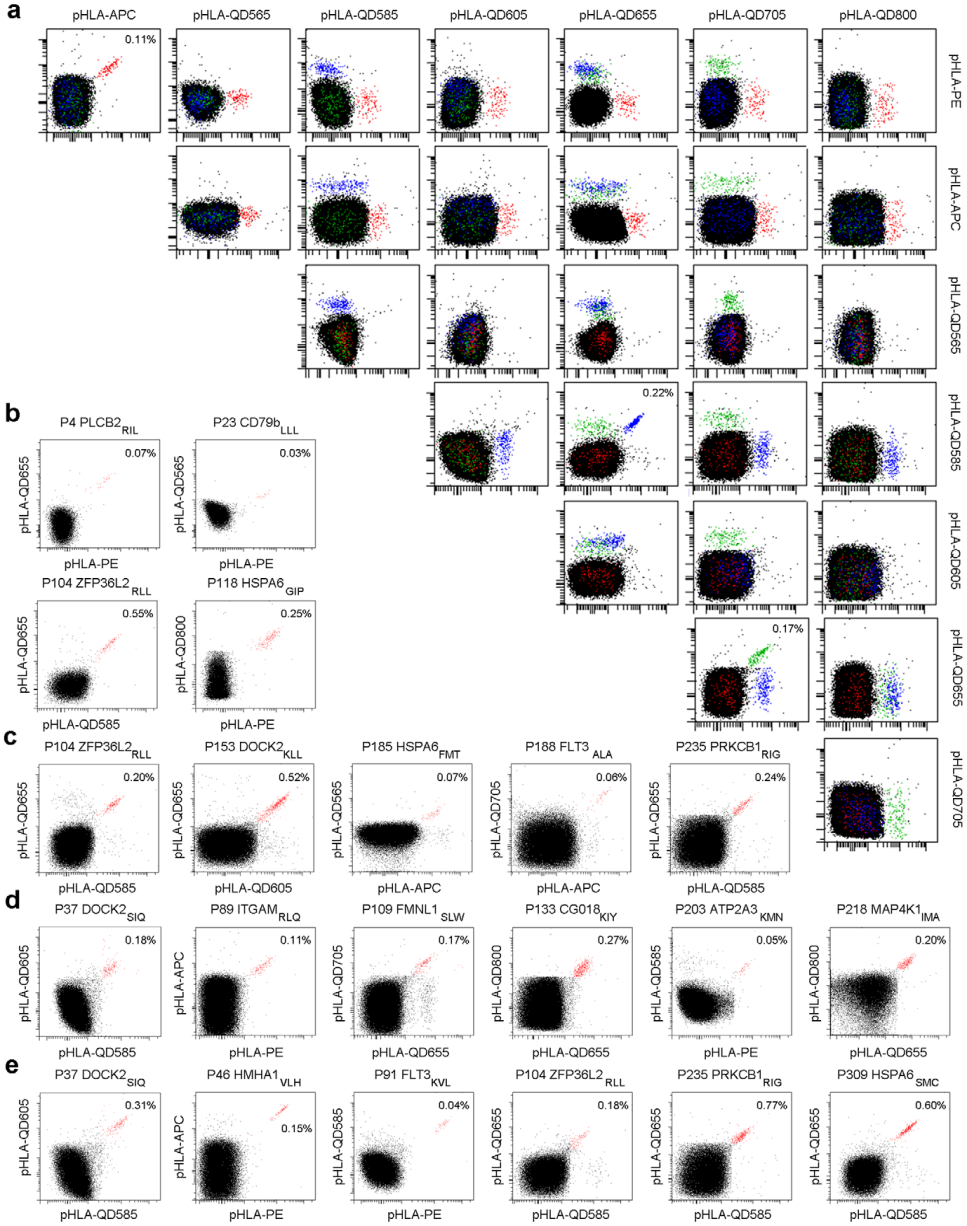
Detection of potential MiHA specific T-cell populations by pMHC tetramer staining. These FACS analyses show the detection of MiHA specific T-cell populations through dual-encoding after pMHC tetramer pull down and in vitro expansion. Shown are total CD8^+^ T-cells. All dot plots are shown with bi-exponential axes and display fluorescence intensity for the indicated fluorochromes at the top and right of the plot matrix. Non-pMHC tetramer specific CD8^+^ T-cells are indicated black. Dot plots of pMHC tetramer positive T-cell populations are shown by staining one expanded cell culture with 16 separate panels of up to 25 different dual-color pMHC tetramers. (**a**) Representative example of pMHC multimer screen panel 5, patient BDY3356. Detection of three dual-labeled potential MiHA specific T-cell populations: P89 ITGAM_RLQ_ (red), P104 ZFP36L2_RLL_ (blue) and P109 FMNL1_SLW_ (green). Frequencies indicate MiHA specific T-cells of total CD8^+^ cells. A selection of 21 potential MiHA specific T-cell populations was made with the highest clinical potential. Selected T-cell populations were derived from allo-SCT patient: OBB1465 (**b**), JMO2750 (**c**), BDY3356 (**d**) and APM4461 (**e**). Dot plots shown are representative for all detected dual-positive CD8^+^ T-cell populations (red).

After pull down and an average of two weeks of expansion, flow cytometric analysis of these samples revealed 71 different pMHC tetramer-reactive T-cell populations, specific for 47 unique pMHC complexes (Table S4). In most cases, T-cell frequencies varied between 0.02% and 4.9% of total CD8^+^ T-cells. A representative example of a full panel with 25 different 2-color coded pMHC complexes is shown in Fig. 2a, in which 3 potential MiHA-tetramer reactive T-cell populations were observed for the predicted MiHA peptides 89 (0.11%),104 (0.22%) and 109 (0.17%). In one patient we detected a T-cell population specific for the previously identified HMHA-1H epitope.

To assess the peptide specificity and functional activity of these T-cell populations we selected the 21 most interesting MiHA specific T-cell populations for the generation of cell lines by pMHC tetramer based cell sorting (Figure 2b–e). We based our selection on favorable SNP allele frequencies according to the dbSNP database and focused on T-cell populations that only showed reactivity with one of the allelic counterparts of a specific peptide. The purity of cell lines generated in this manner was verified by pMHC tetramer staining and 2 representative examples are shown in Figure S2c–d. We were able to generate cell lines with sufficient purity for subsequent functional assessment for 17 out of 21 selected T-cell populations.

### Assessing the functionality of isolated T-cell lines by peptide stimulation

**Figure 3:**
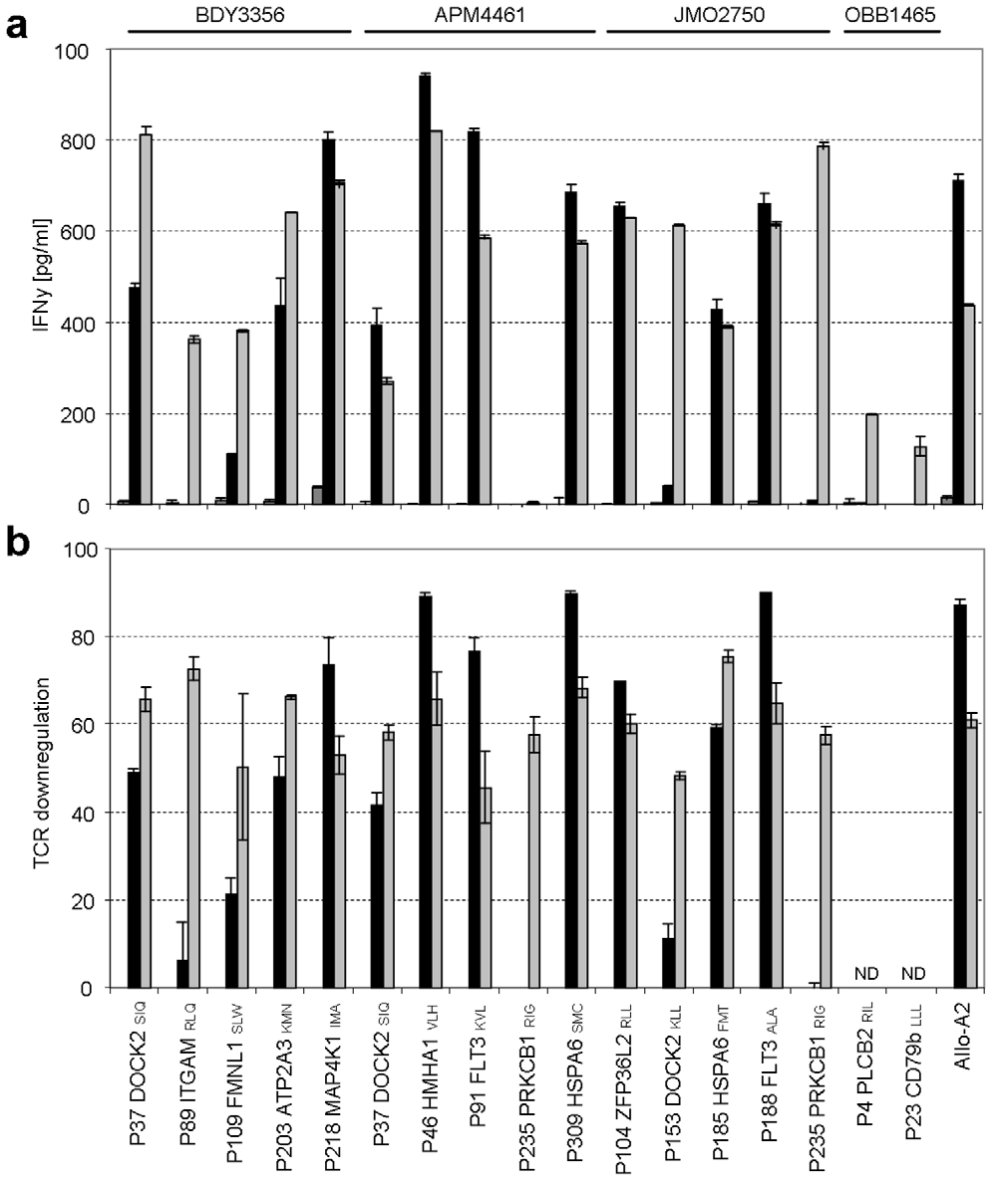
Peptide stimulation leads to IFN γ production and TCR downregulation for 10 out of 17 pMHC tetramer positive cell lines. Isolated pMHC tetramer positive cell lines were stimulated with peptide-loaded HLA-A2^+^ T2 target cells for 18 hours. Data is shown for 17 cell lines that were successfully generated by flowcytometry based cell sorting. Tested cell lines were derived from four different allo-SCT patients as indicated at the top of the graph. As a control an alloreactive CTL clone specific for a HLA-A2 epitope was used (Allo-A2). (**a**) Antigen specificity and functionality was analyzed by cytokine secretion in a standard IFN γ ELISA. Cell lines were stimulated with non-peptide loaded T2 cells (dark grey), [1 ug/ml] peptide-loaded T2 cells (black) and αCD3/CD28 stimulation beads (light grey). Data are presented as cytokine concentration. (**b**) Antigen specificity and functionality was analyzed by TCR internalization upon peptide stimulation. Cell lines shown were stimulated with [1 ug/ml] peptide-loaded T2 cells (black) and αCD3/CD28 stimulation beads (light grey). TCR downregulation was normalized to stimulation with non-peptide loaded T2 cell controls. Experiments were performed in duplicate, data are mean ± SD.

To analyze the functional activity of the isolated pMHC tetramer specific T-cells, we measured IFN γ production upon incubation with peptide-loaded HLA-A2^+^ target cells. As our T-cell isolations were solely based on pMHC tetramer reactivity and not on functional activity, we also measured the overall IFN γ secretion capacity of these cells, by nonspecific stimulation with αCD3/CD28 stimulation beads (Figure 3a). Although IFN γ production capacity varied, peptide specific IFN γ production could be demonstrated for 10 out of 17 pMHC tetramer positive cell lines, including 9 cell lines directed against potential MiHA and one directed against the known HMHA-1 epitope. Six cultures only demonstrated IFN γ production when stimulated with stimulation beads, indicating that these cell lines were not functionally reactive to peptide antigen. In addition, pMHC-tetramer positive cell line P235 PRKCB1_RIG_ totally lacked IFN γ production capacity.

Peptide-specificity of the 10 T-cell lines that produced IFN-γ upon peptide stimulation was confirmed by assessing TCR internalization upon stimulation with peptide-loaded target cells (Figure 3b). TCR downregulation clearly correlated with IFN γ production and was observed for all cell lines that demonstrated peptide specific IFN γ production. Minimal downregulation was observed for the four tested cell lines that lacked peptide specific IFN γ production, as well as the cell line that showed no overall IFN γ producing capacity. Hence, these data indicate that 10 out of 17 tested cell lines are reactive against their specific peptide when added exogenously.

### Wide range of peptide affinity observed for pMHC tetramer positive T-cell populations

**Figure 4:**
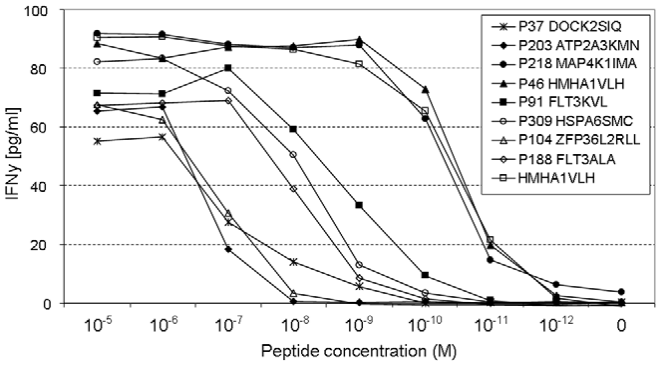
Analysis of peptide affinity of pMHC tetramer positive cell lines MHC tetramer positive T-cell lines demonstrated a wide range of peptide sensitivity. HLA A2^+^ T2 cells were pulsed with specific MiHA peptide. Peptide concentrations were titrated in 10-fold dilution steps starting from 10ug/ml. T-cell reactivity was analyzed by cytokine secretion in a standard IFN γ ELISA. Data are presented as cytokine concentration. Shown are eight representative generated T-cell lines and a high affinity control clone specific for HMHA-1H (open square). Cell lines APM4461 derived P37 DOCK2_SIQ_ and JMO2750 P185 HSPA6_FMT_ were not tested due to technical limitations.

To examine the ligand sensitivity of the generated peptide specific cell lines, INF-γ production was measured after stimulation with T2 cells that were loaded with a range of peptide concentrations (Figure 4). In this assay (performed for 8 representative cell lines), peptide concentrations required for T-cell recognition were compared to those required for a previously identified T-cell clone that is specific for the HMHA-1H epitope. This T-cell clone has been demonstrated to be present in a GVL response and was obtained during the subsequent memory phase. The 8 cell lines tested showed a wide range of peptide sensitivity. Specifically, the cell lines specific for P218 MAP4K1_IMA_ and P46 HMHA1_VLH_ were capable of target recognition at low picomolar peptide concentrations, similar to the peptide concentration required for the HMHA-1-specific control T-cell clone. Cell lines specific for P91 FLT3_KVL_, P309 HSPA6_SMC_ and P188 FLT3_ALA_ required low nanomolar peptide concentrations and those specific for P37 DOCK2_SIQ_, P203 ATP2A3_KMN_ and P104 ZFP36L2_RLL_ only showed target recognition at high nanomolar peptide concentrations (IC50: ±50 pM, ±5 nM and ±500 nM respectively for the three groups of cell lines). Comparison of the peptide concentration required for T-cell activation and the MHC binding affinity of the different peptides indicated that a requirement for high peptide concentrations to obtain T-cell activation was not simply due to a lower pMHC affinity. As an example, the peptides recognized by cell-lines P91 FLT3_KVL_ and P203 ATP2A3_KMN_ displayed a comparably high MHC affinity (as measured in Fig. 1) as those of the two highly sensitive cell lines P218 MAP4K1_IMA_ and P46 HMHA1_VLH_. Thus, the low peptide sensitivity of many of the isolated T-cell lines formed a direct reflection of a low affinity TCR-pMHC interaction.

### Isolated T-cell populations are not involved in the clinical response

**Figure 5:**
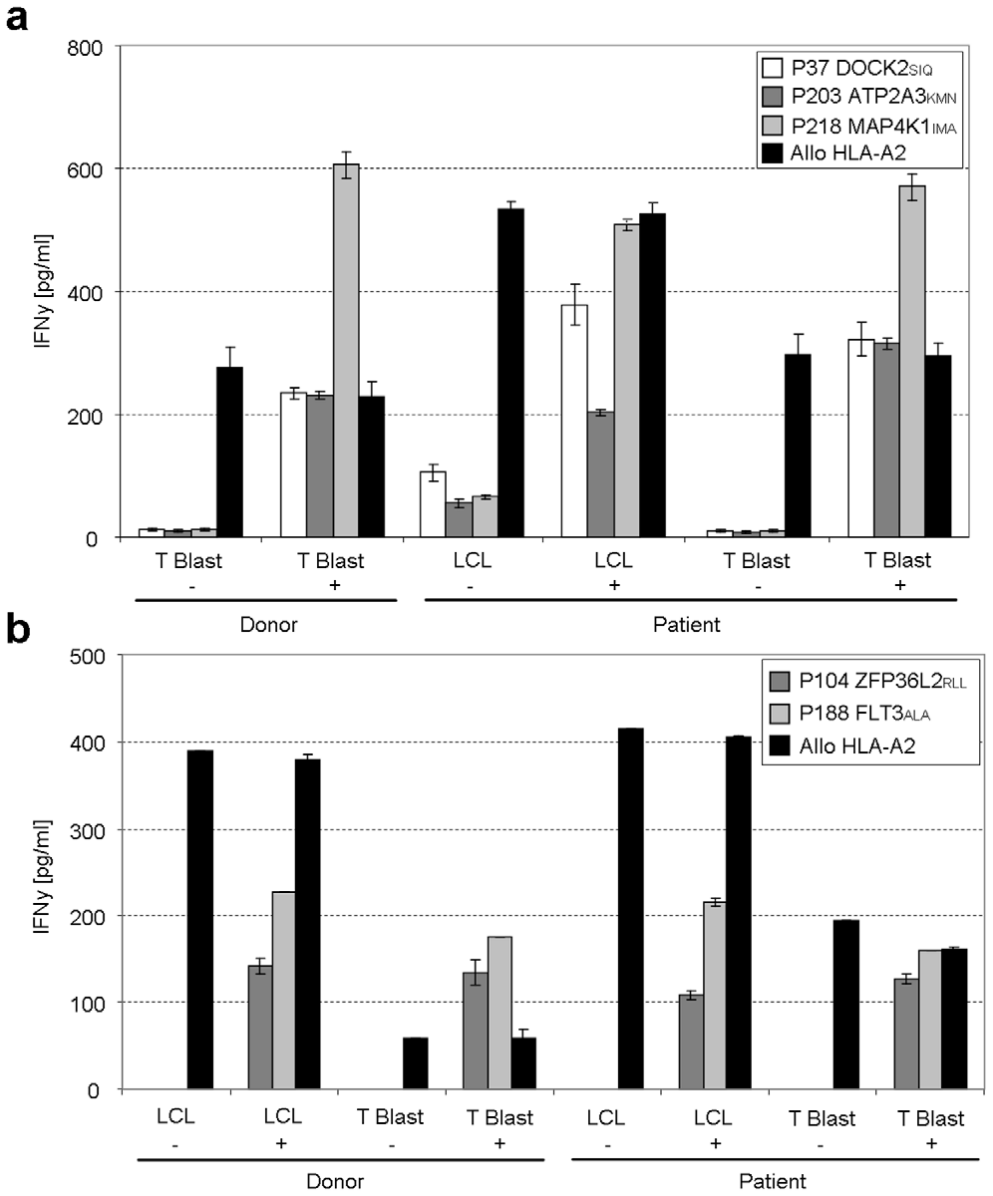
No recognition of hematopoietic donor and recipient target cells by MiHA specific T-cells. Isolated IFN γ producing cell lines were stimulated with HLA-A2^+^ donor and patient derived hematopoietic target cells for 18 hours. T-cell reactivity was measured in a standard IFN γ ELISA. Data are presented as cytokine concentration. Cell lines shown are representative for all cell lines. As a control for T-cell reactivity an alloreactive HLA-A2 specific CTL clone was used (black). (**a**) BDY3356 derived cell lines P37 DOCK2_SIQ_ (white), P203 ATP2A3_KMN_ (dark grey) and P218 MAP4K1_IMA_ (light grey) stimulation with donor and recipient T-cell blasts and EBV-LCLs loaded with (+) or without (−) specific peptide [1 ug/ml]. (**b**) JMO2750 derived cell lines P104 ZFP36L2_RLL_ (dark grey) and P188 FLT3_ALA_ (light grey) stimulation with donor and recipient T-cell and EBV blasts loaded with (+) or without (−) specific peptide [1 ug/ml].

To determine whether the observed T-cell reactivities could be involved in the clinical response observed after DLI in these patients, we screened the high- and intermediate-avidity T-cell lines for differential recognition of patient- and donor-derived EBV-LCLs and T-cell-blasts. Results are shown for 5 representative T-cell lines isolated from patient BDY3356 and JMO2750 (figure 5a,b). Peptide loaded target cells of both donor and recipient origin were recognized by all cell lines. In contrast, all cell lines were unable to recognize recipient target cells, indicating that these cells were not likely to be involved in the GVL response observed in these patients. As a control, all hematopoietic target cells were recognized by an HLA-A2 alloreactive CTL control clone, indicating that HLA-A2 expression was sufficient to allow target-cell recognition. Notably, recognition of recipient cells was also not observed for the HMHA-1 specific cell-line.

To test whether the lack of reactivity that was observed could be explained by absence of the immunogenic MiHA allele variants, we next screened the SNP haplotypes for selected donor-recipient pairs (Table 1). For 15 out of the 17 functionally tested cell lines, no SNP haplotype disparities were revealed for the potential MiHA concerned. In addition, only 7 out of 15 tested cell lines recognized “non-self” antigen variants (i.e. the variant not encoded by the donor genome), whereas 8 cell lines recognized “self” antigen variants. Interestingly, both high-avidity T-cell populations that were isolated (specific for P218 MAP4K1_IMA_ and P46 HMHA1_VLH_) recognized “non-self” antigen variants, whereas all cell lines that recognized “self” antigen variants were of intermediate or low-avidity. These results suggest a possible role for clonal deletion of high-avidity T-cells specific for these “self” epitopes when they are appropriately processed. Based on these results, we hypothesized that the low or intermediate avidity of most MiHA specific cell lines that we generated could explain the inability of these cell lines to recognize endogenously processed antigen. The only two cell lines that were derived from a transplantation setting in which there was a relevant SNP mismatch between the donor and recipient were specific for P23 CD79b_LLL_ and P109 FMNL1_SLW_. Although these cell lines could theoretically recognize the immunogenic MiHA allele variant of the patient, both cell lines demonstrated no recognition of endogenously processed antigen in the prior functional analyses.

**Table 1:** MiHA haplotype disparities in selected donor and recipient pairs.

Clone	Gene	Epitopeα	Epitope allele variant	Donor allele	Patient allele	Disparityβ	Clonal nature
P4	PLCB2	R I *L* V G R L R A A	A	AG	AA	no	Self
P23	CD79b	L L L *S* A E V Q Q H L	A	GG	AG	yes	non-self
P37 (BDY)	DOCK2	S *I* Q N Y H P F A	A	AA	AA	no	Self
P37 (APM	DOCK2	S *I* Q N Y H P F A	A	AA	AA	no	self
P46	HMHA1	V L *H* D D L L E A	A	GG	GG	no	non-self
P89	ITGAM	*R* L Q V P V E A V	G	GG	GG	no	self
P91	FLT3	K V L H E L F G *M* D I	A	AA	AA	no	self
P104	ZFP36L2	*R* L L P L W A A L P L	G	GG	GG	no	self
P109	FMNL1	S L W Q L *G* A A V M L	G	CC	CG	yes	non-self
P153	DOCK2	K L L Q I Q L *R* A	G	GG	GG	no	self
P203	ATP2A3	K M N V F D T *N* L	A	GG	GG	no	non-self
P218	MAP4K1	I *M* A I E L A E L	A	GG	GG	no	non-self
P235 (APM)	PRKCB1	R I G Q R Q *E* T V	G	AA	AA	no	non-self
P235 (JMO)	PRKCB1	R I G Q R Q *E* T V	G	AA	AA	no	non-self
P309	HSPA6	S M C R F S P L T *L*	A	AG	AG	no	self

α Polymorphic residue in italic.

β Disparities are indicated in respect to the donor haplotype.

### Assessing the MiHA recognition potential of isolated T-cell populations

The above data indicate that unbiased MHC tetramer-based enrichment often results in the isolation of T-cell populations that do not play a role in GVL, as based on the lack of the relevant mismatch. However, this does not exclude that such cell populations could recognize target cells that do express the relevant MiHA allele. To investigate the potential capacity of these MiHA specific T-cell populations to recognize MiHA allele -positive target cells, the cell lines were tested against a panel of SNP-genotyped HLA-A2^+^ EBV-LCLs. Interestingly, the HMHA-1 specific T-cell population, isolated from a transplantation setting in which both donor and recipient were homozygous negative for the immunogenic allele variant of the MiHA, recognized target cells in accordance with their SNP haplotype. Specifically, this tested cell line strongly recognized non-peptide loaded homozygote positive and heterozygote EBV-LCLs, whereas homozygote negative EBV-LCLs were not recognized. In contrast, the second high avidity P218 MAP4K1_IMA_ specific cell-line, as well as the intermediate avidity cell lines were unable to recognize any target in the SNP-typed EBV-LCL panel.

### Lack of target cell recognition by high-avidity T-cells is caused by inappropriate processing and surface presentation of predicted epitope

**Figure 6:**
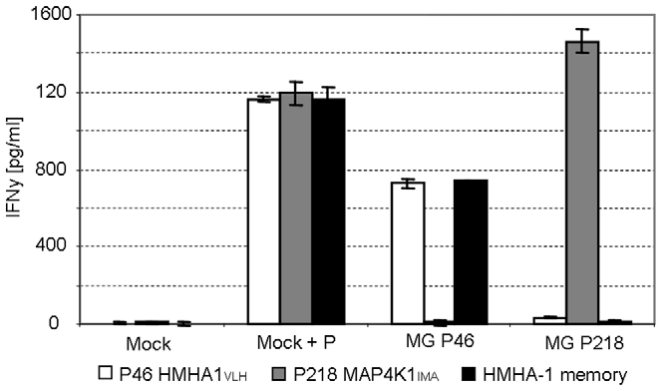
Recognition of EBV target cells by high-avidity MiHA T-cells after minigene transduction. High-avidity cell lines P46 HMHA1_VLH_ (white) and P218 MAP4K1_IMA_ (grey) were stimulated with HLA-A2^+^ EBV-LCL JY transduced with minigene constructs (MG) encoding minimal peptide sequence directly attached to an ER-signal sequence. T-cell reactivity was measured after 18 hours in a standard IFN γ ELISA. Data are presented as cytokine concentration. The MOCK transduced cells only encoded an ER-signal sequence. As a control for T-cell reactivity an alloreactive HLA-A2 specific CTL clone was used (black).

The P218 MAP4K1_IMA_ specific T-cell population demonstrated efficient recognition of target cells loaded with picomolar concentrations of peptide, whereas no reactivity was observed in accordance with the SNP haplotype of tested targets. To determine whether the inability of this high avidity T-cell line to recognize endogenously processed antigen was due to the inability of the target cells to process and present the MAP4K1_IMA_ epitope, a retroviral minigene vector was constructed that encoded the minimal MAP4K1_IMA_ peptide sequence directly attached to an ER-signal sequence [32]. In this design, delivery of the potential T-cell epitope to the ER occurs co-translationally, and hence independent of proteasomal processing and TAP transport. As a positive control, a similar minigene was created for the HMHA-1H epitope. HLA-A2^+^ JY cells that are homozygous negative for both the P218 MAP4K1_IMA_ and the HMHA-1H allele were transduced with the two minigene constructs, and demonstrated to be recognized by the HMHA-1 T-cell line as well as by the MAP4K1_IMA_ specific T-cell line (Figure 6). These results indicate that when endogenous presentation of the predicted MAP4K1_IMA_ epitope is forced, recognition by P218 MAP4K1_IMA_ specific T-cells is strong.

## Discussion

This study demonstrates the feasibility and limitations of using a reverse immunology approach for the identification of potential MiHA. We combined large scale prediction of HLA-restricted MiHA with functional assessments of these polymorphic epitopes by identifying MiHA specific T-cell populations in a high-throughput unbiased fashion. In this study we used a reverse immunology approach, based on UV-induced peptide exchange technology, in which the predicted MiHA epitopes were the starting point for identification of new MiHA specific T-cell responses. By investigating MiHA source proteins with a hematopoietic tissue restricted expression pattern, we aimed to identify potential CTL epitopes that would selectively target recipient hematopoiesis.

The combined use of three prediction programs resulted in the generation of a synthetic peptide library of 973 experimental peptides encoded by hematopoietic stem cells. The MHC binding capacity of these peptides was verified by UV-induced MHC-peptide exchange. Based on the binding capacity of well studied natural ligands, we estimate that one third of the predicted peptide set could be defined as high affinity, one third as intermediate affinity and one third as low affinity HLA-A2.

To assess the ability of these epitopes to serve as TCR ligands we monitored T-cell responses in a high throughput unbiased fashion using multiplexed fluorescently labeled sets of pMHC complexes. We were able to detect epitope specific T-cell recognition for 71 of the 333 screened pMHC complexes of which 24 epitopes were recognized in multiple and 47 epitopes were recognized in single individuals. Taken into account that these T-cells were isolated from small cell size PBMC samples of allo-SCT patients with only partially reconstituted TCR repertoires, this data highlights both the immense capacity of the TCR repertoire to recognize random HLA-ligands and the high-sensitivity of our enrichment protocol. Unfortunately, most isolated pMHC tetramer positive T-cells appeared to be of low or intermediate avidity. Two cell lines recognized their respective peptide with high avidity. In the case of MAP4K1_IMA_, no reactivity against endogenously processed antigen was observed, but cells expressing a minigene encoding this epitope were efficiently recognized, suggesting that inappropriate processing explains the lack of recognition of epitope derived from the parental protein. The second high avidity T-cell population recognized the HMHA-1H epitope, and target cell recognition by the T-cell line fully matched HMHA-1 status.

As the identification of the HMHA-1 and MAP4K1_IMA_ specific T-cell responses in our experiments occurred in a fully unbiased screen, this forms evidence that the type of genome-wide screen developed here can be successful. Nevertheless, the fact that only the HMHA-1 specific T-cell population showed recognition of endogenously produced antigen shows that this discovery process is still highly suboptimal, and we see 3 major areas for improvement for this.

First, the capacity of a specific T-cell to bind to a pMHC tetramer does not necessarily reflect its capacity to elicit potent T-cell reactivity when stimulated with a relevant pMHC complex. In this study only 2 out of the 16 T-cell populations that produced IFN γ upon nonspecific stimulation also demonstrated IFN γ production (and TCR internalization) at picomolar range peptide concentrations. Importantly, these high-avidity T-cell populations do not necessarily demonstrate a more intense pMHC tetramer staining intensity as compared to low and intermediate avidity T-cell populations, making it difficult to weed out less interesting T-cell populations on the basis of MHC tetramer staining intensity. Thus, alternative strategies are required to obtain a rough estimate of T-cell sensitivity early in the screening process.

Second, in this screen, T-cell populations were isolated using the full set of pMHC tetramer complexes for each sample, irrespective of SNP status of donor and recipient. The frequent encounter of low avidity pMHC tetramer positive T-cells from donors for which this epitope forms a “self” antigen could therefore reflect clonal deletion of high avidity T-cells, due to presentation of the predicted epitopes in the donor thymus. In future screens it seems useful to apply stringent epitope selection criteria to restrict high-throughput analysis to those epitopes that can be considered neo-antigens in a given transplant combination, something that can readily be done by evaluation of donor (and recipient) SNP status.

Third, of the two high avidity T-cell populations isolated, only one could recognize epitopes derived from the endogenous antigen. In this project ligand prediction focused solely on the HLA affinity of predicted peptides and disregarded other aspects of the HLA processing and presentation pathway. As a consequence, many of the predicted epitopes used here may not be part of the natural peptidome and thereby lack biological relevance. In future work, this issue may to some extent be addressed by the use of antigen processing algorithms that predict proteosomal cleavage and TAP-dependent peptide transport [33], [34]. As a second, and in our view even more attractive option, the peptide set used for high-throughput screening could be derived from a database of HLA eluted peptides, thereby guaranteeing presentation of the epitopes concerned.

In conclusion, our isolation and detailed analysis of potential MiHA candidates in a high-throughput fashion has revealed the technical feasibility of this reverse immunology approach. We have demonstrated that TCR repertoires against very large sets of antigens can rapidly be screened. However, the productive use of such high-throughput screening technology will require further improvements. In particular, stringent epitope selection criteria including the availability of high quality databases of MHC ligands and SNP genotypes are likely to be of value to increase the percentage of isolated T-cell populations that is not only pMHC tetramer reactive but also biologically relevant.

## Materials and Methods

### PBMC samples and T-cell staining

After study approval of the Leiden University Medical Center institutional review board, PBMC samples were obtained from allo-SCT patients during the memory phase of a graft versus leukemia response after DLI as determined by mixed hematopoietic chimerism and/or quantitative BCR-ABL analysis after approval of the Leiden University Medical Center institutional review board and informed consent according to the Declaration of Helsinki. Informed consent form all participants involved in this study were written for samples obtained since 2003 and verbal for older samples when guidelines provided no written consent. PBMC were isolated by Ficoll gradient centrifugation, and frozen in liquid nitrogen. For T-cell staining of approximately 1×10^6^ PBMC a final concentration of 2 µg/mL per pMHC tetramer was added and incubated for 15 min at 37°C. Next, antibody-mix consisting of CD8-Alexa700 (Caltag) and CD4-, CD14-, CD16-, CD19- and CD40-FITC (BD) was added and cells were incubated for 30 min at 4°C. Prior to flow cytometry, cells were washed twice and Propidium lodide (PI) was added to allow dead cell exclusion. Dual-encoding pMHC tetramer analysis was performed as previously described [20].

### Gene expression of hematopoietic cell fractions by microarray analysis

Hematopoietic precursor CD34^+^ cells were isolated by MACS (Miltenyi) from bone marrow; G-CSF mobilized peripheral blood and cord blood PBMC according to manufacturer's protocol. Total RNA was isolated using Trizol (Invitrogen) and transcribed into cDNA by reverse transcriptase (Invitrogen) using oligo-dT primers (Roche Diagnostics). Microarray analysis of gene expression profiles in CD34^+^/CD38^−^ and CD34^+^/CD38^+^ fractions was performed by Affymetrix U133 array according to the manufacturer's instructions. Additional gene expression information was retrieved from the NCBI Gene Expression Omnibus database [23].

### Prediction of HLA-A2^+^ MiHA ligands

The following prediction algorithms were applied to the peptide candidates: Syfpeithi [25], Bimas [26] and netMHC [27]. Peptides with a score of ≥19 (Syfpeithi), ≥1 (Bimas) and ≤875 (netMHC) were considered to have potential HLA-A2^+^ binding capacities. SNP data was retrieved from NCBI's dbSNP polymorphism database [24]. Amino acid sequences were obtained from NCBI Entrez engine.

### Generation of peptide-MHC complexes

All peptides were synthesized in-house using standard Fmoc chemisty or purchased from Pepscan (Pepscan Presto). The UV-sensitive building block J was synthesized as described [18]. Recombinant HLA-A2 heavy chain and human β_2_m light chain were in-house produced in Escherichia coli. MHC class I refolding was performed as previously described with minor modifications [20]. MHC class I complexes were purified by gel-filtration HPLC in PBS. Peptide-MHC complexes were generated by MHC-peptide exchange. Prefolded UV-liable pMHC complexes (100 µg/ml) were subjected to 366nm UV light (Camag) for 1 h in presence of the specific peptide (200 µM) [19], [28]. After exchange, samples were spun at 16,000 g for 5 min and supernatants were used for pMHC tetramer formation. The peptide HLA-A2^+^ binding affinity was assessed using two different HLA-binding assays in parallel; MHC-ELISA and MHC bead as previously described [18], [29]. For generation of pMHC tetramers, 8 different fluorochrome-streptavidin (SA) conjugates were used as previously described [20]. Phycoerythrin (PE), allophycocyanin (APC) and the quantum dots (QD); QD565, QD585, QD605, QD655, QD705 and QD800 were used (Invitrogen). Complexes were stored at 4°C and prior to use pMHC tetramers were spun at 17.000 g for 2 min.

### Isolation of MiHA specific T-cells by pMHC tetramer pull down

Prior to isolation of peptide-specific T-cells, pMHC tetramers were made coupled to SA-PE. PBMC were stained with pMHC tetramers for 1 hour at 4°C. Subsequently, cells were washed and incubated with anti-PE Ab coated magnetic beads (Miltenyi). Cells were than isolated by MACS (Miltenyi), using an LS column, following the manufacturers protocol. Eluted cells were washed and cultured in Iscove Modified Dulbecco Medium (IMDM; Lonza BioWhittaker) supplemented with 5% human serum, 5% fetal calf serum (FCS; Cambrex), 100 IU/mL IL-2 (Chiron), 10ng/mL IL-15 (Peprotech). Eluted cells were cultured per 5000 cells with 2×10^4^ irradiated autologous feeder cells and 5000 anti-CD3/CD28 Dynabeads (Invitrogen) in 96-well plates. Cultures were split at least twice a week. After 2–3 weeks, cell cultures were analyzed for peptide-specific T-cell populations by pMHC tetramer flow cytometry. Subsequently pMHC tetramer reactive T-cell populations were sorted on a FACSAria (Becton Dickinson) into 96 well plates containing 1×10^5^ irradiated feeder cells supplemented with 0.5 µg/mL phytohaemagglutinin (PHA; Biochrom AG) as previously described [35].

### Flow cytometry

Data acquisition was performed on an LSR-II flow cytometer (Becton Dickinson) with FacsDiva software using the following 11-color instrument settings: 488nm laser: PI: 685LP, 695/40; PE: 550LP, 575/26; FITC: 505LP, 530/30; SSC: 488/10. 633nm laser: Alexa700: 685LP, 730/45; APC: 660/20. 405nm laser: QD800: 770LP, 800/30; QD705: 680LP, 710/50; QD655: 635LP, 660/40; QD605: 595LP, 650/12. 355nm laser: QD585: 575LP, 585/15; QD565: 545LP: 560/20. Approximately 200,000 lymphocytes were recorded for each analysis. To identify antigen-specific T-cells we followed the gating strategy as described in Figure S2.

### IFN γ release assay

MiHA specific T-cell lines (1×10^4^) were stimulated with HLA-A2^+^ T2 cells, EBV-LCLs or T-cell blasts (2,5×10^4^) in 96 well plates for 18 h at 37°C and 5% CO_2_. Peptide pulsing was performed by incubating stimulator cells for 1 h with synthetic peptides (1 µg/ml) in IMDM containing 2% FCS and cells were washed twice before use. Cytokine release was measured by IFN γ ELISA (Sanquin) according to the manufacturer's instructions.

## Supporting Information

Figure S1 **Gene expression profiles and MiHA prediction of hematopoiesis-restricted genes obtained with microarray analysis.** Gene expression profiles of CD34^+^/CD38^−^ and CD34^+^/CD38^+^ hematopoietic precursor cell populations. On the x-axis 100 different tissues or cell material are shown, clustered by organ system. On the y-axis the mRNA expression of the gene is shown. SC: stem cells, BM: bone marrow, PB: peripheral blood, Imm: immunological tissues, Apc: antigen presenting cells, Mal: hematological malignancies, CNS: central nervous system. Repr: reproductive organs, Gla: endocrine glands: Conn: connective tissues, Li: liver, Lu: lung, Dig: digestive tracts, Hea: heart, SK: skin, EC: endothelial cells. (**a,b**) HMHA-1 and PTPRC (CD45) have a clear hematopoiesis-restricted gene expression pattern. (**c**) Prostate kallikrein 2 (KLK2) demonstrate a tissue-specific gene expression pattern. (**d**) glyceraldehyde-3-phosphate dehydrogenase (GAPD) is ubiquitously expressed. (**e,f**) Integrin beta 2 (ITGB2) and FMS-like tyrosine kinase 3 (FLT3) were identified as genes (2 out of 78) with a hematopoiesis-restriced gene expression pattern by data-mining the combined microarray database. MiHA prediction based on peptide sequences representing the nucleotide sequences of both allelic variants of a SNP using three HLA-peptide binding algorithms. Polymorphic residue encoding triplet is highlighted (grey) and start-codon is underlined. ARF: alternative reading frame, NRF: normal reading frame S: Syfpeithi [25], B: Bimas [26], N: netMHC [27]. (*): Only N was used to predict 11-mers HLA-binding. Selection threshold S: binding score (BS) ≥19, B: BS≥1, N: BS≤500. (**g**) Prediction of MHMA-1 epitopes for RH139 polymorphism. Described immunogenic MiHA epitope; MHMA-1H and allelic variant are highlighted (red). (**h**) Prediction of ITGB2 epitopes for KE630 polymorphism.
(PDF)

Figure S2 **Flowcytometric analysis of pMHC tetramer specific cell lines.** **Flowcytometric analysis of HMHA-1 specific T-cells in an allo-SCT patient sample obtained 15 months after DLI, during the memory phase of the GVL response**. (**a**) Life gating strategy to reduce background pMHC tetramer staining. FSC and SSC width and height channels were used to reduce background staining. Propidium iodide (PI) was used as a death cell marker. In all plots total lymphocytes are grey, total CD8^+^ T-cells are black and pMHC tetramer positive T-cells are highlighted red. Dot plots are shown with bi-exponential axes and display fluorescence intensity for the indicated fluorochromes. (**b**) Flowcytometric analysis of HMHA-1 specific T-cells after pull down and in vitro expansion. pMHC tetramer positive T-cell frequencies are expressed as total CD8^+^ T-cells. (**c,d**) Flowcytometric purity analysis of pMHC tetramer specific cell lines. Dot plots show total lymphocytes (black). Dot plots are shown with bi-exponential axes and display fluorescence intensity for the specific pMHC tetramer complexes on the x-axis and CD8 expression on the Y-axis. Shown frequencies indicate pMHC tetramer positive T-cells out of total lymphocytes. (**c**) BDY3356 derived CD8^+^ cell line: P37 DOCK2_SIQ_. (**d**) APM4461 derived CD8^+^ cell line: P46 HMHA1_VLH_. Dot plots shown are representative for all generated cell lines.
(PDF)

Table S1 **Identified genes with a hematopoiesis-resricted expression pattern.**
(PDF)

Table S2 **Total MiHA epitopes predicted by HLA-peptide binding algorithm.**
(PDF)

Table S3 **Dual-encoding pMHC tetramer scheme.**
(PDF)

Table S4 **Total pMHC tetramer-reactive T-cell populations revealed after pull down and two weeks of expansion.**
(PDF)
