# Supplementary material for: High-Throughput Identification of Potential Minor Histocompatibility Antigens by MHC Tetramer-Based Screening: Feasibility and Limitations
Source: PLoS One. 2011 Aug 5;6(8):e22523. doi: 10.1371/journal.pone.0022523 (PMC3151248; doi:10.1371/journal.pone.0022523)
Supplement: Figure S1 — Gene expression profiles and MiHA prediction of hematopoiesis-restricted genes obtained with microarray analysis. Gene expression profiles of CD34+/CD38− and CD34+/CD38+ hematopoietic precursor cell populations. On the x-axis 100 different tissues or cell material are shown, clustered by organ system. On the y-axis the mRNA expression of the gene is shown. SC: stem cells, BM: bone marrow, PB: peripheral blood, Imm: immunological tissues, Apc: antigen presenting cells, Mal: hematological malignancies, CNS: central nervous system. Repr: reproductive organs, Gla: endocrine glands: Conn: connective tissues, Li: liver, Lu: lung, Dig: digestive tracts, Hea: heart, SK: skin, EC: endothelial cells. (a,b) HMHA-1 and PTPRC (CD45) have a clear hematopoiesis-restricted gene expression pattern. (c) Prostate kallikrein 2 (KLK2) demonstrate a tissue-specific gene expression pattern. (d) glyceraldehyde-3-phosphate dehydrogenase (GAPD) is ubiquitously expressed. (e,f) Integrin beta 2 (ITGB2) and FMS-like tyrosine kinase 3 (FLT3) were identified as genes (2 out of 78) with a hematopoiesis-restriced gene expression pattern by data-mining the combined microarray database. MiHA prediction based on peptide sequences representing the nucleotide sequences of both allelic variants of a SNP using three HLA-peptide binding algorithms. Polymorphic residue encoding triplet is highlighted (grey) and start-codon is underlined. ARF: alternative reading frame, NRF: normal reading frame S: Syfpeithi [25], B: Bimas [26], N: netMHC [27]. (*): Only N was used to predict 11-mers HLA-binding. Selection threshold S: binding score (BS) ≥19, B: BS≥1, N: BS≤500. (g) Prediction of MHMA-1 epitopes for RH139 polymorphism. Described immunogenic MiHA epitope; MHMA-1H and allelic variant are highlighted (red). (h) Prediction of ITGB2 epitopes for KE630 polymorphism. (PDF) [file pone.0022523.s001.pdf]

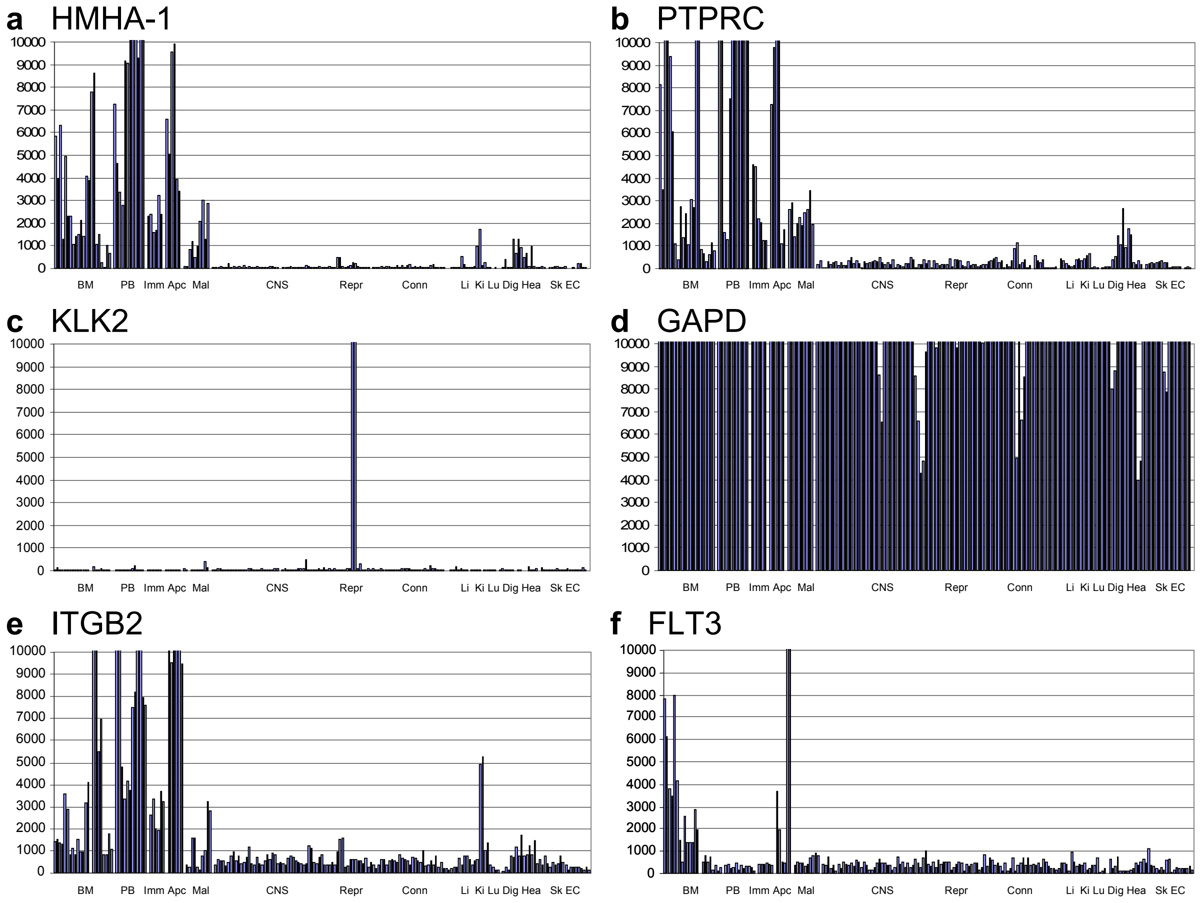

g Prediction of HMHA-1 epitopes

Gene: HMHA1 RefSeq mRNA seq: NM_012292.2
SNP: RH139 dbSNP ID: rs_1801284

ARF G P * E T * G V C V A * R P P * G P P P A
NRF G L E K L K E C V L **R** D D L L E A R R P R
ARF A L R N L R S V C C V T T S L R P A A R G
5' gagggccttgagaaacttaaggagtgtgtgttg**cgt**gacgacctccttgaggcccgccgcccgcgggcc 3'

5' gagggccttgagaaacttaaggagtgtgtgttg**cat**gacgacctccttgaggcccgccgcccgcgggcc 3'
ARF A L R N L R S V C C M T T S L R P A A R G
NRF G L E K L K E C V L **H** D D L L E A R R P R
ARF G P * E T * G V C V A * R P P * G P P P A

HMHA1	S	B	N
V L **R** D D L L E A	23	6	71
V L **H** D D L L E A	23	80	15
S V C C **V** T T S L	20	2	1613
S V C C **M** T T S L	20	2	1450

h Prediction of ITGB2 epitopes

Gene: ITGB2 RefSeq mRNA seq: NM_000211.3
SNP: KE630 dbSNP ID: rs_2230531

ARF L T L W Q V H L L R R V P E V R K G P L W
NRF S P C G K Y I S C A **E** C L K F E K G P F G
ARF H P V A S T S P A P S A * S S K R A P L G
5' ccctcaccctgtggcaagtacatctcctgcgcc**gag**tgcctgaagttcgaaaagggcccctttgggaag 3'

5' ccctcaccctgtggcaagtacatctcctgcgcc**aag**tgcctgaagttcgaaaagggcccctttgggaag 3'
ARF H P V A S T S P A P S A * S S K R A P L G
NRF S P C G K Y I S C A **K** C L K F E K G P F G
ARF L T L W Q V H L L R Q V P E V R K G P L W

ITGB2	S	B	N
H L L R **R** V P E V	29	20	29
H L L R **Q** V P E V	29	20	31
V H L L R **R** V P E V	20		
V H L L R **Q** V P E V	20		
W Q V H L L R **R** V		15	
W Q V H L L R **Q** V		15	
H L L R **Q** V P E V R K	*	*	
T L W Q V H L L R **R** V	*	*	70
T L W Q V H L L R **Q** V	*	*	70

Supplementary Figure 1
