# Supplementary figures and images for: High-Throughput Identification of Potential Minor Histocompatibility Antigens by MHC Tetramer-Based Screening: Feasibility and Limitations
Source: PLoS One. 2011 Aug 5;6(8):e22523. doi: 10.1371/journal.pone.0022523 (PMC3151248; doi:10.1371/journal.pone.0022523)

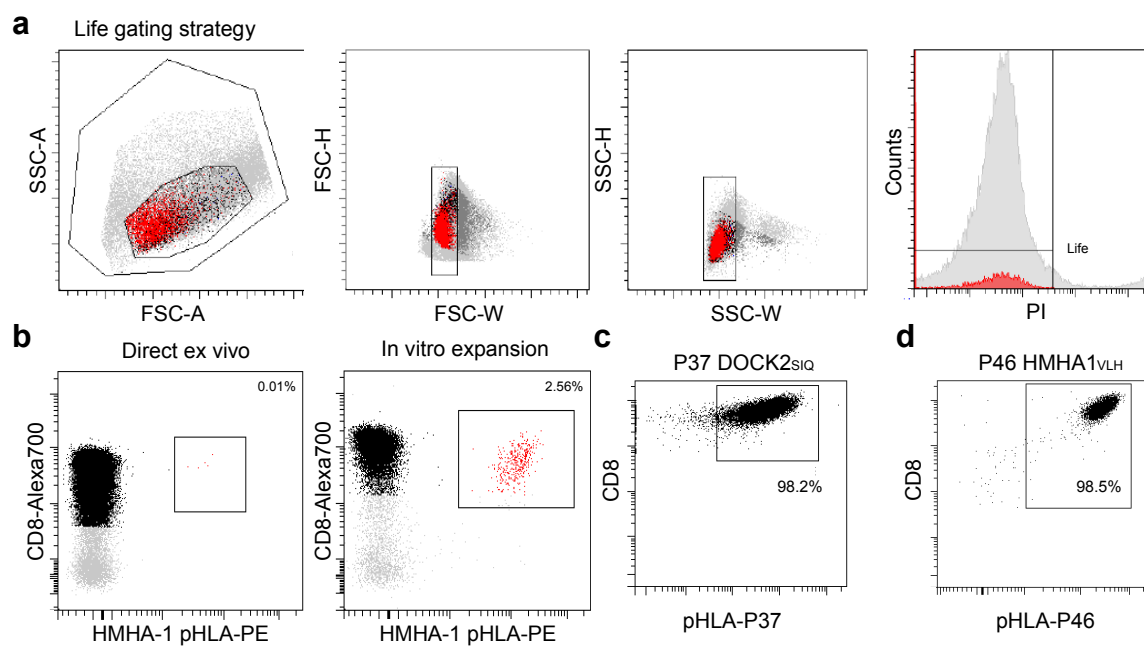

Supplementary Figure 2

Supplement: Figure S2 — Flowcytometric analysis of pMHC tetramer specific cell lines. Flowcytometric analysis of HMHA-1 specific T-cells in an allo-SCT patient sample obtained 15 months after DLI, during the memory phase of the GVL response. (a) Life gating strategy to reduce background pMHC tetramer staining. FSC and SSC width and height channels were used to reduce background staining. Propidium iodide (PI) was used as a death cell marker. In all plots total lymphocytes are grey, total CD8+ T-cells are black and pMHC tetramer positive T-cells are highlighted red. Dot plots are shown with bi-exponential axes and display fluorescence intensity for the indicated fluorochromes. (b) Flowcytometric analysis of HMHA-1 specific T-cells after pull down and in vitro expansion. pMHC tetramer positive T-cell frequencies are expressed as total CD8+ T-cells. (c,d) Flowcytometric purity analysis of pMHC tetramer specific cell lines. Dot plots show total lymphocytes (black). Dot plots are shown with bi-exponential axes and display fluorescence intensity for the specific pMHC tetramer complexes on the x-axis and CD8 expression on the Y-axis. Shown frequencies indicate pMHC tetramer positive T-cells out of total lymphocytes. (c) BDY3356 derived CD8+ cell line: P37 DOCK2SIQ. (d) APM4461 derived CD8+ cell line: P46 HMHA1VLH. Dot plots shown are representative for all generated cell lines. (PDF) [file pone.0022523.s002.pdf]
