# Supplementary material for: High-Throughput Identification of Potential Minor Histocompatibility Antigens by MHC Tetramer-Based Screening: Feasibility and Limitations
Source: PLoS One. 2011 Aug 5;6(8):e22523. doi: 10.1371/journal.pone.0022523 (PMC3151248; doi:10.1371/journal.pone.0022523)
Supplement: Table S1 — Identified genes with a hematopoiesis-resricted expression pattern. (PDF) [file pone.0022523.s003.pdf]

Supplementary Table I. Identified genes with a hematopoiesis-resricted expression pattern

Gene symbol	Official full name	mRNA accession	Protein accession
AIF1	Allograft inflammatory factor 1	NM_001623.3	NP_001614.3
AREG	Amphiregulin	NM_001657.2	NP_001648.1
ARHGAP4	Rho GTPase activating protein 4	NM_001164741.1	NP_001158213.1
ARHGAP15	Rho GTPase activating protein 15	NM_018460.3	NP_060930.3
ARHGAP25	Rho GTPase activating protein 25	NM_001007231.1	NP_001007232.1
ATP2A3	Sarcoplasmic/endoplasmic reticulum calcium ATPase 3 isoform a	NM_005173.2	NP_005164.2
BTK	Bruton agammaglobulinemia tyrosine kinase	NM_000061.2	NP_000052.1
CBFA2T3	Protein CBFA2T3 isoform MTG16b	NM_005187.4	NP_005178.4
CCL3	Chemokine (C-C motif) ligand 3	NM_002983.2	NP_002974.1
CD37	CD37 molecule	NM_001774.2	NP_001765.1
CD48	CD48 molecule	NM_001778.2	NP_001769.2
CD52	CD52 molecule	NM_001803.2	NP_001794.2
CD69	CD69 molecule	NM_001781.2	NP_001772.1
CD79b	CD79b molecule, immunoglobulin-associated beta	NM_000626.2	NP_000617.1
CD83	CD83 molecule	NM_001040280.1	NP_001035370.1
CENTB1	ArfGAP with coiled-coil, ankyrin repeat and PH domains 1	NM_014716.3	NP_055531.1
CG018	NEDD4 binding protein 2-like 1	NM_001079691.1	NP_001073159.1
CORO1A	Coronin, actin binding protein, 1A	NM_001193333.2	NP_001180262.1
CPVL	Carboxypeptidase, vitellogenic-like	NM_019029.2	NP_061902.2
CRHBP	Corticotropin releasing hormone binding protein	NM_001882.3	NP_001873.2
CSF3R	Colony stimulating factor 3 receptor	NM_000760.3	NP_000751.1
CXorf9	SAM and SH3 domain containing 3	NM_018990.3	NP_061863.1
DOCK2	Dedicator of cytokinesis 2	NM_004946.2	NP_004937.1
DOK2	Docking protein 2	NM_003974.2	NP_003965.2
DUSP22	Dual specificity phosphatase 22	NM_020185.3	NP_064570.1
EVI2B	Ecotropic viral integration site 2B	NM_006495.3	NP_006486.3
FCER1A	Fc fragment of IgE, high affinity I, receptor for; alpha polypeptide	NM_002001.2	NP_001992.1
FLT3	Fms-related tyrosine kinase 3	NM_004119.2	NP_004110.2
FMNL1	Formin-like 1	NM_005892.3	NP_005883.2
FNBP1	Formin binding protein 1	NM_015033.2	NP_055848.1
FOSB	FBJ murine osteosarcoma viral oncogene homolog B	NM_001114171.1	NP_001107643.1
GATA2	GATA binding protein 2	NM_001145661.1	NP_001139133.1
GMFG	Glia maturation factor, gamma	NM_004877.2	NP_004868.1
GNA15	Guanine nucleotide binding protein, alpha 15	NM_002068.2	NP_002059.2
HMHA-1	Histocompatibility minor HA-1	NM_012292.2	NP_036424.2
HOXA9	Homeobox A9	NM_152739.3	NP_689952.1
HSPA6	Heat shock 70kDa protein 6	NM_002155.3	NP_002146.2
ICAM3	Intercellular adhesion molecule 3	NM_002162.3	NP_002153.2
IL2RG	Interleukin 2 receptor, gamma	NM_000206.2	NP_000197.1
IQGAP2	IQ motif containing GTPase activating protein 2	NM_006633.2	NP_006624.2
ISG20	Interferon stimulated exonuclease gene 20kDa	NM_002201.4	NP_002192.2
ITGAL	Integrin, alpha L	NM_001114380.1	NP_001107852.1
ITGAM	Integrin, alpha M	NM_000632.3	NP_000623.2
ITGB2	Integrin, beta 2	NM_000211.3	NP_000202.2
KCNAB2	Voltage-gated potassium channel subunit beta-2 isoform 1	NM_003636.2	NP_003627.1
LAT2	Linker for activation of T-cells family member 2	NM_014146.3	NP_054865.2
LCP2	Lymphocyte cytosolic protein 2	NM_005565.3	NP_005556.1
LOC81691	Putative RNA exonuclease NEF-sp isoform 2	NM_001144924.1	NP_001138396.1
LRMP	Lymphoid-restricted membrane protein	NM_006152.2	NP_006143.2
LTB	Lymphotoxin-beta isoform a	NM_002341.1	NP_002332.1
LYN	Tyrosine-protein kinase Lyn isoform B	NM_001111097.1	NP_001104567.1
MAP4K1	Mitogen-activated protein kinase kinase kinase kinase 1 isoform 1	NM_001042600.1	NP_001036065.1
MCM5	DNA replication licensing factor MCM5	NM_006739.3	NP_006730.2
MPL	Thrombopoietin receptor precursor	NM_005373.2	NP_005364.1
NCF4	Neutrophil cytosol factor 4 isoform 1	NM_000631.4	NP_000622.2
NUP210	Nuclear pore membrane glycoprotein 210 precursor	NM_024923.2	NP_079199.2
PIK3CD	Phosphatidylinositol-4,5-bisphosphate 3-kinase catalytic subunit delta isoform	NM_005026.3	NP_005017.3
PIM2	Serine/threonine-protein kinase pim-2	NM_006875.3	NP_006866.2
PLCB2	1-Phosphatidylinositol-4,5-bisphosphate phosphodiesterase beta-2	NM_004573.2	NP_004564.2
PLEK	Pleckstrin	NM_002664.2	NP_002655.2
PRKCB1	Protein kinase C beta type isoform 2	NM_002738.6	NP_002729.2
PSD4	PH and SEC7 domain-containing protein 4	NM_012455.2	NP_036587.2
PSMB10	Proteasome subunit beta type-10 proprotein	NM_002801.2	NP_002792.1
PSMB8	Proteasome subunit beta type-8 isoform E1 proprotein	NM_004159.4	NP_004150.1
PTPN22	Tyrosine-protein phosphatase non-receptor type 22 isoform 3	NM_001193431.1	NP_001180360.1
PTPN6	Tyrosine-protein phosphatase non-receptor type 6 isoform 1	NM_002831.5	NP_002822.2
PTPRC	Receptor-type tyrosine-protein phosphatase C isoform 1 precursor	NM_002838.3	NP_002829.2
PTPRCAP	Protein tyrosine phosphatase receptor type C-associated protein	NM_005608.2	NP_005599.1
RASGRP2	RAS guanyl-releasing protein 2	NM_153819.1	NP_722541.1
RGS1	Regulator of G-protein signaling 1	NM_002922.3	NP_002913.3
SELL	L-selectin precursor	NM_000655.4	NP_000646.2
SELPLG	P-selectin glycoprotein ligand 1	NM_003006.3	NP_002997.1
SEPT6	Septin-6 isoform B	NM_015129.5	NP_055944.2
SF1	Splicing factor 1 isoform 6	NM_001178030.1	NP_001171501.1
SOCS2	Suppressor of cytokine signaling 2	NM_003877.3	NP_003868.1
SP110	Sp110 nuclear body protein isoform d	NM_001185015.1	NP_001171944.1
SYNGR1	Synaptogyrin-1 isoform 1a	NM_004711.4	NP_004702.2
TNRC5	Protein canopy homolog 3 precursor	NM_006586.3	NP_006577.2
ZFP36L2	Zinc finger protein 36, C3H1 type-like 2	NM_006887.4	NP_008818.3
