# Supplementary material for: High-Throughput Identification of Potential Minor Histocompatibility Antigens by MHC Tetramer-Based Screening: Feasibility and Limitations
Source: PLoS One. 2011 Aug 5;6(8):e22523. doi: 10.1371/journal.pone.0022523 (PMC3151248; doi:10.1371/journal.pone.0022523)
Supplement: Table S2 — Total MiHA epitopes predicted by HLA-peptide binding algorithm. (PDF) [file pone.0022523.s004.pdf]

Supplementary Table II. Total MiHA epitopes predicted by HLA-peptide binding algorithm

Gene symbol	Residue Change	RefSNP accession	dbSNP AH [α]	Minor Frequency [β]	Reading Frame	Length	Peptide Sequence [γ]
AIF	RG 14	rs2736182	0,22	0,01	NRF	10	D L Q G G K A F G L
AIF	RG 14	rs2736182	0,22	0,01	NRF	10	D L Q G G K A F R L
AIF	RG 14	rs2736182	0,22	0,01	NRF	11	G L L K A Q Q E E R L
AIF	RG 14	rs2736182	0,22	0,01	NRF	9	L Q G G K A F G L
AIF	RG 14	rs2736182	0,22	0,01	NRF	9	L Q G G K A F R L
AIF	RG 14	rs2736182	0,22	0,01	NRF	11	R L L K A Q Q E E R L
AREG	PP 10	rs1615111	0,18	0,02	ARF	9	A G A G G A V A L
AREG	PP 10	rs1615111	0,18	0,02	ARF	9	A G A S G A V A L
AREG	PP 10	rs1615111	0,18	0,02	ARF	9	A T A G A G G A V
AREG	PP 10	rs1615111	0,18	0,02	ARF	9	A T A G A S G A V
AREG	PP 10	rs1615111	0,18	0,02	ARF	11	A T A G A S G A V A L
AREG	PT 76	rs7656521	0,09	0,01	NRF	10	A A H P P G L H S L
AREG	PT 76	rs7656521	0,09	0,01	NRF	10	A A H T P G L H S L
AREG	PT 76	rs7656521	0,09	0,01	NRF	9	A H P P G L H S L
AREG	PT 76	rs7656521	0,09	0,01	NRF	9	A H T P G L H S L
AREG	FF 177	rs2291715	0,22	N.D	ARF	9	I L V N G V G K S
AREG	FF 177	rs2291715	0,22	N.D	ARF	11	N V S K N I L V N G V
AREG	FF 177	rs2291715	0,22	N.D	ARF	11	N V S K N I S V N G V
AREG	FF 177	rs2291715	0,22	N.D	ARF	9	S K N I L V N G V
AREG	FF 177	rs2291715	0,22	N.D	ARF	9	S K N I S V N G V
ARHGAP4	VA 104	rs5987182	0,14	0,00	NRF	9	L L S P L H C W A
ARHGAP4	VA 104	rs5987182	0,14	0,00	NRF	10	L L S P L H C W A V
ARHGAP4	VA 104	rs5987182	0,14	0,00	NRF	11	L L S P L H C W A V L
ARHGAP4	VA 104	rs5987182	0,14	0,00	NRF	9	L L S P L H C W V
ARHGAP4	VA 104	rs5987182	0,14	0,00	NRF	10	L L S P L H C W V V
ARHGAP4	VA 104	rs5987182	0,14	0,00	NRF	11	L L S P L H C W V V L
ARHGAP4	VA 104	rs5987182	0,14	0,00	NRF	9	L S P L H C W A V
ARHGAP4	VA 104	rs5987182	0,14	0,00	NRF	9	P L H C W A V L L
ARHGAP4	VA 104	rs5987182	0,14	0,00	NRF	9	P L H C W V V L L
ARHGAP4	VA 104	rs5987182	0,14	0,00	NRF	10	S L L S P L H C W A
ARHGAP4	VA 104	rs5987182	0,14	0,00	NRF	11	S L L S P L H C W A V
ARHGAP4	VA 104	rs5987182	0,14	0,00	NRF	10	S L L S P L H C W V
ARHGAP4	VA 104	rs5987182	0,14	0,00	NRF	11	S L L S P L H C W V V
ARHGAP4	VA 104	rs5987182	0,14	0,00	NRF	10	S P L H C W V V L L
ARHGAP4	VV 523	rs2070098	N.D.	0,00	ARF	9	A C A P G G G E L
ARHGAP4	VV 523	rs2070098	N.D.	0,00	ARF	9	A C A P G R G E L
ARHGAP4	VV 523	rs2070098	N.D.	0,00	ARF	10	A P G G G E L H S L
ARHGAP4	VV 523	rs2070098	N.D.	0,00	ARF	10	A P G R G E L H S L
ARHGAP4	VV 523	rs2070098	N.D.	0,00	ARF	10	S L C P W S W R A A
ARHGAP4	VV 523	rs2070098	N.D.	0,00	ARF	10	S L C P W W W R A A
ARHGAP15	LF 438	rs11538443	N.D.	0,00	NRF	11	F L R A E N E T G N M
ARHGAP15	LF 438	rs11538443	N.D.	0,00	NRF	9	G I V F G P T F L
ARHGAP15	LF 438	rs11538443	N.D.	0,00	NRF	9	G I V F G P T L L
ARHGAP15	LF 438	rs11538443	N.D.	0,00	NRF	10	I V F G P T F L R A
ARHGAP15	LF 438	rs11538443	N.D.	0,00	NRF	10	I V F G P T L L R A
ARHGAP15	LF 438	rs11538443	N.D.	0,00	ARF	10	K L G D C I W T Y L
ARHGAP15	LF 438	rs11538443	N.D.	0,00	ARF	11	K L G D C I W T Y L S
ARHGAP15	LF 438	rs11538443	N.D.	0,00	ARF	11	K L G D C I W T Y P S
ARHGAP15	LF 438	rs11538443	N.D.	0,00	NRF	11	L L R A E N E T G N M
ARHGAP15	LF 438	rs11538443	N.D.	0,00	NRF	10	S L G I V F G P T L
ARHGAP15	LF 438	rs11538443	N.D.	0,00	NRF	9	T L L R A E N E T
ARHGAP15	LF 438	rs11538443	N.D.	0,00	NRF	11	T L L R A E N E T G N
ARHGAP25	AA 384	rs17604346	0,01	0,02	ARF	9	M L L K T S E F L
ARHGAP25	AA 384	rs17604346	0,01	0,02	ARF	11	M L L K T S E F L G Q
ARHGAP25	RS 555	rs4241344	0,43	0,27	NRF	10	K L I L C R V W S K
ARHGAP25	RS 555/MT 556	Combination	0,43/0,44	0,27/N.D.	NRF	9	S L Q S T V Q E L
ARHGAP25	RS 555	rs4241344	0,43	0,27	NRF	9	S L Q R M V Q E L
ARHGAP25	RS 555	rs4241344	0,43	0,27	NRF	9	S L Q S M V Q E L
ARHGAP25	RS 555	rs4241344	0,43	0,27	NRF	11	S L Q S M V Q E L R K
ARHGAP25	RS 555	rs4241344	0,43	0,27	NRF	10	S M V Q E L R K E I
ARHGAP25	MT 556	rs10177248	0,44	N.D.	NRF	9	M V Q E L R K E I
ARHGAP25	MT 556	rs10177248	0,44	N.D.	NRF	10	R M V Q E L R K E I
ARHGAP25	MT 556	rs10177248	0,44	N.D.	NRF	9	S L Q R T V Q E L
ARHGAP25	MT 556	rs10177248	0,44	N.D.	NRF	9	T V Q E L R K E I
ATP2A3	W*stop 77	rs17846878	N.D.	0,00	NRF	9	A L V S F V L A W
ATP2A3	W*stop 77	rs17846878	N.D.	0,00	ARF	9	L L C P G L I R G
ATP2A3	W*stop 77	rs17846878	N.D.	0,00	ARF	10	L L C P G L I R G G
ATP2A3	W*stop 77	rs17846878	N.D.	0,00	ARF	9	L L C P G L V R G
ATP2A3	W*stop 77	rs17846878	N.D.	0,00	ARF	10	L L C P G L V R G G
ATP2A3	LL 302	rs9915542	N.D.	0,00	ARF	10	C L L L Q D R R G L
ATP2A3	LL 302	rs9915542	N.D.	0,00	ARF	9	L L L Q D R R G L
ATP2A3	AA 424	rs1800911	N.D.	0,44	ARF	10	R L W T T T R P R V
ATP2A3	ND 458	rs9913158	N.D.	0,00	NRF	9	K M N V F D T D L
ATP2A3	ND 458	rs9913158	N.D.	0,00	NRF	9	K M N V F D T N L
ATP2A3	ND 458	rs9913158	N.D.	0,00	NRF	9	N V F D T D L Q A
ATP2A3	ND 458	rs9913158	N.D.	0,00	NRF	10	N V F D T D L Q A L
ATP2A3	ND 458	rs9913158	N.D.	0,00	NRF	9	N V F D T N L Q A
ATP2A3	ND 458	rs9913158	N.D.	0,00	NRF	10	N V F D T N L Q A L
ATP2A3	ND 458	rs9913158	N.D.	0,00	NRF	9	T N L Q A L S R V
ATP2A3	DE 519	rs12946879	N.D.	0,00	NRF	11	K M F V K G A P D S V
ATP2A3	DE 519	rs12946879	N.D.	0,00	NRF	11	K M F V K G A P E S V
ATP2A3	DE 519	rs12946879	N.D.	0,00	NRF	11	M F V K G A P E S V I
ATP2A3	CR 674	rs9895012	N.D.	0,07	ARF	10	L L L R P R G A R T

ATP2A3	CR 674	rs9895012	N.D.	0,07	ARF	11	L L L R P R G A R T Q
ATP2A3	CR 674	rs9895012	N.D.	0,07	NRF	9	R T A C C F A R V
ATP2A3	CR 674	rs9895012	N.D.	0,07	NRF	9	R T A R C F A R V
ATP2A3	HQ 869	rs11654827	0,01	0,00	NRF	10	I N F Y H L R N F L
ATP2A3	HQ 869	rs11654827	0,01	0,00	NRF	10	I N F Y Q L R N F L
ATP2A3	RR 1034	rs887387	0,43	0,34	ARF	9	G T G W S L R C V
ATP2A3	RR 1034	rs887387	0,43	0,34	ARF	9	W E Q G G V S G V
ATP2A3	RR 1034	rs887387	0,43	0,34	ARF	9	W E Q S G V S G V
BTK	SS 318	rs5991926	N.D.	0,00	ARF	10	C L C L L N P Q G T
BTK	SS 318	rs5991926	N.D.	0,00	ARF	10	K L A N I Q C L C L
BTK	SS 318	rs5991926	N.D.	0,00	ARF	10	K L A N I Q C P C L
BTK	SS 318	rs5991926	N.D.	0,00	ARF	10	L A N I Q C L C L L
BTK	SS 318	rs5991926	N.D.	0,00	ARF	10	Q S W Q I Y S V C V
BTK	SS 318	rs5991926	N.D.	0,00	ARF	10	Q S W Q I Y S V R V
BTK	CY 361	rs28935478	N.D.	0,00	NRF	11	E L I N Y H Q H N S A
BTK	CY 361	rs28935478	N.D.	0,00	ARF	11	S L S S L T A I S T T
BTK	CY 361	rs28935478	N.D.	0,00	ARF	10	S L S S L T A I S T
BTK	CY 361	rs28935478	N.D.	0,00	ARF	11	S L S S L T T I S T T
BTK	CY 361	rs28935478	N.D.	0,00	ARF	10	S L S S L T T I S T
BTK	CY 361	rs28935478	N.D.	0,00	ARF	11	S L T A I S T T L Q D
BTK	CY 361	rs28935478	N.D.	0,00	ARF	9	S L T A I S T T L
BTK	CY 361	rs28935478	N.D.	0,00	ARF	9	S L T T I S T T L
BTK	CC 633	rs1135363	N.D.	0,21	ARF	11	T V A G M R K Q M S V
BTK	CC 633	rs1135363	N.D.	0,21	ARF	11	T V V G M R K Q M S V
BTK	CC 633	rs1135363	N.D.	0,21	ARF	10	V V G M R K Q M S V
BTK	CC 633	rs1135363	N.D.	0,21	ARF	10	Y I P S C T V A G M
CCL3	PP 60	rs1130371	0,43	0,04	ARF	11	T L R R A A S A P S L
CCL3	GS 69	rs5029408	N.D.	0,07	NRF	9	F L T K R G R Q V
CCL3	GS 69	rs5029408	N.D.	0,07	NRF	9	F L T K R S R Q V
CCL3	GS 69	rs5029408	N.D.	0,07	NRF	11	F L T K R S R Q V C A
CD37	CC 7	rs11545491	N.D.	0,00	ARF	11	E L P Q P H Q V L P L
CD37	CC 7	rs11545491	N.D.	0,00	ARF	11	E L S Q P H Q V L P L
CD37	CC 7	rs11545491	N.D.	0,00	ARF	10	L P Q P H Q V L P L
CD37	CC 7	rs11545491	N.D.	0,00	ARF	10	L S Q P H Q V L P L
CD37	II 38	rs354021	0,21	0,18	ARF	10	H L D P H R Q D Q L
CD48	LL 16	rs11541827	N.D.	0,01	ARF	9	F V S G S G I A I
CD48	LL 16	rs11541827	N.D.	0,01	ARF	10	F V S G S G I A I A
CD48	LL 16	rs11541827	N.D.	0,01	ARF	9	F V S G S G I A T
CD48	LL 16	rs11541827	N.D.	0,01	ARF	10	F V S G S G I A T A
CD48	QE 102	rs2295615	0,26	N.D.	ARF	10	A L Y I S K V Q K E
CD48	QE 102	rs2295615	0,26	N.D.	ARF	10	A L Y I S K V Q K Q
CD48	QE 102	rs2295615	0,26	N.D.	ARF	11	K Q D N S T Y I M R V
CD48	QE 102	rs2295615	0,26	N.D.	ARF	11	K V Q K E D N S T Y I
CD48	QE 102	rs2295615	0,26	N.D.	ARF	11	K V Q K Q D N S T Y I
CD48	LL 150	rs17851383	N.D.	0,00	ARF	9	L L F E T V M C D
CD48	LL 150	rs17851383	N.D.	0,00	ARF	10	L L F E T V M C D T
CD48	LL 150	rs17851383	N.D.	0,00	ARF	10	L L S E T V M C D T
CD52	SN 40	rs1071849	0,46	0,28	NRF	11	N I S G G I F L F F V
CD52	SN 40	rs1071849	0,46	0,28	NRF	11	Q T S S P S A S S S I
CD52	SN 40/MI 41	Combination	0,46/0,46	0,28/N.D.	NRF	11	Q T S S P S A S S S M
CD52	SN 40	rs1071849	0,46	0,28	NRF	11	S I S G G I F L F F V
CD52	MI 41	rs17645	0,46	N.D.	NRF	10	I S G G I F L F F V
CD52	MI 41	rs17645	0,46	N.D.	NRF	10	M S G G I F L F F V
CD52	MI 41	rs17645	0,46	N.D.	NRF	11	N M S G G I F L F F V
CD52	MI 41	rs17645	0,46	N.D.	NRF	11	Q T S S P S A S S N I
CD52	MI 41	rs17645	0,46	N.D.	NRF	11	Q T S S P S A S S N M
CD69	NN 166	rs11052883	0,066	0,04	ARF	9	K E F N N W F K V
CD69	NN 166	rs11052883	0,066	0,04	ARF	9	K E F N N W F N V
CD69	NN 166	rs11052883	0,066	0,04	ARF	9	K V T G S D K C V
CD69	NN 166	rs11052883	0,03	0,04	ARF	10	L T T G S T L Q G L
CD69	NN 166	rs11052883	0,03	0,04	ARF	10	L T T G S K L Q G L
CD69	NN 166	rs11052883	0,03	0,04	ARF	11	N L T T G S T L Q G L
CD69	NN 166	rs11052883	0,066	0,04	ARF	11	N L T T G S K L Q G L
CD69	NN 166	rs11052883	0,066	0,04	ARF	9	N V T G S D K C V
CD69	NN 166	rs11052883	0,03	0,04	ARF	11	Q M A K N L T T G S T
CD69	NN 166	rs11052883	0,066	0,04	ARF	9	S K L Q G L T S V
CD69	NN 166	rs11052883	0,03	0,04	ARF	9	S T L Q G L T S V
CD69	NN 166	rs11052883	0,03	0,04	ARF	11	S T L Q G L T S V F F
CD69	NN 166	rs11052883	0,03	0,04	ARF	9	T T G S T L Q G L
CD69	NN 166	rs11052883	0,03	0,04	ARF	9	T T G S K L Q G L
CD79b	LL 20	rs11555057	N.D.	0,00	ARF	9	P L D G G V A A A
CD79b	LL 20	rs11555057	N.D.	0,00	ARF	9	P L D G G V A A V
CD79b	LL 20	rs11555057	N.D.	0,00	ARF	10	Q P L D G G V A A V
CD79b	CC 122	rs2070776	0,47	0,27	ARF	10	G L R T M A S T S V
CD79b	CC 122	rs2070776	0,47	0,27	ARF	10	L L L P A E V Q Q H
CD79b	CC 122	rs2070776	0,47	0,27	ARF	11	L L L P A E V Q Q H L
CD79b	CC 122	rs2070776	0,47	0,27	ARF	11	L L L S A E V Q Q H L
CD79b	CC 122	rs2070776	0,47	0,27	ARF	10	L L P A E V Q Q H L
CD79b	CC 122	rs2070776	0,47	0,27	ARF	10	L L S A E V Q Q H L
CD79b	CC 122	rs2070776	0,47	0,27	ARF	11	S G L R T M A S T S V
CD79b	CC 122	rs2070776	0,47	0,27	ARF	11	T M A S T S A S R S A
CD79b	CC 122	rs2070776	0,47	0,27	ARF	11	T M A S T S V S R S A
CD79b	KE 217	rs1063625	N.D.	0,00	NRF	10	D I V T L R T G E V
CD79b	KE 217	rs1063625	N.D.	0,00	NRF	10	D I V T L R T G K V
CD79b	KE 217	rs1063625	N.D.	0,00	NRF	9	R T G E V K W S V
CD79b	KE 217	rs1063625	N.D.	0,00	NRF	9	R T G K V K W S V
CD79b	KE 217	rs1063625	N.D.	0,00	NRF	11	T L R T G E V K W S V
CENTB1	YY 166	rs3809828	0,13	0,07	ARF	9	W V P G T G T G L

CENTB1	YY 166	rs3809828	0,13	0,07	ARF	9	WVSGTGTGL
CORO1A	DH 80	rs11555342	N.D.	0,00	ARF	9	GLWPHSPCA
CORO1A	DH 80	rs11555342	N.D.	0,00	ARF	9	GLWRHSPCA
CORO1A	DH 80	rs11555342	N.D.	0,00	NRF	9	TVCGDTAPV
CORO1A	DH 80	rs11555342	N.D.	0,00	NRF	9	TVCGHTAPV
CORO1A	LL 404	rs11555339	N.D.	0,00	ARF	10	WLRTPKEPGV
CORO1A	PT 443	rs1053574	0,04	0,00	NRF	9	EMRKLQATV
CORO1A	PT 443	rs1053574	0,04	0,00	NRF	9	KLQAPVQEL
CORO1A	PT 443	rs1053574	0,04	0,00	NRF	9	KLQATVQEL
CORO1A	PT 443	rs1053574	0,04	0,00	NRF	9	TVQELQKRL
CPVL	QP 112	rs11542982	N.D.	0,00	NRF	10	GQGGSSMFGL
CPVL	RH 398	rs1052200	0,3	0,15	NRF	9	AAALTEHSL
CPVL	RH 398	rs1052200	0,3	0,15	NRF	9	AAALTERSL
CPVL	RH 398	rs1052200	0,3	0,15	NRF	10	ALTEHSLMGM
CPVL	RH 398	rs1052200	0,3	0,15	NRF	10	ALTERSLMGM
CPVL	RH 398	rs1052200	0,3	0,15	NRF	11	IVAAALTEHSL
CPVL	RH 398	rs1052200	0,3	0,15	NRF	11	IVAAALTERSL
CPVL	VA 435	rs7313	0,47	0,42	NRF	9	EVAGYIRQV
CPVL	VA 435	rs7313	0,47	0,42	ARF	11	LVTSGKRVTSI
CPVL	VA 435	rs7313	0,47	0,42	ARF	9	LVTSGKWVT
CPVL	VA 435	rs7313	0,47	0,42	NRF	9	RQAGDFHQV
CPVL	VA 435	rs7313	0,47	0,42	NRF	9	RQVGDFHQV
CPVL	VA 435	rs7313	0,41	0,42	ARF	10	RVTSIRLFEV
CPVL	VA 435	rs7313	0,47	0,42	ARF	10	VTSGKRVTSI
CPVL	VA 435	rs7313	0,47	0,42	ARF	10	VTSGKWVTSI
CPVL	VA 435	rs7313	0,41	0,42	ARF	10	WVTSIRLFEV
CPVL	VA 435	rs7313	0,47	0,42	ARF	9	WLVTSGKRV
CPVL	VA 435	rs7313	0,47	0,42	ARF	10	WLVTSGKRVT
CPVL	VA 435	rs7313	0,47	0,42	ARF	9	WLVTSGKWV
CPVL	VA 435	rs7313	0,47	0,42	ARF	10	WLVTSGKWVT
CSF3R	VV 36	rs3918011	0,01	0,01	ARF	11	FTWGIPSQPPA
CSF3R	VV 36	rs3918011	0,01	0,01	ARF	10	SQPPSFTWGI
CSF3R	VV 36	rs3918011	0,01	0,01	ARF	10	SQPPSSTWGI
CSF3R	VV 36	rs3918011	0,01	0,01	ARF	11	STWGIPSQPPA
CSF3R	VV 36	rs3918011	0,01	0,01	ARF	9	SVSQPPSFT
CSF3R	AA 242	rs3918017	0,02	N.D	ARF	11	LLPRQAAYSCA
CSF3R	ND 320	rs3918018	0,07	0,13	ARF	9	APAWPLEQL
CSF3R	ND 320	rs3918018	0,07	0,13	ARF	9	APAWPLERL
CSF3R	ND 320	rs3918018	0,07	0,13	ARF	10	LAPAWPLEQL
CSF3R	ND 320	rs3918018	0,07	0,13	ARF	10	LAPAWPLERL
CSF3R	RQ 346	rs3917974	0,04	N.D	NRF	9	QLDPRTVQL
CSF3R	RQ 346	rs3917974	0,04	N.D	NRF	10	RQLDPRTVQL
CSF3R	RQ 346	rs3917974	0,04	N.D	NRF	9	RLDPRTVQL
CSF3R	RR 418	rs3917980	0,4	N.D.	ARF	10	ITQPGPLAPL
CSF3R	RR 418	rs3917980	0,4	N.D.	ARF	10	ITQPGPLVPL
CSF3R	RR 418	rs3917980	0,4	N.D.	ARF	9	TQPGPLAPL
CSF3R	RR 418	rs3917980	0,4	N.D.	ARF	9	TQPGPLVPL
CSF3R	QR 440	rs3918020	0,35	0,00	NRF	11	AMAQDPHSLWV
CSF3R	QR 440	rs3918020	0,35	0,00	NRF	11	AMARDPHSLWV
CSF3R	QR 440	rs3918020	0,35	0,00	NRF	9	AMAQDPHSL
CSF3R	QR 440	rs3918020	0,35	0,00	NRF	9	AMARDPHSL
CSF3R	QR 440	rs3918020	0,35	0,00	NRF	9	AQDPHSLWV
CSF3R	QR 440	rs3918020	0,35	0,00	NRF	10	MAQDPHSLWV
CSF3R	QR 440	rs3918020	0,35	0,00	NRF	10	MARDPHSLWV
CSF3R	HD 510	rs3917991	0,12	0,02	ARF	9	LVPGHHGTL
CSF3R	HD 510	rs3917991	0,12	0,02	ARF	9	SLVPAHHGT
CSF3R	HD 510	rs3917991	0,12	0,02	ARF	10	SLVPAHHGTL
CSF3R	HD 510	rs3917991	0,12	0,02	ARF	9	SLVPGHHGT
CSF3R	HD 510	rs3917991	0,12	0,02	ARF	10	SLVPGHHGTL
DOCK2	QQ 784	rs13155521	0,05	0,06	ARF	9	SIQNYHPFA
DOCK2	YY 1438	rs17647491	0,02	0,04	ARF	10	IQLCAKVPLL
DOCK2	YY 1438	rs17647491	0,02	0,04	ARF	9	IQLCAKVPL
DOCK2	YY 1438	rs17647491	0,02	0,04	ARF	10	IQLRAKVPLL
DOCK2	YY 1438	rs17647491	0,02	0,04	ARF	9	IQLRAKVPL
DOCK2	YY 1438	rs17647491	0,02	0,04	ARF	9	KLLQIQLCA
DOCK2	YY 1438	rs17647491	0,02	0,04	ARF	11	KLLQIQLCAKV
DOCK2	YY 1438	rs17647491	0,02	0,04	ARF	11	KLLQIQLRAKV
DOCK2	YY 1438	rs17647491	0,02	0,04	ARF	9	KLLQIQLRA
DOCK2	YY 1438	rs17647491	0,02	0,04	ARF	10	LLQIQLCAKV
DOCK2	YY 1438	rs17647491	0,02	0,04	ARF	10	LLQIQLRAKV
DOCK2	YY 1438	rs17647491	0,02	0,04	ARF	9	LQIQLCAKV
DOCK2	YY 1438	rs17647491	0,02	0,04	ARF	9	LQIQLRAKV
DOCK2	YY 1438	rs17647491	0,02	0,04	ARF	10	QIQLCAKVPL
DOCK2	YY 1438	rs17647491	0,02	0,04	ARF	10	QIQLRAKVPL
DOCK2	YY 1438	rs17647491	0,02	0,04	ARF	9	QLCAKVPLL
DOCK2	YY 1438	rs17647491	0,02	0,04	ARF	9	QLRAKVPLL
DOCK2	YY 1438	rs17647491	0,018	0,04	ARF	9	RLTSTNPTM
DOCK2	YY 1438	rs17647491	0,018	0,04	ARF	9	RLTSTNPTT
DOCK2	RR 1632	rs1045168	0,34	0,29	ARF	10	LTLTTGEWAA
DOCK2	RR 1632	rs1045168	0,34	0,29	ARF	10	LTLTTGEWAV
DOCK2	RR 1632	rs1045168	0,34	0,29	ARF	9	TLTTGEWAA
DOCK2	RR 1632	rs1045168	0,34	0,29	ARF	9	TLTTGEWAV
DOCK2	RR 1632	rs1045168	0,34	0,29	ARF	10	TTGEWAVPGL
DOK2	PA 152	rs1140295	N.D.	0,00	ARF	10	SQSAPTRNFL
DOK2	PA 152	rs1140295	N.D.	0,00	NRF	10	TVGPHKEFAV
DOK2	PA 152	rs1140295	N.D.	0,00	NRF	10	TVGPHKEFPV
DOK2	PA 152	rs1140295	N.D.	0,00	NRF	9	VGPHKEFAV
DOK2	PA 152	rs1140295	N.D.	0,00	NRF	9	VGPHKEFPV

DOK2	PA 152	rs1140295	N.D.	0,00	NRF	11	V T V G P H K E F A V
DOK2	PA 152	rs1140295	N.D.	0,00	NRF	11	V T V G P H K E F P V
DOK2	PT 336	rs7824139	N.D.	0,00	ARF	10	A L R R P C P L D L
DOK2	PT 336	rs7824139	N.D.	0,00	ARF	10	A L R S P C P L D L
DOK2	PT 336	rs7824139	N.D.	0,00	NRF	10	P L Y D S I E E P L
DOK2	PT 336	rs7824139	N.D.	0,00	NRF	9	P L Y D S I E E T
DOK2	PT 336	rs7824139	N.D.	0,00	NRF	10	P L Y D S I E E T L
DOK2	PT 336	rs7824139	N.D.	0,00	NRF	9	T L P P R P D H I
DOK2	AA 387	rs17853066	0,03	N.D.	ARF	10	L L A S S M S S Q L
DOK2	AA 387	rs17853066	0,03	N.D.	ARF	11	L L A S S M S S Q L G
DOK2	AA 387	rs17853066	0,03	N.D.	ARF	9	Q L G R I S L L L
DOK2	AA 387	rs17853066	0,03	N.D.	ARF	10	Q L G R I S L L L A
DOK2	AA 387	rs17853066	0,03	N.D.	ARF	9	R A G F L C F W L
DOK2	AA 387	rs17853066	0,03	N.D.	ARF	11	S M S S Q L G R I S L
DOK2	AA 387	rs17853066	0,03	N.D.	ARF	9	S M S S Q L G R I
DOK2	AA 387	rs17853066	0,03	N.D.	ARF	11	S M S S Q P G R I S L
DOK2	AA 387	rs17853066	0,03	N.D.	ARF	9	S Q L G R I S L L
DOK2	AA 387	rs17853066	0,03	N.D.	ARF	10	S Q L G R I S L L L
DOK2	AA 387	rs17853066	0,03	N.D.	ARF	11	T L L A S S M S S Q L
DOK2	AA 387	rs17853066	0,03	N.D.	ARF	9	W A G F L C F W L
DOK2	AS 394	rs2242241	0,01	0,30	ARF	9	R A G F L C F W L
DUSP22	HR 119	rs7768224	0,07	0,00	ARF	9	C M L G D P V P T
DUSP22	HR 119	rs7768224	0,07	0,00	ARF	10	M L G D P V P T P T
DUSP22	HR 119	rs7768224	0,07	0,00	ARF	9	R M P C T P C M L
DUSP22	HR 119	rs7768224	0,07	0,00	ARF	9	R M P C T P C V L
DUSP22	HR 119	rs7768224	0,07	0,00	ARF	10	V L G D P V P T P T
DUSP22	PP 172	rs1129085	0,24	0,37	ARF	9	S G N S E V L G L
DUSP22	PP 172	rs1129085	0,24	0,37	ARF	9	S R N S E V L G L
EVI2B	VI 161	rs17882145	0,01	0,00	NRF	10	Q I P S R K Q V T V
EVI2B	VI 161	rs17882145	0,01	0,00	NRF	10	S V Q I P S R K Q V
EVI2B	VI 161	rs17882145	0,01	0,00	NRF	9	V Q I P S R K Q V
EVI2B	GC 340	rs17884293	0,01	0,00	ARF	9	C L D H L P F W I
EVI2B	GC 340	rs17884293	0,01	0,00	ARF	9	C L H H L P F W I
EVI2B	GC 340	rs17884293	0,01	0,00	NRF	9	G L P P P P P L L
EVI2B	GC 340	rs17884293	0,01	0,00	NRF	10	L P P P P P L L D L
EVI2B	GC 340	rs17884293	0,01	0,00	ARF	9	M M Q V C L H H L
EVI2B	GC 340	rs17884293	0,01	0,00	ARF	10	V C L H H L P F W I
FCER1A	VA 230	rs17851282	N.D.	0,00	NRF	9	F I S T Q Q Q A T
FCER1A	VA 230	rs17851282	N.D.	0,00	NRF	11	F I S T Q Q Q A T F L
FCER1A	VA 230	rs17851282	N.D.	0,00	NRF	11	F I S T Q Q Q V T F L
FCER1A	VA 230	rs17851282	N.D.	0,00	NRF	10	G L F I S T Q Q Q A
FCER1A	VA 230	rs17851282	N.D.	0,00	NRF	10	G L F I S T Q Q Q V
FCER1A	VA 230	rs17851282	N.D.	0,00	NRF	11	I S T Q Q Q A T F L L
FCER1A	VA 230	rs17851282	N.D.	0,00	NRF	11	I S T Q Q Q V T F L L
FCER1A	VA 230	rs17851282	N.D.	0,00	NRF	9	Q Q A T F L L K I
FCER1A	VA 230	rs17851282	N.D.	0,00	NRF	9	Q Q V T F L L K I
FCER1A	VA 230	rs17851282	N.D.	0,00	NRF	9	S T Q Q Q V T F L
FCER1A	VA 230	rs17851282	N.D.	0,00	NRF	9	T Q Q Q A T F L L
FCER1A	VA 230	rs17851282	N.D.	0,00	NRF	9	T Q Q Q V T F L L
FLT3	GD 7	rs12872889	0,5	N.D	NRF	9	A L A R D A G T V
FLT3	GD 7	rs12872889	0,5	N.D	NRF	11	A L A R D A G T V P L
FLT3	GD 7	rs12872889	0,5	N.D	NRF	9	A L A R G A G T V
FLT3	GD 7	rs12872889	0,5	N.D	NRF	11	A L A R G A G T V P L
FLT3	GD 7	rs12872889	0,5	N.D	NRF	10	G A G T V P L L V V
FLT3	GD 7	rs12872889	0,5	N.D	NRF	10	L A R D A G T V P L
FLT3	GD 7	rs12872889	0,5	N.D	NRF	10	L A R G A G T V P L
FLT3	GD 7	rs12872889	0,5	N.D	NRF	9	R D A G T V P L L
FLT3	GD 7	rs12872889	0,5	N.D	NRF	9	R G A G T V P L L
FLT3	DD 96	rs7338903	0,08	0,22	ARF	9	S M P Q G T F P V
FLT3	DD 96	rs7338903	0,08	0,22	ARF	9	S T P Q G T F P V
FLT3	DD 96	rs7338903	0,08	0,22	ARF	10	W S M P Q G T F P V
FLT3	DD 96	rs7338903	0,08	0,22	ARF	10	W S T P Q G T F P V
FLT3	MT 227	rs1933437	0,49	0,41	NRF	9	K V L H E L F G M
FLT3	MT 227	rs1933437	0,49	0,41	NRF	11	K V L H E L F G M D I
FLT3	MT 227	rs1933437	0,49	0,41	NRF	9	K V L H E L F G T
FLT3	MT 227	rs1933437	0,49	0,41	NRF	11	K V L H E L F G T D I
FLT3	MT 227	rs1933437	0,49	0,41	NRF	10	V L H E L F G M D I
FLT3	MT 227	rs1933437	0,49	0,41	NRF	10	V L H E L F G T D I
FMNL1	NN 170	rs7209538	N.D.	0,00	ARF	10	M T W R A Q T M G L
FMNL1	NN 170	rs7209538	N.D.	0,00	ARF	10	M T W R A Q T T G L
FMNL1	NN 170	rs7209538	N.D.	0,00	ARF	9	R M T W R A Q T M
FMNL1	NN 170	rs7209538	N.D.	0,00	ARF	11	R M T W R A Q T M G L
FMNL1	NN 170	rs7209538	N.D.	0,00	ARF	9	R M T W R A Q T T
FMNL1	NN 170	rs7209538	N.D.	0,00	ARF	11	R M T W R A Q T T G L
FMNL1	SS 336	rs12940312	N.D.	0,13	ARF	10	F G G E H E L P C L
FMNL1	SS 336	rs12940312	N.D.	0,13	ARF	10	F S G E H E L P C L
FMNL1	SS 336	rs12940312	N.D.	0,13	ARF	9	G T F G G E H E L
FMNL1	SS 336	rs12940312	N.D.	0,13	ARF	9	G T F S G E H E L
FMNL1	SS 336	rs12940312	N.D.	0,13	ARF	9	T L W Y I Q W R T
FMNL1	SS 336	rs12940312	N.D.	0,13	ARF	9	T L W Y I R W R T
FMNL1	RP 511	rs4792898	N.D.	0,38	NRF	9	I L P V A V A T R
FMNL1	RP 511	rs4792898	N.D.	0,38	ARF	10	Q L G A A V M L R L
FMNL1	RP 511	rs4792898	N.D.	0,38	ARF	10	Q L R A A V M L R L
FMNL1	RP 511	rs4792898	N.D.	0,38	ARF	9	S L W Q L G A A V
FMNL1	RP 511	rs4792898	N.D.	0,38	ARF	10	S L W Q L G A A V M
FMNL1	RP 511	rs4792898	N.D.	0,38	ARF	11	S L W Q L G A A V M L
FMNL1	RP 511	rs4792898	N.D.	0,38	ARF	9	S L W Q L R A A V

FMNL1	RP 511	rs4792898	N.D.	0,38	ARF	10	S L W Q L R A A V M
FMNL1	RP 511	rs4792898	N.D.	0,38	ARF	11	S L W Q L R A A V M L
FMNL1	RP 511	rs4792898	N.D.	0,38	ARF	9	W Q L G A A V M L
FMNL1	RP 511	rs4792898	N.D.	0,38	ARF	9	W Q L R A A V M L
FMNL1	EK 667	rs11555736	N.D.	0,00	NRF	11	E L N D E E V L Q E L
FMNL1	EK 667	rs11555736	N.D.	0,00	NRF	11	E L N D E K V L Q E L
FMNL1	EK 667	rs11555736	N.D.	0,00	NRF	10	L N D E E V L Q E L
FMNL1	EK 667	rs11555736	N.D.	0,00	NRF	10	L N D E K V L Q E L
FMNL1	EK 667	rs11555736	N.D.	0,00	NRF	11	T V F T E L N D E E V
FMNL1	EK 667	rs11555736	N.D.	0,00	NRF	11	T V F T E L N D E K V
FNBP1	SS 136	rs17519205	0,15	0,08	ARF	9	L A G S S L N L V
FNBP1	SS 136	rs17519205	0,15	0,08	ARF	9	L A G S S L N P V
FNBP1	SS 136	rs17519205	0,15	0,08	ARF	9	L V K G D L N A I
FNBP1	SS 136	rs17519205	0,15	0,08	ARF	9	N L V K G D L N A
FNBP1	SS 136	rs17519205	0,15	0,08	ARF	10	N L V K G D L N A I
FNBP1	SS 136	rs17519205	0,15	0,08	ARF	11	N L V K G D L N A I A
FNBP1	SS 136	rs17519205	0,15	0,08	ARF	10	R L A G S S L N L V
FNBP1	SS 136	rs17519205	0,15	0,08	ARF	10	R L A G S S L N P V
FNBP1	SS 136	rs17519205	0,15	0,08	ARF	9	R L A G S S L N L
FNBP1	SS 136	rs17519205	0,15	0,08	ARF	11	S R L A G S S L N L V
FNBP1	SS 136	rs17519205	0,15	0,08	ARF	11	S R L A G S S L N P V
FOSB	SG 33	rs28381241	0,01	N.D.	ARF	11	G L L Q Q S T H R R G
FOSB	SG 33	rs28381241	0,01	N.D.	ARF	11	G L L R Q S T H R R G
FOSB	SG 33	rs28381241	0,01	N.D.	ARF	11	L L Q Q S T H R R G L
FOSB	SG 33	rs28381241	0,01	N.D.	ARF	11	L L R Q S T H R R G L
FOSB	AA 39	rs2282695	0,48	0,27	ARF	11	G L L R Q S T H R R G
FOSB	AA 39	rs2282695	0,48	0,27	ARF	11	G L L R Q S T H R R R
FOSB	AA 39	rs2282695	0,48	0,27	ARF	11	L L R Q S T H R R G L
FOSB	AA 39	rs2282695	0,48	0,27	ARF	11	L L R Q S T H R R R L
GATA2	TA 164	rs2335052	0,35	0,15	ARF	10	G L P H P Y S N P L
GATA2	TA 164	rs2335052	0,35	0,15	ARF	10	G L P H P Y S S P L
GATA2	TA 164	rs2335052	0,35	0,15	ARF	10	N P L W I P P F R L
GATA2	TA 164	rs2335052	0,35	0,15	NRF	11	S L T P T A A H S G S
GATA2	TA 164	rs2335052	0,35	0,15	NRF	11	S L T P T A T H S G S
GATA2	TA 164	rs2335052	0,35	0,15	ARF	10	S P L W I P P F R L
GATA2	GG 480	rs1126560	N.D.	0,00	NRF	10	G L G N R W T S R T
GATA2	GG 480	rs1126560	N.D.	0,00	NRF	9	V Q H G D R H G L
GATA2	GG 480	rs1126560	N.D.	0,00	NRF	9	V Q H G D R H G V
GNA15	IM 187	rs2230330	0,04	0,01	ARF	10	A A A C P P L A S T
GNA15	IM 187	rs2230330	0,04	0,01	ARF	10	A A A Y P P L A S T
GNA15	IM 187	rs2230330	0,04	0,01	ARF	9	A A C P P L A S T
GNA15	IM 187	rs2230330	0,04	0,01	ARF	9	A A Y P P L A S T
GNA15	IM 187	rs2230330	0,04	0,01	NRF	9	V L R S R I P T T
GNA15	IM 187	rs2230330	0,04	0,01	NRF	11	V L R S R I P T T G I
GNA15	IM 187	rs2230330	0,04	0,01	NRF	11	V L R S R M P T T G I
GNA15	LL 232	rs2074865	0,45	0,09	ARF	9	R D R P H L P S L
GNA15	LL 339	rs1637656	0.26	0,21	ARF	10	R T I P T P F Q P L
GNA15	LL 339	rs1637656	0.26	0,21	ARF	10	R T I P T P L Q P L
GNA15	LL 339	rs1637656	0.26	0,21	ARF	9	T I P T P F Q P L
GNA15	LL 339	rs1637656	0.26	0,21	ARF	9	T I P T P L Q P L
HSPA6	GV 96	rs428614	N.D.	0,00	ARF	9	F M T S S W G G A
HSPA6	GV 96	rs428614	N.D.	0,00	ARF	11	F M T S S W G G A P L
HSPA6	GV 96	rs428614	N.D.	0,00	ARF	9	F M T S S W W G A
HSPA6	GV 96	rs428614	N.D.	0,00	ARF	11	F M T S S W W G A P L
HSPA6	AA 216	rs391145	0,412	N.D	ARF	9	F S P L T L V S L
HSPA6	AA 216	rs391145	0,412	N.D	ARF	10	R F S P L T L V S L
HSPA6	AA 216	rs391145	0,412	N.D	ARF	11	R F S P L T L V S L V
HSPA6	AA 216	rs391145	0,412	N.D	ARF	10	S M C R F S P L T L
HSPA6	AA 216	rs391145	0,412	N.D	ARF	11	S M C R F S P L T L V
HSPA6	AA 216	rs391145	0,412	N.D	ARF	11	S M C R F S P L T P V
HSPA6	LP 276	rs393027	0,42	0,49	NRF	11	L S S S T Q A T L E I
HSPA6	LP 276	rs393027	0,42	0,49	NRF	11	R T L S S S T Q A T L
HSPA6	LP 276	rs393027	0,42	0,49	NRF	11	R T P S S S T Q A T L
HSPA6	LP 276	rs393027	0,42	0,49	NRF	11	T L S S S T Q A T L E
HSPA6	LP 276	rs393027	0,42	0,49	NRF	10	T L S S S T Q A T L
HSPA6	KT 297	rs41297718	0,02	0,06	NRF	10	F E G V D F Y K S I
HSPA6	KT 297	rs41297718	0,02	0,06	NRF	10	F E G V D F Y T S I
HSPA6	KT 297	rs41297718	0,02	0,06	ARF	11	G L L H V H H S C P L
HSPA6	KT 297	rs41297718	0,02	0,06	ARF	11	G L L Q V H H S C P L
HSPA6	KT 297	rs41297718	0,02	0,06	ARF	10	L L H V H H S C P L
HSPA6	KT 297	rs41297718	0,02	0,06	ARF	10	L L Q V H H S C P L
HSPA6	KT 297	rs41297718	0,02	0,06	ARF	9	L Q V H H S C P L
HSPA6	KT 297	rs41297718	0,02	0,06	NRF	10	S L F E G V D F Y K
HSPA6	KT 297	rs41297718	0,02	0,06	NRF	10	S L F E G V D F Y T
HSPA6	KT 297	rs41297718	0,02	0,06	ARF	9	W T S T R P S L V
HSPA6	KT 297	rs41297718	0,02	0,06	ARF	9	W T S T S P S L V
HSPA6	VG 447	rs450363	0,47	0,00	NRF	11	F I Q V Y E G E R A M
HSPA6	VG 447	rs450363	0,47	0,00	NRF	10	F I Q V Y E V E R A
HSPA6	VG 447	rs450363	0,47	0,00	NRF	11	F I Q V Y E V E R A M
HSPA6	VG 447	rs450363	0,47	0,00	NRF	9	G V F I Q V Y E V
HSPA6	VG 447	rs450363	0,47	0,00	NRF	10	Q V Y E G E R A M T
HSPA6	VG 447	rs450363	0,47	0,00	NRF	10	Q V Y E V E R A M T
HSPA6	SI 464	rs388218	0,08	N.D.	ARF	9	F E L I G I P P A
HSPA6	SI 464	rs388218	0,08	N.D.	ARF	9	F E L S G I P P A
HSPA6	SI 464	rs388218	0,08	N.D.	ARF	10	L L G R F E L I G I
HSPA6	SI 464	rs388218	0,08	N.D.	ARF	10	L L G R F E L S G I
HSPA6	SI 464	rs388218	0,08	N.D.	ARF	11	N L L G R F E L I G I
HSPA6	SI 464	rs388218	0,08	N.D.	ARF	11	N L L G R F E L S G I

HSPA6	SI 464	rs388218	0,08	N.D.	ARF	9	NLLGRFELI
HSPA6	SI 464	rs388218	0,08	N.D.	NRF	9	SLASLLPHV
HSPA6	SI 464	rs388218	0,08	N.D.	NRF	9	VLNSLASLL
HSPA6	SI 464	rs388218	0,08	N.D.	NRF	9	VLNSVASLL
HSPA6	HR 471	rs41299256	0,06	N.D.	NRF	9	GIPPAPHGV
HSPA6	HR 471	rs41299256	0,06	N.D.	NRF	9	GIPPAPRGV
HSPA6	HR 471	rs41299256	0,06	N.D.	ARF	9	SVASLLPHV
HSPA6	VM 527	rs452004	0,04	N.D.	ARF	10	KIPEEDRRKV
HSPA6	VM 527	rs452004	0,04	N.D.	NRF	9	KMQDKCREV
HSPA6	VM 527	rs452004	0,04	N.D.	NRF	10	KMQDKCREVL
HSPA6	VM 527	rs452004	0,04	N.D.	NRF	9	KVQDKCREV
HSPA6	VM 527	rs452004	0,04	N.D.	NRF	10	KVQDKCREVL
HSPA6	EK 528	rs570189	0,02	N.D.	NRF	11	RMVHEAEQYEA
HSPA6	ED 562	rs753856	0,24	N.D.	NRF	11	SLQEESLRDKI
HSPA6	ED 562	rs753856	0,24	N.D.	NRF	10	SLQEESLREK
HSPA6	ED 562	rs753856	0,24	N.D.	NRF	9	SLQEESLREKI
HSPA6	ED 562	rs753856	0,24	N.D.	NRF	9	SLRDKIPEE
HSPA6	ED 562	rs753856	0,24	N.D.	NRF	9	SLREKIPEE
HSPA6	QR 577	rs368844	0,49	0,14	NRF	9	KCQEVLAWL
HSPA6	QR 577	rs368844	0,49	0,14	NRF	9	KCREVLAWL
HSPA6	QR 577	rs368844	0,49	0,14	NRF	9	KMQDKCQEV
HSPA6	QR 577	rs368844	0,49	0,14	NRF	10	KMQDKCQEVL
ICAM3	AA 2	rs281414	0,02	0,21	ARF	10	ALCQNGHHGT
ICAM3	AA 2	rs281414	0,02	0,21	ARF	11	ALCQNGHHGTI
ICAM3	AA 2	rs281414	0,02	0,21	ARF	10	ALCQNGYHGT
ICAM3	AA 2	rs281414	0,02	0,21	ARF	11	ALCQNGYHGTI
ICAM3	AA 2	rs281414	0,02	0,21	ARF	9	SLSEWLPWY
ICAM3	AA 2	rs281414	0,02	0,21	ARF	10	SLSEWLPWYH
ICAM3	AA 2	rs281414	0,02	0,21	ARF	10	SLSEWPPWYH
ICAM3	VI 63	rs17697947	0,044	0,00	NRF	11	IALETSLSKEL
ICAM3	VI 63	rs17697947	0,044	0,00	NRF	11	VALETSLSKEL
ICAM3	SS 103	rs2304240	0,27	N.D.	ARF	9	LQWLPDNRL
ICAM3	SS 103	rs2304240	0,27	N.D.	ARF	10	LQWLPDNRLL
ICAM3	SS 103	rs2304240	0,27	N.D.	ARF	9	LQWLSDNRL
ICAM3	SS 103	rs2304240	0,27	N.D.	ARF	10	LQWLSDNRLL
ICAM3	SS 103	rs2304240	0,27	N.D.	ARF	11	SVLQWLPDNRL
ICAM3	SS 103	rs2304240	0,27	N.D.	ARF	11	SVLQWLSDNRL
ICAM3	SS 103	rs2304240	0,27	N.D.	ARF	10	VLQWLPDNRL
ICAM3	SS 103	rs2304240	0,27	N.D.	ARF	10	VLQWLSDNRL
ICAM3	GR 115	rs7258015	0,26	N.D	NRF	11	GLPERVELAPL
ICAM3	GR 115	rs7258015	0,26	N.D	NRF	9	GLPERVELA
ICAM3	GR 115	rs7258015	0,26	N.D	NRF	10	ITVYGLPERV
ICAM3	GR 115	rs7258015	0,26	N.D	NRF	10	ITVYRLPERV
ICAM3	GR 115	rs7258015	0,26	N.D	NRF	9	RLPERVELA
ICAM3	GR 115	rs7258015	0,26	N.D	NRF	11	SLPERVELAPL
ICAM3	GR 115	rs7258015	0,26	N.D	NRF	9	SSNITVYGL
ICAM3	GR 115	rs7258015	0,26	N.D	NRF	9	TVYGLPERV
ICAM3	GR 115	rs7258015	0,26	N.D	NRF	9	TVYRLPERV
ICAM3	GR 115	rs7258015	0,26	N.D	NRF	9	YGLPERVEL
ICAM3	GR 115	rs7258015	0,26	N.D	NRF	9	YRLPERVEL
ICAM3	GD 143	rs2304237	0,29	0,00	ARF	9	SPCAAKWRV
ICAM3	GD 143	rs2304237	0,29	0,00	ARF	10	TSPCAAKWRV
ICAM3	ST 525	rs2230399	0,19	0,13	NRF	11	HVREESTYLPL
ICAM3	ST 525	rs2230399	0,19	0,13	NRF	11	HVREETTYLPL
ICAM3	ST 525	rs2230399	0,19	0,13	ARF	9	TMLGRRAPI
ICAM3	ST 525	rs2230399	0,19	0,13	ARF	9	TMLGRRPPI
IL2RG	EK 109	rs17875899	0,02	0,00	NRF	10	YLFSEEITSG
IL2RG	EK 109	rs17875899	0,02	0,00	NRF	10	YLFSKEITSG
IQGAP2	LL 154	rs10036913	0,46	0,44	ARF	9	ILHSRTEFI
IQGAP2	LL 154	rs10036913	0,46	0,44	ARF	11	ILHSRTEFISV
IQGAP2	LL 154	rs10036913	0,46	0,44	ARF	9	ILHSRTEFV
IQGAP2	LL 154	rs10036913	0,46	0,44	ARF	11	ILHSRTEFVSV
IQGAP2	AA 524	rs2431351	0,5	0,42	ARF	9	FGWMRYSKL
IQGAP2	AA 524	rs2431351	0,499	0,42	ARF	9	KLSMRPTWT
IQGAP2	DE 527	rs2431352	0,14	N.D.	NRF	11	EIQQAVDDANV
IQGAP2	DE 527	rs2431352	0,14	N.D.	NRF	11	EIQQAVDEANV
IQGAP2	DE 527	rs2431352	0,14	N.D.	NRF	10	IQQAVDDANV
IQGAP2	DE 527	rs2431352	0,14	N.D.	NRF	10	IQQAVDEANV
IQGAP2	DE 527	rs2431352	0,14	N.D.	NRF	9	QQAVDDANV
IQGAP2	DE 527	rs2431352	0,14	N.D.	NRF	9	QQAVDEANV
IQGAP2	DE 527	rs2431352	0,14	N.D.	ARF	9	SMMPTWTRT
IQGAP2	DD 597	rs2910819	0,5	0,40	ARF	11	CLVMVHGSNST
IQGAP2	DD 597	rs2910819	0,5	0,40	ARF	11	CLVTVHGSNST
IQGAP2	DD 597	rs2910819	0,5	0,40	ARF	10	LVMVHGSNST
IQGAP2	DD 597	rs2910819	0,5	0,40	ARF	9	VMVHGSNST
IQGAP2	FL 629	rs2455230	0,5	0,40	NRF	9	SCFYKESWL
IQGAP2	FL 629	rs2455230	0,5	0,40	NRF	9	SCLYKESWL
IQGAP2	VI 724	rs2431363	0,48	0,42	NRF	9	FIDNTDSIV
IQGAP2	VI 724	rs2431363	0,48	0,42	NRF	11	FIDNTDSIVKI
IQGAP2	VI 724	rs2431363	0,48	0,42	NRF	9	FIDNTDSVV
IQGAP2	VI 724	rs2431363	0,48	0,42	NRF	11	FIDNTDSVVKI
IQGAP2	VI 724	rs2431363	0,48	0,42	NRF	11	SIVKIQSWFRM
IQGAP2	VI 724	rs2431363	0,48	0,42	NRF	11	SVVKIQSWFRM
IQGAP2	WR 1379	rs17681908	0,04	0,00	NRF	9	KIQRNLRTL
IQGAP2	WR 1379	rs17681908	0,04	0,00	NRF	9	KIQRNLWTL
IQGAP2	WR 1379	rs17681908	0,04	0,00	NRF	11	NLRTLEQTGHV
IQGAP2	WR 1379	rs17681908	0,04	0,00	NRF	11	NLWTLEQTGHV
IQGAP2	WR 1379	rs17681908	0,04	0,00	NRF	9	RTLEQTGHV

IQGAP2	WR 1379	rs17681908	0,04	0,00	NRF	9	WTLEQTGHV
ITGAL	RT 791	rs2230433	0,49	0,22	NRF	11	RALRLTAFASL
ITGAL	RT 791	rs2230433	0,49	0,22	NRF	11	TALRLTAFASL
ITGAL	PP 1152	rs11574950	0,05	N.D	ARF	9	HLGKRLGIL
ITGAM	RH 77	rs1143679	0,08	0,15	NRF	9	HLQVPVEAV
ITGAM	RH 77	rs1143679	0,08	0,15	NRF	11	HLQVPVEAVNM
ITGAM	RH 77	rs1143679	0,08	0,15	NRF	10	IHLQVPVEAV
ITGAM	RH 77	rs1143679	0,08	0,15	NRF	10	IRLQVPVEAV
ITGAM	RH 77	rs1143679	0,08	0,15	NRF	9	RLQVPVEAV
ITGAM	RH 77	rs1143679	0,08	0,15	NRF	11	RLQVPVEAVNM
ITGAM	RS 343	rs3087444	0,02	0,00	ARF	11	EVAAPLSMRCL
ITGAM	RS 343	rs3087444	0,02	0,00	ARF	11	RVLRQEVAAPL
ITGAM	RS 343	rs3087444	0,02	0,00	ARF	11	RVLRQEVEAPL
ITGAM	RS 343	rs3087444	0,02	0,00	ARF	10	VAAPLSMRCL
ITGAM	RS 343	rs3087444	0,02	0,00	ARF	10	VLRQEVAAPL
ITGAM	RS 343	rs3087444	0,02	0,00	ARF	10	VLRQEVEAPL
ITGAM	MT 441	rs11861251	0,2	0,16	NRF	9	AMFRQNTGM
ITGAM	MT 441	rs11861251	0,2	0,16	NRF	9	AMFRQNTGT
ITGAM	MT 441	rs11861251	0,2	0,16	NRF	9	GMWESNANV
ITGAM	MT 441	rs11861251	0,2	0,16	NRF	9	GTWESNANV
ITGAM	SP 1146	rs1143678	0,42	0,17	NRF	10	MMSEGGPPGA
ITGAM	SP 1146	rs1143678	0,42	0,17	NRF	10	MMSEGGSPGA
ITGB2	GG 273	rs2230528	0,25	N.D	ARF	10	SISRATESWA
ITGB2	GG 273	rs2230528	0,25	N.D	ARF	10	SISRATGSWA
ITGB2	VV 367	rs2230529	0,23	0,23	ARF	11	TINSPPGSSWI
ITGB2	VV 367	rs2230529	0,23	0,23	ARF	11	TINSPPGYSWI
ITGB2	WR 586	rs5030672	N.D.	0,01	NRF	10	TEGCLNPWRV
KCNAB2	EK 88	rs2229003	0,5	N.D.	ARF	10	GINLFDTAEV
KCNAB2	EK 88	rs2229003	0,5	N.D.	ARF	10	GINLFDTAKV
KCNAB2	EK 88	rs2229003	0,5	N.D.	ARF	9	INLFDTAEV
KCNAB2	EK 88	rs2229003	0,5	N.D.	ARF	9	INLFDTAKV
KCNAB2	EK 88	rs2229003	0,5	N.D.	ARF	10	KVYAAGKAEV
KCNAB2	EK 88	rs2229003	0,5	N.D.	ARF	10	NLFDTAEVYA
KCNAB2	EK 88	rs2229003	0,5	N.D.	ARF	11	NLFDTAEVYAA
KCNAB2	EK 88	rs2229003	0,5	N.D.	ARF	10	NLFDTAKVYA
KCNAB2	EK 88	rs2229003	0,5	N.D.	ARF	11	NLFDTAKVYAA
KCNAB2	AA 92	rs2229004	0,14	0,00	ARF	9	QLARLKWYW
KCNAB2	AA 92	rs2229004	0,14	0,00	ARF	11	SIQQKSTQLAR
KCNAB2	AA 92	rs2229004	0,14	0,00	ARF	9	SIQQKSTQL
KCNAB2	AA 92	rs2229004	0,14	0,00	ARF	11	SSIQQKSTQLA
KCNAB2	GG 122	rs2229005	0,05	0,00	ARF	10	LLGRKGGDGA
KCNAB2	GG 122	rs2229005	0,05	0,00	ARF	10	LLGWKGGDGA
KCNAB2	SS 344	rs2229002	0,49	0,35	ARF	9	HLPLSTRLI
KCNAB2	SS 344	rs2229002	0,49	0,35	ARF	10	HLPLSTRLIV
KCNAB2	SS 344	rs2229002	0,49	0,35	ARF	9	RLPLSTRLI
KCNAB2	SS 344	rs2229002	0,49	0,35	ARF	10	RLPLSTRLIV
LCP2	QQ 326	rs315717	0,49	0,48	ARF	10	MMKMMCIKDL
LCP2	QQ 326	rs315717	0,49	0,48	ARF	10	MMKMMCIRDL
LRMP	GE 25	rs1129442	N.D.	0,00	NRF	10	LLQSREYSSL
LRMP	GE 25	rs1129442	N.D.	0,00	NRF	10	LLQSRGYSSL
LRMP	GE 25	rs1129442	N.D.	0,00	NRF	9	LQSREYSSL
LRMP	GE 25	rs1129442	N.D.	0,00	NRF	9	LQSRGYSSL
LRMP	GE 25	rs1129442	N.D.	0,00	NRF	11	SLLQSREYSSL
LRMP	GE 25	rs1129442	N.D.	0,00	NRF	11	SLLQSRGYSSL
LRMP	SC 197	rs1908946	0,5	N.D.	NRF	9	NCLKLLESL
LRMP	SC 197	rs1908946	0,5	N.D.	NRF	10	NLKKEITNCL
LRMP	SC 197	rs1908946	0,5	N.D.	NRF	10	NLKKEITNSL
LRMP	SC 197	rs1908946	0,5	N.D.	NRF	9	NSLKLLESL
LRMP	SC 197	rs1908946	0,5	N.D.	NRF	11	SLKLLESLTPI
LRMP	FS 322	rs1129443	N.D.	0,00	NRF	9	FLPRNIGNA
LRMP	FS 322	rs1129443	N.D.	0,00	NRF	11	FLPRNIGNAGM
LRMP	FS 322	rs1129443	N.D.	0,00	NRF	9	LRRVTIASL
LRMP	FS 322	rs1129443	N.D.	0,00	NRF	11	SSLRRVTIAFL
LRMP	FS 322	rs1129443	N.D.	0,00	NRF	11	SSLRRVTIASL
LRMP	FS 322	rs1129443	N.D.	0,00	NRF	9	SLPRNIGNA
LRMP	FS 322	rs1129443	N.D.	0,00	NRF	11	SLPRNIGNAGM
LRMP	FS 322	rs1129443	N.D.	0,00	NRF	10	SLRRVTIAFL
LRMP	FS 322	rs1129443	N.D.	0,00	NRF	10	SLRRVTIASL
LRMP	FS 322	rs1129443	N.D.	0,00	NRF	9	TIASLPRNI
LTB	GA 69	rs4647186	0,01	0,04	NRF	9	DLRPGLPAA
LTB	GA 69	rs4647186	0,01	0,04	ARF	10	KQISAPGSQL
LTB	GA 69	rs4647186	0,01	0,04	ARF	10	KQISGPGSQL
LTB	GA 69	rs4647186	0,01	0,04	ARF	9	QISAPGSQL
LTB	GA 69	rs4647186	0,01	0,04	ARF	9	QISGPGSQL
LTB	RS 84	rs4647186	0,01	0,04	NRF	9	DLSPGLPAA
LTB	TT 117	rs2229698	0,4	0,00	ARF	9	GVSDERDAV
LTB	TT 117	rs2229698	0,4	0,00	ARF	10	GVSDERDAVL
LTB	TT 117	rs2229698	0,4	0,00	ARF	9	GVSDERDSV
LTB	TT 117	rs2229698	0,4	0,00	ARF	10	GVSDERDSVL
LTB	DA 122	rs2229699	0,17	N.D.	NRF	11	FLTSGTQFSDA
LTB	DA 122	rs2229699	0,17	N.D.	NRF	9	TQFSDAEGL
LTB	DA 122	rs2229699	0,17	N.D.	NRF	9	TQFSDDEGL
LTB	FF 231	rs4248165	0,01	0,01	ARF	9	GLCEREDLL
LTB	FF 231	rs4248165	0,01	0,01	ARF	9	GLREREDLL
LTB	FF 231	rs4248165	0,01	0,01	ARF	9	SVTPIWWTL
LTB	FF 231	rs4248165	0,01	0,01	ARF	9	TLREGRPSL
LTB	FF 231	rs4248165	0,01	0,01	ARF	10	WTLREGRPSL
LYN	KK 233	rs2227980	0,22	0,03	ARF	10	VLVPSHRSHGI

LYN	KK 233	rs2227980	0,22	0,03	ARF	11	V L V P S H R S H G I
LYN	II 358	rs7001291	N.D.	0,00	ARF	9	R L Q R E W H T L
LYN	II 358	rs7001291	N.D.	0,00	ARF	9	T L S G R T T F T
LYN	II 379	rs1050875	N.D.	0,00	ARF	11	L M F W S P S H S C A
LYN	II 379	rs1050875	N.D.	0,00	ARF	10	S C A K L Q I L A L
MAP4K1	MT 204	rs17847696	N.D.	0,00	NRF	10	D I W S L G I M A I
MAP4K1	MT 204	rs17847696	N.D.	0,00	NRF	10	D I W S L G I T A I
MAP4K1	MT 204	rs17847696	N.D.	0,00	NRF	10	G I M A I E L A E L
MAP4K1	MT 204	rs17847696	N.D.	0,00	NRF	10	G I T A I E L A E L
MAP4K1	MT 204	rs17847696	N.D.	0,00	NRF	9	I M A I E L A E L
MAP4K1	MT 204	rs17847696	N.D.	0,00	NRF	9	I T A I E L A E L
MAP4K1	MT 204	rs17847696	N.D.	0,00	NRF	9	S L G I M A I E L
MAP4K1	MT 204	rs17847696	N.D.	0,00	NRF	10	S L G I M A I E L A
MAP4K1	MT 204	rs17847696	N.D.	0,00	NRF	9	S L G I T A I E L
MAP4K1	MT 204	rs17847696	N.D.	0,00	NRF	10	S L G I T A I E L A
MCM5	ST 180	rs2307340	0,08	0,17	ARF	11	A A A A A T P S A T L
MCM5	ST 180	rs2307340	0,08	0,17	ARF	10	A A A A T P S A T L
MCM5	ST 180	rs2307340	0,08	0,17	ARF	10	A A A A T P S P T L
MCM5	ST 180	rs2307340	0,08	0,17	ARF	9	A A A T P S A T L
MCM5	ST 180	rs2307340	0,08	0,17	ARF	9	A A A T P S P T L
MCM5	ST 180	rs2307340	0,08	0,17	NRF	10	L T N I A M R P G L
MCM5	ST 180	rs2307340	0,08	0,17	NRF	11	T L S N I A M R P G L
MCM5	ST 180	rs2307340	0,08	0,17	NRF	11	T L T N I A M R P G L
MCM5	LL 188	rs2230932	0,04	N.D.	ARF	10	T L P C A L A L R A
MCM5	LL 188	rs2230932	0,04	N.D.	ARF	10	T L P C A L A S R A
MCM5	VI 258	rs2230933	0,03	0,00	NRF	10	K V I P G N R V T I
MCM5	VI 258	rs2230933	0,03	0,00	NRF	10	K V V P G N R V T I
MCM5	VI 258	rs2230933	0,03	0,00	NRF	9	V I P G N R V T I
MCM5	VI 258	rs2230933	0,03	0,00	NRF	9	V V P G N R V T I
MCM5	VI 258	rs2230933	0,03	0,00	NRF	9	Y L C D K V I P G
MCM5	VI 258	rs2230933	0,03	0,00	NRF	10	Y L C D K V I P G N
MCM5	VI 258	rs2230933	0,03	0,00	NRF	9	Y L C D K V V P G
MCM5	VI 258	rs2230933	0,03	0,00	NRF	10	Y L C D K V V P G N
MCM5	KK 569	rs133427	0,19	N.D	ARF	10	L L P S E M W P P A
MCM5	KK 569	rs133427	0,19	N.D	ARF	11	L L P S E M W P P A V
MCM5	KK 569	rs133427	0,19	N.D	ARF	10	L L P S E V W P P A
MCM5	KK 569	rs133427	0,19	N.D	ARF	11	L L P S E V W P P A V
MPL	KN 39	rs17292650	0,04	0,00	ARF	9	G I R L R A P E L
MPL	KN 39	rs17292650	0,04	0,00	ARF	9	G I R L R A P E V
MPL	KN 39	rs17292650	0,04	0,00	ARF	9	R L R A P E L F L
MPL	KN 39	rs17292650	0,04	0,00	ARF	9	R L R A P E V F L
MPL	KN 39	rs17292650	0,04	0,00	NRF	11	S L L A S D S E P L K
MPL	KN 39	rs17292650	0,04	0,00	NRF	11	S L L A S D S E P L N
MPL	PP 70	rs6086	N.D.	0,00	ARF	10	C M P T H G R S P V
MPL	PP 70	rs6086	N.D.	0,00	ARF	10	C M P T R G R S P V
MPL	PP 70	rs6086	N.D.	0,00	ARF	10	C L P A G E A P C L
MPL	PP 70	rs6086	N.D.	0,00	ARF	10	C L P T G E A P C L
MPL	VM 114	rs12731981	0,03	0,03	NRF	10	F P L H L W V K N V
MPL	VM 114	rs12731981	0,03	0,03	NRF	9	H L W V K N M F L
MPL	VM 114	rs12731981	0,03	0,03	NRF	9	H L W V K N V F L
MPL	VM 114	rs12731981	0,03	0,03	NRF	9	N M F L N Q T R T
MPL	VM 114	rs12731981	0,03	0,03	NRF	9	P L H L W V K N V
MPL	VM 114	rs12731981	0,03	0,03	ARF	10	S A A P L G E E C V
MPL	VM 114	rs12731981	0,03	0,03	ARF	10	S A A P L G E E Y V
MPL	EE 230	rs16830693	0,13	0,01	ARF	9	S R P P Q V E K L
MPL	TP 275	rs28928908	N.D.	0,00	NRF	10	G S W G S W S L P V
MPL	TP 275	rs28928908	N.D.	0,00	NRF	10	G S W G S W S L T V
MPL	TP 275	rs28928908	N.D.	0,00	ARF	9	G S W S L T V T V
MPL	TP 275	rs28928908	N.D.	0,00	ARF	9	W L L G I L V P H
N4BP2L1	IL 141	rs1062947	N.D.	0,00	NRF	9	A L E N N Y E V I
N4BP2L1	IL 141	rs1062947	N.D.	0,00	NRF	9	A L E N N Y E V L
N4BP2L1	IL 141	rs1062947	N.D.	0,00	NRF	11	V M A L E N N Y E V I
N4BP2L1	IL 141	rs1062947	N.D.	0,00	NRF	11	V M A L E N N Y E V L
NCF4	SS 23	rs10854695	0,23	0,01	ARF	10	M M L P S Q P T L L
NCF4	SS 23	rs10854695	0,23	0,01	ARF	11	M M L P S Q P T L L T
NCF4	SS 23	rs10854695	0,23	0,01	ARF	10	M M L P S R P T L L
NCF4	SS 23	rs10854695	0,23	0,01	ARF	11	M M L P S R P T L L T
NCF4	SS 23	rs10854695	0,23	0,01	ARF	9	M L P S Q P T L L
NCF4	SS 23	rs10854695	0,23	0,01	ARF	10	M L P S Q P T L L T
NCF4	SS 23	rs10854695	0,23	0,01	ARF	9	M L P S R P T L L
NCF4	SS 23	rs10854695	0,23	0,01	ARF	10	M L P S R P T L L T
NCF4	SS 23	rs10854695	0,23	0,01	ARF	9	M M L P S Q P T L
NCF4	SS 23	rs10854695	0,23	0,01	ARF	9	M M L P S R P T L
NCF4	SS 23	rs10854695	0,23	0,01	ARF	10	R M M L P S Q P T L
NCF4	SS 23	rs10854695	0,23	0,01	ARF	10	R M M L P S R P T L
NCF4	SN 118	rs9610595	N.D.	0,00	NRF	11	A Y M K S L L N L P V
NCF4	SN 118	rs9610595	N.D.	0,00	NRF	9	A Y M K S L L S L
NCF4	SN 118	rs9610595	N.D.	0,00	NRF	11	A Y M K S L L S L P V
NCF4	SN 118	rs9610595	N.D.	0,00	NRF	9	L L N L P V W V L
NCF4	SN 118	rs9610595	N.D.	0,00	NRF	10	L L N L P V W V L M
NCF4	SN 118	rs9610595	N.D.	0,00	NRF	9	L L S L P V W V L
NCF4	SN 118	rs9610595	N.D.	0,00	NRF	10	L L S L P V W V L M
NCF4	SN 118	rs9610595	N.D.	0,00	NRF	10	N A Y M K S L L N L
NCF4	SN 118	rs9610595	N.D.	0,00	NRF	10	N A Y M K S L L S L
NCF4	SN 118	rs9610595	N.D.	0,00	NRF	10	S L L N L P V W V L
NCF4	SN 118	rs9610595	N.D.	0,00	NRF	11	S L L N L P V W V L M
NCF4	SN 118	rs9610595	N.D.	0,00	NRF	10	S L L S L P V W V L
NCF4	SN 118	rs9610595	N.D.	0,00	NRF	11	S L L S L P V W V L M

NCF4	SN 118	rs9610595	N.D.	0,00	NRF	9	SLLNLPVWV
NCF4	SN 118	rs9610595	N.D.	0,00	NRF	9	SLLSLPVWV
NCF4	SN 118	rs9610595	N.D.	0,00	NRF	10	VSLLNLPVWV
NCF4	SN 118	rs9610595	N.D.	0,00	NRF	10	VSLLSLPVWV
NCF4	SN 118	rs9610595	N.D.	0,00	NRF	10	YMKSLLNLPV
NCF4	SN 118	rs9610595	N.D.	0,00	NRF	10	YMKSLLSLPV
NCF4	SS 299	rs11552115	0,26	N.D.	ARF	9	GIWFGCCQM
NCF4	SS 299	rs11552115	0,26	N.D.	ARF	9	GIWFGCCRM
NCF4	EA 304	rs5995361	N.D.	0,00	NRF	10	ALMVRQARGL
NCF4	EA 304	rs5995361	N.D.	0,00	NRF	10	ELMVRQARGL
NCF4	EA 304	rs5995361	N.D.	0,00	NRF	9	LLSDEDVAL
NCF4	EA 304	rs5995361	N.D.	0,00	NRF	10	LLSDEDVALM
NCF4	EA 304	rs5995361	N.D.	0,00	NRF	11	LLSDEDVALMV
NCF4	EA 304	rs5995361	N.D.	0,00	NRF	9	LLSDEDVEL
NCF4	EA 304	rs5995361	N.D.	0,00	NRF	10	LLSDEDVELM
NCF4	EA 304	rs5995361	N.D.	0,00	NRF	11	LLSDEDVELMV
NCF4	EA 304	rs5995361	N.D.	0,00	NRF	10	RLLSDEDVAL
NCF4	EA 304	rs5995361	N.D.	0,00	NRF	11	RLLSDEDVALM
NCF4	EA 304	rs5995361	N.D.	0,00	NRF	10	RLLSDEDVEL
NCF4	EA 304	rs5995361	N.D.	0,00	NRF	11	RLLSDEDVELM
NCF4	FF 320	rs1858	0,11	N.D.	ARF	9	PLPEAPLPL
NCF4	FF 320	rs1858	0,11	N.D.	ARF	9	PLPEAPLSL
NCF4	TT 337	rs28669668	0,09	0,00	ARF	9	QLQGLQHDA
NCF4	TT 337	rs28669668	0,09	0,00	ARF	9	QLQGLQHNA
NCF4	TT 337	rs28669668	0,09	0,00	ARF	9	QCHELTVSL
NCF4	TT 337	rs28669668	0,09	0,00	ARF	9	RCHELTVSL
NUP210	VA 755	rs6795271	0,31	0,38	ARF	10	CLSTPAPSWT
NUP210	VA 755	rs6795271	0,31	0,38	NRF	10	LVPVYTSPQL
NUP210	VA 755	rs6795271	0,31	0,38	ARF	10	RLSTPAPSWT
NUP210	VA 755	rs6795271	0,31	0,38	NRF	11	TLAPVYTSPQL
NUP210	VA 755	rs6795271	0,31	0,38	NRF	11	TLVPVYTSPQL
NUP210	LR 786	rs2280084	0,47	0,39	ARF	11	ATATPCWTWLL
NUP210	LR 786	rs2280084	0,47	0,39	ARF	11	ATATPGWTWLL
NUP210	LR 786	rs2280084	0,47	0,39	ARF	9	ATPCWTWLL
NUP210	LR 786	rs2280084	0,47	0,39	ARF	9	ATPGWTWLL
NUP210	AP 821	rs2280085	0,36	0,16	NRF	10	IEAELPMQLV
NUP210	AP 821	rs2280085	0,36	0,16	NRF	10	IEAELPMQLV
NUP210	AP 821	rs2280085	0,36	0,16	NRF	10	IEPELPMQLV
NUP210	AP 821	rs2280085	0,36	0,16	NRF	10	IEPELPMQLV
NUP210	AP 821	rs2280085	0,36	0,16	NRF	10	SIEAELPMQL
NUP210	AP 821	rs2280085	0,36	0,16	NRF	10	SIEPELPMQL
NUP210	AP 821	rs2280085	0,36	0,16	NRF	11	VLASIEAELPM
NUP210	AP 821	rs2280085	0,36	0,16	NRF	11	VLASIEPELPM
NUP210	AP 821	rs2280085	0,36	0,16	NRF	9	VLASIEAEL
NUP210	AP 821	rs2280085	0,36	0,16	NRF	9	VLASIEPEL
NUP210	PL 966	rs2271503	N.D.	0,00	NRF	10	CLVFLAPAKA
NUP210	PL 966	rs2271503	N.D.	0,00	NRF	10	CLVFPAPAKA
NUP210	PL 966	rs2271503	N.D.	0,00	NRF	11	CLVFPAPAKAV
NUP210	PL 966	rs2271503	N.D.	0,00	NRF	9	DCLVFLAPA
NUP210	PL 966	rs2271503	N.D.	0,00	NRF	9	FLAPAKAVV
NUP210	PL 966	rs2271503	N.D.	0,00	NRF	11	FLAPAKAVVYV
NUP210	PL 966	rs2271503	N.D.	0,00	NRF	11	IMIHDLCLVFL
NUP210	PL 966	rs2271503	N.D.	0,00	NRF	10	LVFLAPAKAV
NUP210	PL 966	rs2271503	N.D.	0,00	NRF	11	LVFLAPAKAVV
NUP210	PL 966	rs2271503	N.D.	0,00	NRF	10	LVFPAPAKAV
NUP210	PL 966	rs2271503	N.D.	0,00	NRF	11	LVFPAPAKAVV
NUP210	PL 966	rs2271503	N.D.	0,00	NRF	10	LAPAKAVVYV
NUP210	PL 966	rs2271503	N.D.	0,00	NRF	11	MIHDLCLVFLA
NUP210	PL 966	rs2271503	N.D.	0,00	NRF	10	MIHDLCLVFL
NUP210	FF 1016	rs2271504	0,47	0,39	NRF	9	ILPLHGPEA
NUP210	FF 1016	rs2271504	0,47	0,39	NRF	9	ILPLYGPEA
NUP210	FF 1016	rs2271504	0,47	0,39	ARF	11	PLHGPEAPSSL
NUP210	FF 1016	rs2271504	0,47	0,39	ARF	11	PLYGPEAPSSL
NUP210	FF 1016	rs2271504	0,47	0,39	NRF	10	QILPLHGPEA
NUP210	FF 1016	rs2271504	0,47	0,39	NRF	10	QILPLYGPEA
NUP210	FF 1016	rs2271504	0,47	0,39	ARF	10	SLPNTSPLWT
NUP210	FF 1016	rs2271504	0,47	0,39	ARF	10	SLPNTSPSWT
NUP210	IM 1096	rs2271505	0,01	0,00	NRF	9	LLIGATIQV
NUP210	IM 1096	rs2271505	0,01	0,00	NRF	10	LLIGATIQVT
NUP210	IM 1096	rs2271505	0,01	0,00	NRF	11	LLIGATIQVTS
NUP210	IM 1096	rs2271505	0,01	0,00	NRF	9	LLIGATMQV
NUP210	IM 1096	rs2271505	0,01	0,00	NRF	10	LLIGATMQVT
NUP210	IM 1096	rs2271505	0,01	0,00	NRF	11	LLIGATMQVTS
NUP210	IM 1096	rs2271505	0,01	0,00	NRF	10	TLLIGATIQV
NUP210	IM 1096	rs2271505	0,01	0,00	NRF	11	TLLIGATIQVT
NUP210	IM 1096	rs2271505	0,01	0,00	NRF	10	TLLIGATMQV
NUP210	IM 1096	rs2271505	0,01	0,00	NRF	11	TLLIGATMQVT
NUP210	IM 1096	rs2271505	0,01	0,00	NRF	9	VTLLIGATI
NUP210	IM 1096	rs2271505	0,01	0,00	NRF	11	VTLLIGATIQV
NUP210	IM 1096	rs2271505	0,01	0,00	NRF	11	VTLLIGATMQV
NUP210	CC 1444	rs2271509	0,5	0,46	ARF	9	ALSAQSAWA
NUP210	CC 1444	rs2271509	0,5	0,46	ARF	9	RAPPTTPVL
NUP210	CC 1444	rs2271509	0,5	0,46	ARF	9	RAPPTTPAL
NUP210	CC 1444	rs2271509	0,5	0,46	ARF	9	VLSAQSAWA
NUP210	SL 1752	rs354479	0,19	N.D	NRF	9	FITYTVGVL
NUP210	SL 1752	rs354479	0,19	N.D	NRF	9	YTVGVLDPA
NUP210	MV 1787	rs354478	0,5	0,46	NRF	10	AIPVTVAFVV
NUP210	MV 1787	rs354478	0,05	0,46	ARF	10	LLWWIAVGPV

PIK3CD	TT 226	rs2230735	0,04	0,00	ARF	11	G L C P A E E G H G V
PIK3CD	TT 226	rs2230735	0,04	0,00	ARF	11	G L C P A E E G H S V
PIK3CD	AA 345	rs28730672	N.D.	0,00	ARF	10	W A F P R Q R D A V
PIK3CD	CR 437	rs28730673	N.D.	0,01	NRF	9	K T G E C C L Y M
PIK3CD	CR 437	rs28730673	N.D.	0,01	NRF	9	K T G E R C L Y M
PIK3CD	CR 437	rs28730673	N.D.	0,01	NRF	9	Q L K T G E C C L
PIK3CD	CR 437	rs28730673	N.D.	0,01	NRF	9	Q L K T G E R C L
PIK3CD	YY 936	rs11121484	0,4	0,06	ARF	11	S V S H S S S P M T L
PIK3CD	YY 936	rs11121484	0,4	0,06	ARF	11	S V S H S S S P T T L
PLCB2	TT 477	rs2229691	0,46	0,23	ARF	9	A P P P P V R I L
PLCB2	TT 477	rs2229691	0,46	0,23	ARF	11	F L A P P P P V R I L
PLCB2	TT 477	rs2229691	0,46	0,23	ARF	9	I L V G R L R A A
PLCB2	TT 477	rs2229691	0,46	0,23	ARF	10	I L V G R L R A A A
PLCB2	TT 477	rs2229691	0,46	0,23	ARF	10	L A P P P P V R I L
PLCB2	TT 477	rs2229691	0,46	0,23	ARF	10	R I L V G R L R A A
PLEK	NK 97	rs3816281	0,5	0,29	NRF	9	D I K K A I K C I
PLEK	NK 97	rs3816281	0,5	0,29	NRF	9	D I N K A I K C I
PLEK	NK 97	rs3816281	0,5	0,29	ARF	10	G I S I R P L N A L
PLEK	NK 97	rs3816281	0,5	0,29	ARF	10	G I S R R P L N A L
PRKCB1	RR 238	rs17847891	N.D.	0,00	ARF	9	R I G Q R Q E T V
PRKCB1	RR 238	rs17847891	N.D.	0,00	ARF	9	R I G Q R Q K T V
PRKCB1	II 508	rs17847876	N.D.	0,00	ARF	10	R L H C P R D N C L
PRKCB1	II 508	rs17847876	N.D.	0,00	ARF	10	R L H R P R D N C L
PRKCB1	PS 563	rs17847879	N.D.	0,00	NRF	9	P M S K E A V A I
PRKCB1	PS 563	rs17847879	N.D.	0,00	NRF	9	S M S K E A V A I
PSD4	PP 58	rs3738908	0,02	0,50	ARF	10	L L N Q P D K M F L
PSD4	PP 58	rs3738908	0,02	0,50	ARF	10	L L N L P D K M F L
PSD4	PP 58	rs3738908	0,02	0,50	ARF	9	L N L P D K M F L
PSD4	PP 58	rs3738908	0,02	0,50	ARF	11	N L P D K M F L P G A
PSD4	PP 58	rs3738908	0,02	0,50	ARF	9	T L L N L P D K M
PSD4	PP 58	rs3738908	0,02	0,50	ARF	11	T L L N L P D K M F L
PSD4	PP 58	rs3738908	0,02	0,50	ARF	9	T L L N Q P D K M
PSD4	PP 58	rs3738908	0,02	0,50	ARF	11	T L L N Q P D K M F L
PSD4	DE 124	rs17857060	N.D.	0,00	ARF	10	A Q A E H S I T R V
PSD4	DE 124	rs17857060	N.D.	0,00	ARF	10	S Q A E H S I T R V
PSD4	SS 216	rs3748914	0,49	N.D	ARF	10	K T Q G K T A A S L
PSD4	SS 216	rs3748914	0,49	N.D	ARF	10	K T Q G K T A V S L
PSD4	AG 269	rs4849167	0,49	0,37	NRF	9	A A S D S H A G V
PSD4	AG 269	rs4849167	0,752	0,37	ARF	10	G L Q T P M Q V G L
PSD4	AG 269	rs4849167	0,49	0,37	NRF	9	G A S D S H A G V
PSD4	AG 269	rs4849167	0,49	0,37	ARF	10	L L E S A F P G G L
PSD4	AG 269	rs4849167	0,49	0,37	ARF	10	L L E S A F P G R L
PSD4	AG 269	rs4849167	0,752	0,37	ARF	10	R L Q T P M Q V G L
PSD4	AG 269	rs4849167	0,49	0,37	NRF	11	S W A A S D S H A G V
PSD4	AG 269	rs4849167	0,49	0,37	ARF	11	V L L E S A F P G G L
PSD4	AG 269	rs4849167	0,49	0,37	ARF	11	V L L E S A F P G R L
PSD4	AG 269	rs4849167	0,49	0,37	NRF	10	W A A S D S H A G V
PSD4	GG 422	rs2241976	0,49	0,38	ARF	9	W A P P G I S S L
PSD4	GG 422	rs2241976	0,49	0,38	ARF	9	W T P P G I S S L
PSMB8	ST 70	rs17220206	0,06	N.D	NRF	9	E M A H G T S T L
PSMB8	ST 70	rs17220206	0,06	N.D	NRF	9	E M A H G T T T L
PSMB8	ST 70	rs17220206	0,06	N.D	NRF	11	Q I E M A H G T S T L
PSMB8	ST 70	rs17220206	0,06	N.D	NRF	11	Q I E M A H G T T T L
PSMB8	ST 70	rs17220206	0,06	N.D	NRF	11	S T L A F K F Q H G V
PSMB8	ST 70	rs17220206	0,06	N.D	NRF	11	T T L A F K F Q H G V
PSMB8	LL 163	rs11540143	N.D.	0,00	ARF	10	P L Y G Q Y D L W L
PSMB10	LL 107	rs20549	0,42	0,32	ARF	9	G V Q D G A T R V
PSMB10	GG 151	rs14178	0,23	0,04	ARF	10	A A L R C A S P W L
PSMB10	GG 151	rs14178	0,23	0,04	ARF	10	A A L R R A S P W L
PSMB10	GG 151	rs14178	0,23	0,04	ARF	9	A L R C A S P W L
PSMB10	GG 151	rs14178	0,23	0,04	ARF	10	A L R C A S P W L L
PSMB10	GG 151	rs14178	0,23	0,04	ARF	9	A L R R A S P W L
PSMB10	GG 151	rs14178	0,23	0,04	ARF	10	A L R R A S P W L L
PTPN22	RR 620	rs2476601	0,25	0,00	NRF	11	P V R T P E S F I V V
PTPN22	RR 620	rs2476601	0,25	0,00	NRF	11	P V W T P E S F I V V
PTPN22	RR 620	rs2476601	0,25	0,00	NRF	9	R T P E S F I V V
PTPN22	RR 620	rs2476601	0,25	0,00	NRF	9	W T P E S F I V V
PTPN6	PL 28	rs11547853	N.D.	0,00	ARF	10	R L G P V A R T R V
PTPN6	PL 28	rs11547853	N.D.	0,00	ARF	11	S R L G P V A R T R V
PTPN6	PL 28	rs11547853	N.D.	0,00	ARF	11	S W L G P V A R T R V
PTPN6	PL 28	rs11547853	N.D.	0,00	ARF	10	S T V A S R L G P V
PTPN6	PL 28	rs11547853	N.D.	0,00	ARF	10	S T V A S W L G P V
PTPN6	PL 28	rs11547853	N.D.	0,00	ARF	9	T V A S R L G P V
PTPN6	PL 28	rs11547853	N.D.	0,00	ARF	9	T V A S W L G P V
PTPN6	PL 28	rs11547853	N.D.	0,00	ARF	10	W L G P V A R T R V
PTPN6	VV 85	rs1048596	N.D.	0,00	ARF	10	T L S S R V C C R T
PTPN6	VV 85	rs1048596	N.D.	0,00	ARF	10	T L S S R V S C R T
PTPN6	VV 85	rs1048596	N.D.	0,00	ARF	10	V L H S A A G C A A
PTPN6	VV 85	rs1048596	N.D.	0,00	ARF	10	V L H S A A G C P A
RASGRP2	II 83	rs11231864	N.D.	0,00	ARF	10	N V P P G Q V L D L
RASGRP2	II 83	rs11231864	N.D.	0,00	ARF	11	Q V L D F R L P S G V
RASGRP2	II 83	rs11231864	N.D.	0,00	ARF	11	Q V L D L R L P S G V
RASGRP2	II 83	rs11231864	N.D.	0,00	ARF	10	V L D F R L P S G V
RASGRP2	II 83	rs11231864	N.D.	0,00	ARF	10	V L D L R L P S G V
RASGRP2	GG 583	rs2230414	0,35	N.D	ARF	9	A L A G E A P G L
RASGRP2	GG 583	rs2230414	0,35	N.D	ARF	9	A L E G E A P G L
RASGRP2	GG 583	rs2230414	0,35	N.D	ARF	9	S L C P A L A G E
RASGRP2	GG 583	rs2230414	0,35	N.D	ARF	10	S L C P A L A G E A

RASGRP2	GG 583	rs2230414	0,35	N.D	ARF	9	S L C P A L E G E
RASGRP2	GG 583	rs2230414	0,35	N.D	ARF	10	S L C P A L E G E A
SELL	GG 22	rs4987279	0,04	0,00	ARF	10	L M E H L Q V V G L
SELL	GG 22	rs4987279	0,04	0,00	ARF	10	L M E H L Q V V G V
SELL	LL 107	rs1051091	0,38	0,17	ARF	11	L L K K Q R T G E M V
SELL	LL 107	rs1051091	0,38	0,17	ARF	9	N L L L K K Q R T
SELL	LL 107	rs1051091	0,38	0,17	ARF	9	N L S L K K Q R T
SELL	LL 107	rs1051091	0,38	0,17	ARF	11	S L K K Q R T G E M V
SELL	FL 193	rs1131498	0,29	0,28	NRF	9	Q L V I Q C E P L
SELL	SP 213	rs2229569	0,26	0,09	NRF	11	E L G T M D C T H P L
SELL	SP 213	rs2229569	0,26	0,09	NRF	11	E L G T M D C T H S L
SELL	SP 213	rs2229569	0,26	0,09	NRF	9	G T M D C T H P L
SELL	SP 213	rs2229569	0,26	0,09	NRF	9	G T M D C T H S L
SELL	SP 213	rs2229569	0,26	0,09	ARF	9	S L F G K L Q L Q
SELL	SP 213	rs2229569	0,26	0,09	ARF	10	S L F G K L Q L Q L
SELPLG	IM 62	rs2228315	0,34	0,07	NRF	10	F L P E T E P P E I
SELPLG	IM 62	rs2228315	0,34	0,07	NRF	11	F L P E T E P P E I L
SELPLG	IM 62	rs2228315	0,34	0,07	NRF	10	F L P E T E P P E M
SELPLG	IM 62	rs2228315	0,34	0,07	NRF	11	F L P E T E P P E M L
SELPLG	IM 62	rs2228315	0,34	0,07	NRF	9	I L R N S T D T T
SELPLG	IM 62	rs2228315	0,34	0,07	NRF	11	I L R N S T D T T P L
SELPLG	IM 62	rs2228315	0,34	0,07	NRF	11	M L R N S T D T T P L
SELPLG	VM 264	rs7300972	0,08	0,00	NRF	11	L S M E P T T K R G L
SELPLG	VM 264	rs7300972	0,08	0,00	NRF	11	L S V E P T T K R G L
SELPLG	VM 264	rs7300972	0,08	0,00	NRF	10	S M E P T T K R G L
SELPLG	VM 264	rs7300972	0,08	0,00	NRF	10	S V E P T T K R G L
SEPT6	PL 406	rs17856302	N.D.	0,40	ARF	11	E L L Q S Q G S Q A G
SEPT6	PL 406	rs17856302	N.D.	0,40	ARF	9	L L Q S Q G S Q A
SEPT6	PL 406	rs17856302	N.D.	0,40	NRF	9	R L S C P S P R A
SEPT6	PL 406	rs17856302	N.D.	0,40	NRF	9	R L S C S S P R A
SF1	PP 499	rs2277308	N.D.	0,00	ARF	11	P M A T T A A A A S A
SF1	PP 499	rs2277308	N.D.	0,00	ARF	11	P V A T T A A A A S A
SF1	PP 499	rs2277308	N.D.	0,00	ARF	9	S L W S S S P M A
SF1	PP 499	rs2277308	N.D.	0,00	ARF	10	S L W S S S P M A T
SF1	PP 499	rs2277308	N.D.	0,00	ARF	11	S L W S S S P M A T T
SF1	PP 499	rs2277308	N.D.	0,00	ARF	9	S L W S S S P V A
SF1	PP 499	rs2277308	N.D.	0,00	ARF	10	S L W S S S P V A T
SF1	PP 499	rs2277308	N.D.	0,00	ARF	11	S L W S S S P V A T T
SOCS2	GE 7	rs7956250	N.D.	0,01	ARF	10	F S H D P A V P W A
SP110	RW 112	rs1129411	N.D.	0,08	NRF	10	W Q S R D T P I L L
SP110	RW 112	rs1129411	N.D.	0,08	NRF	10	Y E W Q S R D T P I
SP110	VA 128	rs11556887	0,11	N.D.	NRF	10	G L A E G S S L H T
SP110	VA 128	rs11556887	0,11	N.D.	NRF	10	G L V E G S S L H T
SP110	VA 128	rs11556887	0,11	N.D.	NRF	10	I L L E A P T G L A
SP110	VA 128	rs11556887	0,11	N.D.	NRF	11	I L L E A P T G L A E
SP110	VA 128	rs11556887	0,11	N.D.	NRF	10	I L L E A P T G L V
SP110	VA 128	rs11556887	0,11	N.D.	NRF	11	I L L E A P T G L V E
SP110	VA 128	rs11556887	0,11	N.D.	NRF	9	L L E A P T G L V
SP110	VA 206	rs28930679	0,05	N.D.	NRF	11	K M N A E E D S E E M
SP110	VA 206	rs28930679	0,05	N.D.	NRF	11	K M N V E E D S E E M
SP110	KE 207	rs9061	0,31	0,11	NRF	11	K M N A K E D S E E M
SP110	RG 299	rs1365776	0,52	0,44	NRF	9	S L P G G T A S S
SP110	RG 299	rs1365776	0,52	0,44	NRF	9	S L P R G T A S S
SP110	LS 303	rs17856587	N.D.	0,00	NRF	9	S L P R G T A S S
SP110	SL 425	rs3948464	0,29	0,10	NRF	11	R L K E K K K E K D I
SP110	MT 523	rs1135791	0,41	0,45	NRF	9	N I R C E G M T L
SP110	MT 523	rs1135791	0,41	0,45	NRF	9	N I R C E G T T L
SP110	CC 577	rs13018234	0,12	N.D.	ARF	10	E L H L L Q D E E V
SP110	IM 579	rs3948463	0,05	0,08	ARF	10	E L H L L Q D K E V
SP110	IM 579	rs3948463	0,05	0,08	ARF	11	H L L Q D E E V F R K
SP110	IM 579	rs3948463	0,05	0,08	ARF	11	H L L Q D K E V F R K
SP110	IM 579	rs3948463	0,05	0,08	NRF	10	M L W S C T F C R I
SP110	IM 579	rs3948463	0,05	0,08	NRF	10	M L W S C T F C R M
SP110	IM 579	rs3948463	0,05	0,08	NRF	11	R M L W S C T F C R I
SP110	IM 579	rs3948463	0,05	0,08	NRF	11	R M L W S C T F C R M
SYNGR1	LF 153	rs1062695	N.D.	0,00	NRF	10	A A I A L S F F S I
SYNGR1	LF 153	rs1062695	N.D.	0,00	NRF	9	A L S F F S I F T
SYNGR1	LF 153	rs1062695	N.D.	0,00	NRF	11	A L S F F S I F T W A
SYNGR1	LF 153	rs1062695	N.D.	0,00	NRF	10	A A I A F S F F S I
SYNGR1	LF 153	rs1062695	N.D.	0,00	NRF	9	A I A F S F F S I
SYNGR1	LF 153	rs1062695	N.D.	0,00	NRF	9	A I A L S F F S I
SYNGR1	LF 153	rs1062695	N.D.	0,00	NRF	10	F S F F S I F T W A
SYNGR1	LF 153	rs1062695	N.D.	0,00	NRF	10	I A F S F F S I F T
SYNGR1	LF 153	rs1062695	N.D.	0,00	NRF	10	I A L S F F S I F T
SYNGR1	LF 153	rs1062695	N.D.	0,00	ARF	9	L L L F L H L H L
SYNGR1	LF 153	rs1062695	N.D.	0,00	ARF	9	P L L F L H L H L
SYNGR1	LF 153	rs1062695	N.D.	0,00	NRF	10	R L L L F L H L H L
SYNGR1	KQ 164	rs11548411	N.D.	0,09	ARF	9	F T W A G K A V L
SYNGR1	KQ 164	rs11548411	N.D.	0,09	ARF	9	F T W A G Q A V L
SYNGR1	KQ 164	rs11548411	N.D.	0,09	ARF	10	G Q G C A G L P A V
SYNGR1	KQ 164	rs11548411	N.D.	0,09	ARF	10	H L G G P G C A G L
SYNGR1	KQ 164	rs11548411	N.D.	0,09	ARF	10	H L G G Q G C A G L
SYNGR1	KQ 164	rs11548411	N.D.	0,09	ARF	9	L G G P G C A G L
SYNGR1	KQ 164	rs11548411	N.D.	0,09	ARF	10	S I F T W A G K A V
SYNGR1	KQ 164	rs11548411	N.D.	0,09	ARF	11	S I F T W A G K A V L
SYNGR1	KQ 164	rs11548411	N.D.	0,09	ARF	10	S I F T W A G Q A V
SYNGR1	KQ 164	rs11548411	N.D.	0,09	ARF	11	S I F T W A G Q A V L
ZFP36L2	II 203	rs8098	0,08	0,08	ARF	10	A V P H L S Y H W L

ZFP36L2	II 203	rs8098	0,08	0,08	ARF	10	H L S Y H R L L P L
ZFP36L2	II 203	rs8098	0,08	0,08	ARF	10	H L S Y H W L L P L
ZFP36L2	II 203	rs8098	0,08	0,08	ARF	9	R L L P L W A A L
ZFP36L2	II 203	rs8098	0,08	0,08	ARF	11	R L L P L W A A L P L
ZFP36L2	II 203	rs8098	0,08	0,08	ARF	9	W L L P L W A A L
ZFP36L2	II 203	rs8098	0,08	0,08	ARF	11	W L L P L W A A L P L
ZFP36L2	LL 254	rs7933	0,5	0,32	ARF	10	A A P Q P Q L L G L
ZFP36L2	LL 254	rs7933	0,5	0,32	ARF	10	G L P A G A A A Q A
ZFP36L2	LL 254	rs7933	0,5	0,32	ARF	10	G L P A G A A A Q V
ZFP36L2	LL 254	rs7933	0,5	0,32	ARF	11	Q V A P Q P Q L L G L
ZFP36L2	LL 254	rs7933	0,5	0,32	ARF	10	V A P Q P Q L L G L
HMHA1 (red)	RH 139	rs1801284	0,04	0,37	NRF	9	V L H D D L L E A
ADIR-F (N.D.)	FL 13	rs2296377	0,28	0,03	ARF	11	S V A P A L A L F P A
CMV (green)	none	no polymorphism	-	N.D.	NRF	9	N L V P M V A T V
EBV (orange)	none	no polymorphism	-	N.D.	NRF	9	G L C T L V A M L
FLU (pink)	none	no polymorphism	-	N.D.	NRF	9	G I L G F V F T L
HY (N.D.)	none	no polymorphism	-	N.D.	NRF	9	F I D S Y I C Q V
Mart-I (blue)	none	no polymorphism	-	N.D.	NRF	9	A A G I G I L T V
A3-GP100 (grey)	none	no polymorphism	-	N.D.	NRF	9	L I Y R R R L M K

$^{\alpha}$ dbSNP average heterozygosity (build126)

$^{\beta}$ SNP frequency obtained from customized SNP genotyping array. Data represents the allele frequency of 100 Dutch individuals

$^{\gamma}$ Peptide sequences are provided with polymorphic residue in red
