# Supplementary material for: High-Throughput Identification of Potential Minor Histocompatibility Antigens by MHC Tetramer-Based Screening: Feasibility and Limitations
Source: PLoS One. 2011 Aug 5;6(8):e22523. doi: 10.1371/journal.pone.0022523 (PMC3151248; doi:10.1371/journal.pone.0022523)
Supplement: Table S3 — Dual-encoding pMHC tetramer scheme. (PDF) [file pone.0022523.s005.pdf]

Supplementary Table III. Dual-encoding pMHC tetramer scheme

Peptide number	Gene symbol	Peptide Sequence [α]	RS MHC-ELISA [β]	RS MHC-Bead [γ]	Tetramer group	Dual-color encoding
1	NUP210	MIHDLCLVFL	71	38	1	PE & Q565
2	NUP210	CLVFPAPAKAV	56	51		PE & Q585
3	PSD4	VLLESAFPGGL	73	79		PE & Q605
4	PLCB2	RILVGRLRAA	84	79		PE & Q655
5	HSPA6	SLASLLPHV	88	101		PE & Q705
6	CSF3R	TQPGPLVPL	70	63		PE & Q800
7	HSPA6	GIPPAPHGV	73	98		APC & Q565
8	ARHGAP15	KLGDCIWTYL	78	39		APC & Q605
9	GNA15	RTIPTPLQPL	74	56		APC & Q655
10	CPVL	ALTEHSLMGM	22	86		APC & Q800
11	ICAM3	ALCQNGYHGTI	61	66		Q565 & Q605
12	PSD4	TLLNQPDKMFL	104	84		Q565 & Q655
13	PSD4	LLNQPDKMFL	82	21		Q565 & Q705
14	SELL	GTMDCTHPL	120	74		Q585 & Q605
15	ZFP36L2	WLLPLWAALPL	81	77		Q585 & Q655
16	MPL	RLRAPEVFL	66	31		Q585 & Q705
17	HSPA6	GLLQVHHSCPL	84	33		Q605 & Q705
18	NCF4	MMLPSQPTLL	86	95		Q605 & Q800
19	NCF4	MLPSQPTLL	50	56		Q655 & Q705
20	MCM5	YLCDKVIPG	96	90		Q655 & Q800
21	HY	FIDSYICQV	N.D.	N.D.		Q705 & Q800
22	CPVL	RQVGDFHQV	79	76	2	PE & APC
23	CD79b	LLLSAEVQQHL	89	97		PE & Q565
24	KCNAB2	NLFDTAKVYA	95	85		PE & Q585
25	HSPA6	NLLGRFELIGI	65	70		PE & Q605
26	HSPA6	VLNSVASLL	107	58		PE & Q655
27	NUP210	DCLVFLAPA	46	57		PE & Q705
28	NUP210	QILPLHGPEA	63	51		PE & Q800
29	ICAM3	VLQWLSDNRL	59	43		APC & Q585
30	ARHGAP 25	SLQRMVQEL	92	96		APC & Q605
31	SP110	ELHLLQDEEV	26	25		APC & Q655
32	NUP210	VLASIEPELPM	70	55		APC & Q705
33	SELPLG	FLPETEPPEM	94	93		APC & Q800
34	ICAM3	TMLGRRPPI	69	88		Q565 & Q605
35	SELL	SLFGKLQLQL	42	60		Q565 & Q655
36	FNBP1	LAGSSLNPV	67	99		Q565 & Q705
37	DOCK2	SIQNYHPFA	60	51		Q585 & Q605
38	ZFP36L2	HLSYHRLLPL	77	44		Q585 & Q655
39	DOCK2	KLLQIQLRAKV	49	78		Q585 & Q705
40	DOCK2	KLLQIQLCA	103	72		Q605 & Q655
41	HSPA6	SLFEGVDFYT	77	77		Q605 & Q705
42	FLT3	SMPQGTFPV	63	87		Q605 & Q800
43	SF1	SLWSSSPMA	101	102		Q655 & Q705
44	CD48	LLFETVMCDT	100	72		Q655 & Q800
45	HSPA6	FMTSSWWRAPL	N.D.	73		Q705 & Q800
46	HMHA1	VLHDDLLEA	83	91	3	PE & APC
47	IQGAP2	SVVKIQSWFRM	59	62		PE & Q565
48	KCNAB2	NLFDTAEVYAA	50	59		PE & Q585
49	ITGAL	TALRLTAFASL	64	34		PE & Q605
50	LCP2	MMKMMCIKDL	36	69		PE & Q655
51	HSPA6	NLLGRFELI	65	76		PE & Q705
52	CSF3R	TQPGPLAPL	76	79		PE & Q800
53	ZFP36L2	GLPAGAAAQA	35	54		APC & Q565
54	DOCK2	TLTTGEWAV	68	73		APC & Q605
55	SP110	MLWSCTFCRM	63	88		APC & Q655
56	ITGAM	MMSEGGPPGA	59	63		APC & Q705
57	NUP210	VLASIEPEL	79	96		APC & Q800
58	ICAM3	ALCQNGYHGT	56	74		Q565 & Q605
59	PSD4	TLLNLPDKMFL	93	93		Q565 & Q655
60	PSD4	LLNLPDKMFL	88	26		Q565 & Q705
61	HSPA6	LLQVHHSCPL	33	-14		Q585 & Q605
62	BTK	YIPSCTVVGM	52	66		Q585 & Q655
63	ZFP36L2	RLLPLWAAL	88	71		Q585 & Q705
64	EVI2B	LPPPPPLLDL	69	19		Q605 & Q800
65	EVI2B	DLPPPPPLL	41	58		Q655 & Q705
66	ARHGAP4	SLCPWSWRAA	90	78		Q655 & Q800
67	ADIR-1F	SVAPALALFPA	N.D.	N.D.		Q705 & Q800
68	CPVL	RQAGDFHQV	81	86	4	PE & APC
69	CD79b	LLLPAEVQQHL	84	99		PE & Q565
70	KCNAB2	NLFDTAEVYA	81	90		PE & Q585
71	PLCB2	FLAPPPPVRIL	70	44		PE & Q605
72	HSPA6	VLNSLASLL	86	49		PE & Q655
73	CSF3R	ITQPGPLVPL	82	75		PE & Q705
74	NUP210	ILPLHGPEA	64	43		PE & Q800
75	ICAM3	VLQWLPDNRL	67	52		APC & Q585
76	ITGAM	GTWESNANV	52	74		APC & Q605
77	GNA15	TIPTPLQPL	67	66		APC & Q655
78	NUP210	VLASIEAELPM	87	57		APC & Q705
79	SELPLG	FLPETEPPEI	96	90		APC & Q800
80	ICAM3	TMLGRRAPI	50	58		Q565 & Q605
81	FNBP1	RLAGSSLNPV	91	104		Q565 & Q655
82	FNBP1	LAGSSLNLV	71	79		Q565 & Q705
83	ZFP36L2	WLLPLWAAL	97	96		Q585 & Q655
84	DOCK2	RLTSTNPTT	67	75		Q585 & Q705
85	NCF4	MMLPSQPTLLT	52	70		Q605 & Q655
86	BTK	CLCLLNPQGT	18	21		Q605 & Q705
87	NCF4	MMLPSRPTL	79	61		Q605 & Q800
88	N4BP2L1	KLYSENLTLA	122	86		Q655 & Q705
89	ITGAM	RLQVPVEAV	58	81	5	PE & APC
90	ARHGAP4	SLLSPLHCWAV	82	71		PE & Q565
91	FLT3	KVLHELFGMDI	84	79		PE & Q585
92	LRMP	SLKLLESLTPI	90	93		PE & Q605
93	HSPA6	LLGRFELIGI	82	54		PE & Q655
94	LRMP	NCLKLLESL	59	51		PE & Q705
95	HSPA6	FIQVYEVERA	77	80		PE & Q800
96	NCF4	RMMLPSQPTL	72	80		APC & Q565
97	NUP210	RAPPTTPAL	65	80		APC & Q585
98	ARHGAP 25	SLQSTVQEL	91	94		APC & Q605
99	SP110	MLWSCTFCRI	100	81		APC & Q655
100	NUP210	VLASIEAEL	86	99		APC & Q800

101	FNBP1	S R L A G S S L N P V	90	90		Q565 & Q655
102	FNBP1	R L A G S S L N L	91	73		Q565 & Q705
103	SP110	I L L E A P T G L V	78	73		Q585 & Q605
104	ZFP36L2	R L L P L W A A L P L	88	64		Q585 & Q655
105	ARHGAP 25	M L L K T S E F L	92	101		Q585 & Q705
106	DOCK2	Q L C A K V P L L	94	82		Q605 & Q655
107	IL2RG	Y L F S K E I T S G	88	76		Q605 & Q705
108	HSPA6	L L H V H H S C P L	61	26		Q605 & Q800
109	FMNL1	S L W Q L G A A V M L	96	95		Q655 & Q705
110	SYNGR1	F T W A G K A V L	100	90		Q655 & Q800
111	CD48	F V S G S G I A I A	N.D.	99		Q705 & Q800
112	CD79b	T M A S T S V S R S A	53	72	6	PE & APC
113	IQGAP2	F I D N T D S V V	49	74		PE & Q565
114	PSD4	V L L E S A F P G R L	72	55		PE & Q585
115	PSD4	R L Q T P M Q V G L	87	65		PE & Q605
116	HSPA6	S V A S L L P H V	88	90		PE & Q655
117	CSF3R	I T Q P G P L A P L	73	83		PE & Q705
118	HSPA6	G I P P A P R G V	71	96		PE & Q800
119	DOCK2	L T L T T G E W A V	59	68		APC & Q585
120	ITGAM	G M W E S N A N V	73	73		APC & Q605
121	CPVL	A L T E R S L M G M	72	23		APC & Q705
122	LTB	F L T S G T Q F S D A	61	68		APC & Q800
123	HSPA6	L I F D L G G G T	52	48		Q565 & Q605
124	FNBP1	R L A G S S L N L V	84	93		Q565 & Q655
125	SELL	G T M D C T H S L	87	57		Q565 & Q705
126	NCF4	A L M V R Q A R G L	74	45		Q585 & Q605
127	CD69	S T L Q G L T S V	74	78		Q585 & Q655
128	DOCK2	R L T S T N P T M	43	50		Q585 & Q705
129	NCF4	M M L P S R P T L L T	45	58		Q605 & Q655
130	NCF4	M M L P S R P T L L	78	93		Q605 & Q705
131	NCF4	M M L P S Q P T L	90	94		Q605 & Q800
132	SYNGR1	F T W A G Q A V L	95	81		Q655 & Q705
133	N4BP2L1	K I Y S E N L K L A	104	82		Q655 & Q800
134	CSF3R	A M A R D P H S L W V	N.D.	104		Q705 & Q800
135	CPVL	R V T S I R L F E V	54	66	7	PE & APC
136	IQGAP2	S I V K I Q S W F R M	55	77		PE & Q565
137	FLT3	V L H E L F G M D I	76	90		PE & Q585
138	ITGAL	R A L R L T A F A S L	92	29		PE & Q605
139	PSD4	L L E S A F P G G L	57	68		PE & Q655
140	PLCB2	I L V G R L R A A	104	86		PE & Q705
141	LRMP	S L L Q S R E Y S S L	74	13		PE & Q800
142	SELL	Q L V I Q C E P L	67	65		APC & Q565
143	NUP210	L L W W I A V G P V	80	80		APC & Q585
144	ARHGAP 25	S L Q S M V Q E L	93	96		APC & Q605
145	BTK	K L A N I Q C P C L	87	77		APC & Q655
146	PSMB10	A L R C A S P W L	65	26		APC & Q705
147	SELPLG	F L P E T E P P E M L	69	60		APC & Q800
148	FNBP1	S R L A G S S L N L V	77	72		Q565 & Q655
149	PSD4	L N L P D K M F L	64	58		Q565 & Q705
150	SP110	I L L E A P T G L A	66	71		Q585 & Q605
151	ZFP36L2	H L S Y H W L L P L	80	25		Q585 & Q655
152	DOCK2	K L L Q I Q L C A K V	53	66		Q585 & Q705
153	DOCK2	K L L Q I Q L R A	83	20		Q605 & Q655
154	IL2RG	Y L F S E E I T S G	88	75		Q605 & Q705
155	HSPA6	V L V E G S T R I	74	55		Q605 & Q800
156	N4BP2L1	K L Y S E N L K L A	108	93		Q655 & Q705
157	CD48	L L S E T V M C D T	100	81		Q655 & Q800
158	CD48	F V S G S G I A T A	110	91	8	PE & APC
159	KCNAB2	M I M A S T S S I	91	92		PE & Q565
160	NUP210	I M I H D L C L A	104	86		PE & Q585
161	NCF4	L L S D E D V A L	92	97		PE & Q605
162	ARHGAP 25	S L Q R T V Q E L	87	84		PE & Q655
163	EVI2B	L L F L L Q M M Q V	77	104		PE & Q705
164	NCF4	R L L S D E D V E L M	67	64		APC & Q565
165	EVI2B	L L Q M M Q V C L	74	91		APC & Q585
166	FMNL1	S L W Q L G A A V M	76	61		APC & Q605
167	CORO1A	K L Q A P V Q E L	79	53		APC & Q655
168	LYN	R L Q R E W H T L	69	54		APC & Q705
169	RASGRP2	V L D F R L P S G V	51	43		APC & Q800
170	PTPN6	S T V A S R L G P V	72	74		Q565 & Q605
171	NCF4	R C H E L T V S L	33	69		Q565 & Q655
172	NCF4	S L L S L P V W V L M	74	48		Q565 & Q705
173	N4BP2L1	M A L E N N Y E V	59	83		Q585 & Q605
174	MAP4K1	S L G I M A I E L	71	70		Q585 & Q655
175	SEPT6	R L S C P S P R A	53	64		Q585 & Q705
176	FCER1A	I S T Q Q Q A T F L L	56	55		Q605 & Q655
177	DUSP22	M L G D P V P T P T	41	88		Q605 & Q705
178	SP110	E L H L L Q D K E V	23	47		Q655 & Q800
179	ATP2A3	K M F V K G A P E S V	N.D.	98		Q705 & Q800
180	N4BP2L1	K I Y S E N L T L A	92	95	9	PE & APC
181	N4BP2L1	T M K I Y S E N L T L	88	83		PE & Q565
182	FMNL1	S L W Q L G A A V	88	91		PE & Q655
183	EVI2B	M M Q V C L H H L	84	65		PE & Q705
184	NUP210	L L I G A T M Q V	88	81		PE & Q800
185	HSPA6	F M T S S W W R A	82	78		APC & Q565
186	IQGAP2	I L H S R T E F I	80	81		APC & Q605
187	LRMP	F L P R N I G N A	62	79		APC & Q655
188	FLT3	A L A R G A G T V P L	76	73		APC & Q705
189	NCF4	P L P E A P L S L	77	91		APC & Q800
190	NCF4	A I S A N I A D I	47	56		Q565 & Q605
191	EVI2B	L L F L L Q M M Q I	65	74		Q565 & Q655
192	DOK2	T L P P R P D H I	54	66		Q565 & Q705
193	NCF4	L L S D E D V E L M V	62	68		Q585 & Q605
194	DOK2	L L A S S M S S Q L	62	59		Q585 & Q655
195	NUP210	I M I H D L C L V	60	54		Q605 & Q655
196	DOK2	Q L G R I S L L L	63	37		Q605 & Q705
197	CSF3R	A Q D P H S L W V	60	64		Q605 & Q800
198	ARHGAP15	F L R A E N E T G N M	39	64		Q655 & Q705
199	FMNL1	Q L R A A V M L R L	65	19		Q655 & Q800
200	CSF3R	A M A Q D P H S L W V	N.D.	103		Q705 & Q800
201	N4BP2L1	K L Y S E N L T L	94	92	10	PE & APC
202	MPL	H L W V K N V F L	109	93		PE & Q565
203	ATP2A3	K M N V F D T N L	89	89		PE & Q585
204	BTK	S L T A I S T T L	83	80		PE & Q705
205	EVI2B	F L L Q M M Q V C L	86	82		APC & Q565

206	SF1	SLWSSSPVAT	69	84		APC & Q585
207	IQGAP2	ILHSRTEFV	77	86		APC & Q605
208	NCF4	SLLNLPVWV	73	92		APC & Q705
209	PIK3CD	SVSHSSSPMTL	76	52		APC & Q800
210	N4BP2L1	TMKLYSENLTL	73	91		Q565 & Q605
211	ARHGAP4	PLHCWVVLL	70	77		Q565 & Q655
212	DUSP22	CMLGDPVPT	57	86		Q565 & Q705
213	CD79b	PLDGGVAAA	45	65		Q585 & Q605
214	BTK	HLASEKVYAI	68	65		Q585 & Q705
215	CCL3	FLTKRGRQV	54	56		Q605 & Q655
216	PTPN6	RLGPVARTRV	48	68		Q605 & Q800
217	BTK	SLTTISTTL	92	84		Q655 & Q705
218	MAP4K1	IMAIELAEL	92	93		Q655 & Q800
219	ATP2A3	KMFVKGAPDSV	N.D.	106		Q705 & Q800
220	EVI2B	FLLQMMQICL	91	90	11	PE & APC
221	EVI2B	QMMQVCLHHL	87	65		PE & Q585
222	CSF3R	AMAQDPHSL	83	62		PE & Q605
223	NUP210	FLAPAKAVVYV	88	85		PE & Q705
224	BTK	KLANIQCLCL	89	51		PE & Q800
225	NUP210	TIMIHDLCLA	85	72		APC & Q565
226	IQGAP2	KIQRNLWTL	76	69		APC & Q585
227	ITGAM	VLRQEVAAPL	89	84		APC & Q605
228	NCF4	LLSDEDVALM	76	72		APC & Q655
229	EVI2B	ICLHHLPFWI	80	79		APC & Q705
230	EVI2B	VCLHHLPFWI	57	75		APC & Q800
231	ITGAM	RVLRQEVAAPL	74	79		Q565 & Q605
232	LRMP	LQSRGYSSL	72	70		Q565 & Q655
233	N4BP2L1	VMALENNYEV	60	72		Q565 & Q705
234	NUP210	TLLIGATIQVT	69	48		Q585 & Q605
235	PRKCB1	RIGQRQETV	63	63		Q585 & Q655
236	ARHGAP4	LLSPLHCWAV	77	70		Q585 & Q705
237	ZFP36L2	RLRPLCCTA	56	58		Q605 & Q655
238	NUP210	LLIGATIQV	59	71		Q605 & Q705
239	NUP210	TLLIGATIQV	68	81		Q605 & Q800
240	MPL	HLWVKNMFL	104	82	12	PE & APC
241	DOK2	TLLASSMSSQL	74	80		PE & Q585
242	NCF4	RLLSDEDVALM	77	68		PE & Q655
243	CD79b	TLRTGEVKWSV	69	62		PE & Q800
244	ITGB2	RLGGAALPRV	73	95		APC & Q565
245	BTK	LASEKVYTI	62	67		APC & Q585
246	ATP2A3	RLWTTTRPRV	68	68		APC & Q655
247	MAP4K1	GIMAIELAEL	81	81		APC & Q705
248	NUP210	LLIGATIQVT	71	46		APC & Q800
249	SEPT6	RLSCSSPRA	58	56		Q565 & Q605
250	IQGAP2	ILHSRTEFISV	66	70		Q565 & Q655
251	NUP210	LAPAKAVVYV	67	72		Q565 & Q705
252	LTB	DLSPGLPAA	62	61		Q585 & Q655
253	IQGAP2	KIQRNLRTL	66	39		Q585 & Q705
254	PIK3CD	KTGECCLYM	84	86		Q605 & Q655
255	NUP210	TLLIGATMQV	87	83		Q605 & Q705
256	FMNL1	SLWQLRAAV	86	91		Q605 & Q800
257	N4BP2L1	KLYSENLKL	93	90		Q655 & Q705
258	NCF4	SLLSLPVWV	80	87		Q655 & Q800
259	CORO1A	KLQATVQEL	89	81	13	PE & APC
260	SF1	SLWSSSPVA	86	102		PE & Q565
261	SF1	SLWSSSPMATT	75	83		PE & Q585
262	EVI2B	MMQICLHHL	97	74		PE & Q605
263	EVI2B	LLQMMQICL	87	87		PE & Q655
264	N4BP2L1	KIYSENLKL	83	81		PE & Q705
265	NUP210	CLAFPAPAKA	20	23		APC & Q565
266	NCF4	RLLSDEDVEL	85	93		APC & Q585
267	SYNGR1	SIFTWAGKAVL	81	46		APC & Q655
268	ITGAM	RVLRQEVEAPL	66	79		APC & Q705
269	CORO1A	GLWRHSPCA	114	62		Q565 & Q605
270	LRMP	SLLQSRGYSSL	69	72		Q565 & Q655
271	LTB	GLREREDLL	65	48		Q565 & Q705
272	NCF4	QLQGLQHNA	53	51		Q585 & Q605
273	HSPA6	FMTSSWWGA	65	67		Q585 & Q655
274	N4BP2L1	AVMALENNYEV	64	85		Q605 & Q655
275	NUP210	VTLLIGATIQV	63	78		Q605 & Q705
276	CD79b	LLSAEVQQHL	65	34		Q655 & Q800
277	CD48	KQDNSTYIMRV	N.D.	124		Q705 & Q800
278	DOK2	SMSSQLGRISL	86	76	14	PE & APC
279	NCF4	LLSDEDVEL	85	92		PE & Q585
280	NUP210	IMIHDLCLVFL	81	64		PE & Q605
281	NUP210	LLIGATMQVT	74	53		PE & Q655
282	SF1	SLWSSSPVATT	70	81		PE & Q705
283	LTB	GLCEREDLL	75	72		PE & Q800
284	PSD4	AQAEHSITRV	78	61		APC & Q605
285	CCL3	FLTKRGGQV	64	75		APC & Q705
286	NUP210	CLVFLAPAKA	50	67		Q565 & Q655
287	SF1	SLWSSSPMAT	88	100		Q585 & Q605
288	N4BP2L1	KIYSENLTL	91	94		Q585 & Q655
289	ARHGAP4	SLLSPLHCWA	86	81		Q605 & Q655
290	NCF4	RLLSDEDVAL	85	71		Q605 & Q705
291	EVI2B	GLPPPPPLL	80	85		Q605 & Q800
292	NUP210	CLVFPAPAKA	72	69		Q655 & Q705
293	NCF4	SLLNLPVWVLM	85	66		Q655 & Q800
294	MCM5	YLCDKVVPG	97	88	15	PE & APC
295	NUP210	VTLLIGATMQV	88	84		PE & Q565
296	ARHGAP4	LLSPLHCWA	50	65		PE & Q585
297	NCF4	LLSDEDVELM	83	69		PE & Q605
298	EVI2B	QMMQICLHHL	82	59		PE & Q655
299	SYNGR1	SIFTWAGQAVL	84	58		PE & Q705
300	FMNL1	SLWQLRAAVML	58	48		APC & Q565
301	NUP210	FLAPAKAVV	85	83		APC & Q585
302	CD79b	RTGEVKWSV	67	79		APC & Q605
303	BTK	LASEKVYAI	72	69		APC & Q655
304	CSF3R	AMARDPHSL	72	53		APC & Q800
305	NCF4	LLNLPVWVL	38	65		Q565 & Q605
306	FMNL1	RMTWRAQTM	79	38		Q565 & Q655
307	CCL3	FLTKRSGQV	69	77		Q565 & Q705
308	PRKCB1	SMSKEAVAI	68	65		Q585 & Q605
309	HSPA6	SMCRFSPLTL	61	69		Q585 & Q655
310	IQGAP2	NLWTLEQTGHV	60	82		Q585 & Q705

311	HSPA6	K C Q E V L A W L	82	30		Q605 & Q705
312	NUP210	T L L I G A T M Q V T	45	41		Q605 & Q800
313	PTPN6	T L S S R V C C R T	72	34		Q655 & Q705
314	NUP210	L V F L A P A K A V V	53	67		Q655 & Q800
315	NCF4	L L S D E D V A L M V	70	82	16	PE & APC
316	N4BP2L1	A L E N N Y E V L	71	63		PE & Q565
317	EVI2B	C L H H L P F W I	41	33		PE & Q585
318	PTPN6	S T V A S W L G P V	75	64		PE & Q605
319	CCL3	F L T K R S R Q V	64	76		PE & Q655
320	ARHGAP4	P L H C W A V L L	69	77		PE & Q800
321	PTPN6	T V A S R L G P V	73	61		APC & Q565
322	CCL3	F L T K R S G Q V C A	61	59		APC & Q605
323	SP110	L L E A P T G L V	75	69		APC & Q655
324	NCF4	V S L L S L P V W V	56	60		APC & Q800
325	ARHGAP15	K L G D C I W T Y P S	N.D.	105		Q565 & Q655
326	NUP210	M I H D L C L A F P A	N.D.	128		Q565 & Q705
327	PSD4	N L P D K M F L P G A	N.D.	124		Q585 & Q605
328	ARHGAP15	K L G D C I W T Y L S	N.D.	91		Q585 & Q655
329	HSPA6	G V F I Q V Y E V	N.D.	103		Q585 & Q705
330	LYN	L M F W S P S H S C A	N.D.	102		Q605 & Q705
331	MCM5	T L T N I A M R P G L	N.D.	74		Q605 & Q800
332	RASGRP2	Q V L D L R L P S G V	N.D.	107		Q655 & Q705
333	NUP210	A T A T P C W T W L L	N.D.	86		Q655 & Q800

$^{\alpha}$ Peptide sequences are provided with polymorphic residue in red

$^{\beta,\gamma}$ RS are normalized to the HLA-A2 high affinity CMV-pp65$_{NLV}$ peptide
