# Supplementary material for: High-Throughput Identification of Potential Minor Histocompatibility Antigens by MHC Tetramer-Based Screening: Feasibility and Limitations
Source: PLoS One. 2011 Aug 5;6(8):e22523. doi: 10.1371/journal.pone.0022523 (PMC3151248; doi:10.1371/journal.pone.0022523)
Supplement: Table S4 — Total pMHC tetramer-reactive T-cell populations revealed after pull down and two weeks of expansion. (PDF) [file pone.0022523.s006.pdf]

Supplementary Table IV.

Allo-SCT patient	pMHC tetramer group	Dual-color encoding	CD8+ T cell frequency	Peptide number	Allo-SCT patient	pMHC tetramer group	Dual-color encoding	CD8+ T cell frequency	Peptide number
APM4461	1	QD655 & PE	0,36	4	OBB1465	1	APC & QD565	0,09	7
12 weeks [α]	2	QD585 & QD605	0,31	37	12 weeks		PE & QD655	0,07	4
		QD605 & PE	0,04	25		2	QD585 & QD705	0,21	39
	3	APC & PE	0,15	46			PE & QD565	0,03	23
	4	APC & PE	0,06	68		4	PE & QD605	0,27	71
	5	PE & QD585	0,04	91			QD705 & QD585	0,04	84
		PE & QD605	n.t.	92		5	PE & QD655	0,17	93
		QD585 & QD655	0,18	104			QD585 & QD655	0,55	104
	6	PE & QD605	0,42	115		6	PE & QD800	0,25	118
	7	PE & APC	4,9	135			QD585 & QD705	0,04	128
	9	APC & QD565	0,04	185		8	PE & QD705	0,07	163
	11	QD585 & QD655	0,77	235		9	PE & QD800	0,35	184
	12	QD565 & APC	0,19	265		9	QD605 & QD655	0,15	195
	15	QD585 & QD655	0,6	309		11	QD605 & QD705	0,05	238
BDY3356	2	APC & PE	0,07	22		12	APC & QD565	0,04	244
16 weeks		QD605 & QD585	0,177	37			QD705 & QD605	0,06	255
	4	APC & PE	0,05	68		14	QD605 & QD800	0,06	291
	5	QD655 & QD585	0,215	104			QD655 & QD705	0,07	292
		APC & PE	0,11	89	JMO2750	1	QD585 & PE	0,27	2
		QD705 & QD655	0,17	109	18 weeks		QD705 & PE	0,02	5
	6	QD655 & QD800	0,27	133		4	QD585 & QD705	0,19	84
	7	QD655 & QD705	0,53	156			APC & PE	0,2	68
	9	APC & QD565	0,03	185		5	QD585 & QD655	0,2	104
	10	PE & QD585	0,05	203			QD655 & QD705	0,14	109
		QD705 & APC	0,05	208		6	QD585 & QD705	0,62	128
		QD655 & QD800	0,2	218		7	QD605 & QD655	0,52	153
	11	QD705 & APC	0,04	229			QD585 & QD605	0,15	150
LBK5266	2	PE & QD605	0,04	25			APC & PE	0,08	135
16 weeks	4	APC & PE	0,05	68		8	QD705 & PE	0,07	163
	8	PE & QD705	0,08	163		9	QD705 & APC	0,06	188
	10	QD585 & QD605	0,19	213			QD565 & APC	0,07	185
JVE3357	8	PE & QD705	0,08	163		11	QD585 & QD655	0,24	235
28 weeks	11	QD705 & APC	0,06	229		13	QD565 & APC	0,08	265
	12	QD605 & QD655	0,13	254		14	QD655 & QD705	0,34	292
	14	PE & QD655	0,15	281	MBF5177	2	QD605 & PE	0,04	25
					12 weeks	8	PE & QD705	0,1	163

[α] Allo-SCT patient PBMC samples were obtained at the indicated time point after DLI

Supplementary Table 4
